# Caste-, sex-, and age-dependent expression of immune-related genes in a Japanese subterranean termite, *Reticulitermes speratus*

**DOI:** 10.1371/journal.pone.0175417

**Published:** 2017-04-14

**Authors:** Yuki Mitaka, Kazuya Kobayashi, Kenji Matsuura

**Affiliations:** Laboratory of Insect Ecology, Graduate School of Agriculture, Kyoto University, Kyoto, Japan; Pusan National University, REPUBLIC OF KOREA

## Abstract

Insects protect themselves from microbial infections through innate immune responses, including pathogen recognition, phagocytosis, the activation of proteolytic cascades, and the synthesis of antimicrobial peptides. Termites, eusocial insects inhabiting microbe-rich wood, live in closely-related family groups that are susceptible to shared pathogen infections. To resist pathogenic infection, termite families have evolved diverse immune adaptations at both individual and societal levels, and a strategy of trade-offs between reproduction and immunity has been suggested. Although termite immune-inducible genes have been identified, few studies have investigated the differential expression of these genes between reproductive and neuter castes, and between sexes in each caste. In this study, we compared the expression levels of immune-related genes among castes, sexes, and ages in a Japanese subterranean termite, *Reticulitermes speratus*. Using RNA-seq, we found 197 immune-related genes, including 40 pattern recognition proteins, 97 signalling proteins, 60 effectors. Among these genes, 174 showed differential expression among castes. Comparing expression levels between males and females in each caste, we found sexually dimorphic expression of immune-related genes not only in reproductive castes, but also in neuter castes. Moreover, we identified age-related differential expression of 162 genes in male and/or female reproductives. In addition, although *R*. *speratus* is known to use the antibacterial peptide C-type lysozyme as an egg recognition pheromone, we determined that *R*. *speratus* has not only C-type, but also P-type and I-type lysozymes, as well as other termite species. Our transcriptomic analyses revealed immune response plasticity among all castes, and sex-biased expression of immune genes even in neuter castes, suggesting a sexual division of labor in the immune system of *R*. *speratus*. This study heightens the understanding of the evolution of antimicrobial strategies in eusocial insects, and of sexual roles in insect societies as a whole.

## Introduction

Insects are exposed to a variety of infectious microbes in their habitats throughout their life cycle [1]. Insects need to prevent infection in order to survive, and so they must recognize and eliminate pathogens if they become infected with any microbes. Social insects form colonies consisting of a large number of siblings, and thus they are all susceptible to the same pathogenic infections [2]. At the individual level, social insects eliminate pathogens by innate cellular and humoral immunity [3]. At the colony level, social insects have developed behavioral and social immunity such as allogrooming, trophallaxis, isolation, canibalism [2]. In addition, passive immuniazation, which is characterized by sharing immune factors between infected individuals and naive nestmates, is considered a type of social immunity in insect societies [4], although the mechanisms of passive immunization remains to be explored [5]. On the other hand, recent studies revealed an active immunization, which is characterized by social transfer to low-dose infectious pathogens and active up-regulation of nestmates’ immune systems, in ants [6] and termites [7].

Most insects actively defend themselves against fungal or bacterial infections, and the defense systems consist of various innate immune reactions, including phagocytosis [8], the activation of proteolytic cascades inducing local melanization and coagulation [1], and the biosynthesis of antimicrobial peptides by the fat body [9]. The insect immune system involves the following four steps: pathogen recognition, signal modulation, signal transduction, and pathogen elimination. Immune reactions are initiated after successful pathogen recognition. Microbial recognition occurs through conserved pathogen-associated molecular patterns (PAMPs) that are absent in the host. These PAMPs include β-1,3-glucan from fungi and lipopolysaccharides (LPSs) or peptidoglycans (PGNs) from bacteria [10]. PAMPs bind to host proteins generally referred to as pattern recognition proteins (PRPs). Insect genomes include many groups of PRPs, such as the C-type lectin-like domain (CTLD) superfamily, peptidoglycan recognition proteins (PGRPs), the apolipophorin III superfamily, and *β*-1,3(4)-glucan recognition proteins [10,11]. After the recognition of PAMPs by PRPs, signal modulation proteins such as serine proteases (SPs) and serine protease inhibitors (SPIs) amplify pathogen invasion signals, activating various lines of defense. These proteins are involved in hemolymph coagulation, antimicrobial peptide synthesis, and the activation of phenoloxidases that induce melanization of pathogen surfaces [12–14]. The insect immune response mainly relies on the immune deficiency (IMD), Toll, c-jun N-terminal kinase (JNK), and janus kinase/signal transduction and activator of transcription (JAK/STAT) immune signaling pathways, and these pathways induce the expression of effectors such as antimicrobial peptides [15]. Additionally, proteins belonging to the Ras superfamily, EF hand domain family, 14-3-3 proteins, low-density lipoprotein receptor-related proteins (LRPs), and the four-and-a-half LIM domain protein (FHL) family are involved in a variety of signal transduction pathways in insect immune responses [16–20]. Antimicrobial peptides and proteins are the final effectors produced by insects to eliminate pathogenic intruders. These molecules, including termicin [3,21,22], prolixicin [23], thaumatin-like protein [24–26], lysozyme [27], cathepsin, asparaginyl endopeptidase-like cysteine peptidase (AE-like CP), and metacaspase-like cysteine peptidase (MCA-like CP) [5,28,29], are upregulated upon pathogenic infection, and they directly attack pathogens.

Termites are eusocial insects that live inside wood. Damp wood termites live in microbe-rich habitats such as rotten wood [30]. They prevent pathogens from propagating and invading their nests by secreting strong antibiotic agents. For example, although the inner walls of termite nests are made from a mixture of excrement and saliva containing antimicrobial substances, the nest walls of the Formosan subterranean termite *Coptotermes formosanus* contain naphthalene [31]. On the other hand, some antimicrobial agents produced by the Japanese subterranean termite *Reticulitermes speratus* also function as pheromones. The antibacterial protein lysozyme is used as an egg recognition pheromone [32], and *n*-butyl-*n*-butyrate and 2-methyl-1-butanol, which have antifungal activities, are used as volatile components of queen pheromone [33,34]. Although cellular immune reactions have not been well studied in termites, the cellular encapsulation against the entomopathogenic fungus *Metarhizium anisopliae* was demonstrated in *R*. *flavipes* [35]. Humoral immune reactions have been investigated in some termite species. In *Mastotermes darwiniensis*, transferrin gene expression increases following infection with *M*. *anisopliae* [36]. The molecular evolution of pathogen recognition proteins, Gram-negative binding proteins (GNBPs), has been investigated in various termite species [37], and the previous study revealed that termite GNBPs also have *β*-(1,3)-glucanase activity, demonstrating that they fulfill the dual role of PRR and effector [38]. Moreover, 182 expression sequence tags were obtained from *R*. *flavipes* infected with *M*. *anisopliae* [39], and in *R*. *chinensis*, infected nestmates promoted the activity of antioxidant enzymes (superoxide dismutase (SOD) and catalase (CAT)) and the expressions of immune genes (*phenoloxidase*, *transferrin* and *termicin*). And two upregulated (60S ribosomal protein L23 and isocitrate dehydrogenase) and three downregulated (glutathione *S*-transferase D1, cuticle protein 19, and ubiquitin conjugating enzyme) immune proteins were validated by proteomic analyses [7]. Recently, Hussain et al. [29] investigated changes in the expression of immune-related genes in *C*. *formosanus* workers infected with common entomopathogenic fungi (*M*. *anisopliae* and *Beauveria bassiana*), Gram-positive bacteria (*Bacillus thuringiensis*), and Gram-negative bacteria (*Escherichia coli*), demonstrating that different types of genes were expressed following infection with different types of pathogens. However, few studies have investigated the differential expression of immune-inducible genes by caste, sex, and age in termites.

In this study, we annotated the immune-related genes of the Japanese subterranean termite, *R*. *speratus*, using the transcriptomic database that we constructed in a previous study [40]. Subsequently, we compared the expression levels of each gene among castes, between sexes in each caste, and among different ages in reproductives.

## Materials and methods

### RNA sequencing and *de novo* transcriptome assembly

Comprehensive transcriptome analysis of *R*. *speratus* was performed using high-throughput mRNA sequencing in the previous study [40]. To compare the expression levels of *R*. *speratus* immune-related genes among castes, ages, and between sexes, we analyzed transcriptomic database of both male and female alates, young primary kings (PKs) and primary queens (PQs), mature PKs and secondary queens (SQs), both male and female soldiers, and both male and female workers [40]. Alates were collected from three colonies in secondary forests in Kyoto, Japan, in the swarming season from April to May 2013. The alates were separated by sex, and then a male and female were randomly selected from each colony; five nestmate pairs were made for each colony and kept at 25°C under continuous darkness. After 6 months, male and female alates became young PKs and PQs, respectively, and then they were extracted from the incipient colonies. Kings and queens of *R*. *speratus* were collected from mature termite colonies in secondary forests in Kyoto and Shiga, Japan, during the reproductive season from July to October 2013. We found royal chambers of these colonies and extracted mature PKs and SQs together with workers and soldiers. Male and female workers and soldiers were separated based on the caudal sternite configuration using a stereoscope. No specific permits were required for the described field studies and no specific permissions were required for the locations/activities for insect sampling because they are public lands and are not privately owned or protected in any way. These field studies did not involve endangered or protected species.

Total RNA was extracted from the whole body of each individual of each reproductive caste (alates, kings, and queens) using an RNeasy mini kit (Qiagen), using the standardized instructions from the manufacturer. As for workers and soldiers, we pooled 10 individuals of each sex to extract a sufficient amount of RNA, and we performed RNA-seq analysis on a total of 60 samples. Following previously described procedures [41] for first-strand cDNA synthesis, RNA sequencing was performed using the Illumina HiSeq 2000 at the Okinawa Institute of Science and Technology Graduate University. For further details of the methods, see Mitaka et al. (2016) [40]. Sequence data was deposited in the DNA Data Bank of Japan (DDBJ) under the BioProject PRJDB3531, which contains links and access to sampling data through the BioSample SAMD00026264—SAMD00026323 and the Sequence Read Archive DRR030795—DRR030854.

The raw sequencing reads were trimmed by removing adapter sequences. Preprocessing was performed in DDBJ Read Annotation Pipeline. Bases with a quality score of < 20 were trimmed from the 5’ and 3’ ends of each read. After the trimming, reads with a high percentage (> 30%) of low quality bases (< 15) and short reads (< 25 bp) were discarded, and the remaining reads were used for an assembly. The remaining reads from all of the samples were assembled *de novo* using Trinity version trinityrnaseq_r2012–04–27 [42,43], which generates transcriptomic assemblies from short read sequences using the de Bruijn graph algorithm. All parameters selected to run Trinity were default parameters (k-mer length = 25-mers) except max_reads_per_loop, which was set to 1,500,000. Illumina sequencing yielded a total of 729 M read pairs with a mean length of 93 bp for each short read, and the GC content was 41%. The transcriptome yielded a total of 856 Mbp of RNA sequences, 1,144,272 contigs with a minimum length of 201 bp, a maximum length of 35,267 bp, a mean length of 748 bp, and a N50 value of 1,296 bp [40].

We also searched open reading frames (ORFs) in the assembled sequences using transcriptsToOrfs version 0.0.2 and detected 156,276 ORFs with a total length of 76,684,150 amino acids (aa), a minimum length of 100 aa, a maximum length of 11,369 aa, a mean length of 490.70 aa, and a N50 length of 584 aa. Based on the subsequent homology search of the known amino acid sequence data from a termite (*Zootermopsis nevadensis*), an aphid (*Acyrthosiphon pisum*), a honeybee (*Apis mellifera*), a beetle (*Tribolium castaneum*), a silkmoth (*Bombyx mori*), and a fruit fly (*Drosophila melanogaster*), 10,238 protein-coding transcripts were inferred in *R*. *speratus* [40].

### Annotation of immune-related proteins

Targets of immune-related genes were selected based on a previous study investigating immune-inducible genes in *C*. *formosanus* [29]. Pattern recognition proteins (PRPs) included C-type lectins (CTLs), lipopolysaccharide-binding proteins (LPSBPs), agglucetins, brevicans, laminins, GNBPs, apolipophorin III, PGRPs, and endo-β-1,4-glucanase. Signalling proteins included SPs, SPIs, and prophenoloxidase activating factor, 14-3-3 proteins, calpains, minor histocompatibility antigens (MHAs), LRPs, and FHLs. Also, effectors contained antimicrobial proteins such as carboxypeptidases (CPases), cathepsins, lysozymes, metacaspase-like cysteine peptidases (MCA-like CPs), asparaginyl endopeptidase-like cysteine peptidase (AE-like CPs), lysosomal Pro-X carboxypeptidases, prolixicin antimicrobial proteins, transferrins, and termicins, cysteine-rich proteins (CRPs), ferritins, melanotransferrins, venom allergens, and thaumatin-like proteins.

Peptide sequences of immune-related proteins of the following species were obtained from the National Center for Biotechnology Information (NCBI) (http://www.ncbi.nlm.nih.gov/), and used as BLAST queries for our peptide database with an E-value cutoff of 1E−20: *Zootermopsis nevadensis*, *Cryptotermes secundus*, *C*. *formosanus*, *R*. *flavipes*, *R*. *chinensis*, *Nasutitermes comatus*, *Periplaneta americana*, *Eupolyphaga sinensis*, *Tribolium castaneum*, *Apis mellifera*, *Bombyx mori*, and *Drosophila melanogaster* (S1 Table).

### Abundance estimation and differential expression analyses

Expression levels of immune-related genes were estimated by RSEM version 1.2.8 software [44] separately for the filtered reads from each sample. Raw read counts generated by RSEM were normalized with the trimmed mean of M-value normalization method [45]. Subsequently, these read counts were used for differential expression analyses among castes and ages, and between sexes using the edgeR software package v3.4.2 [46]. All statistical analyses were performed using the R package v3.0.3 and heatmaps were generated with heatmap.2 in the gplots package.

## Results

### Pattern recognition proteins

**Fig 1:**
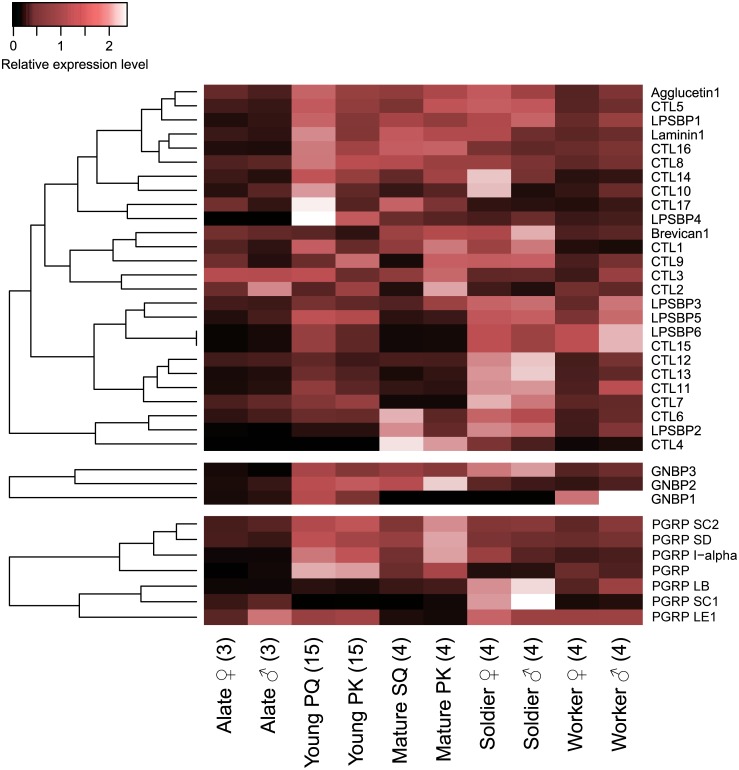
Differential expressions of CTLDs, GNBPs and PGRPs among castes. These heatmaps indicate the differential expression of 29 genes belonging to the CTLD superfamily, the GNBP family and PGRPs among termite castes (PQ: primary queen, PK: primary king, SQ: secondary queen). Relative expression level indicates the mean normalized count per million (CPM), ranging from black (scaled expression of 0) to white (scaled expression of 2.5). The tree at the left corresponds to hierarchical clustering of cluster-averaged expression. Numbers in parentheses after caste names refer to the numbers of biological replicates. Ten individuals were pooled for each sex of worker and soldier to obtain sufficient amounts of RNA, while single individuals were used for RNA extraction in the other castes. CTL: C-type lectin-like domain protein, LPSBP: lipopolysaccharide-binding protein, GNBP: Gram-negative binding protein, PGRP: peptidoglycan recognition protein.

CTLD-containing proteins include CTLs, LPSBPs, agglucetins, brevicans, and laminins [47]. Through a BLAST search querying the amino acid sequences of various insect species (S1 Table), we found 26 CTLD-coding genes (Table 1). Among these genes, the expression levels of six genes (brevican1, CTL7, and CTL11–14) in soldiers were more than 1.2 times as high as those in other castes (false discovery rate (FDR) < 0.05; Fig 1 and S2 Table). The expressions of CTL17 and LPSBP4 in young reproductives (PKs and PQs) were more than twice as high as those in other castes, and those of CTL9 and LPSBP1 in young reproductives, mature reproductives (PKs and SQs), and soldiers were more than 1.3 times as high as those in alates and workers (FDR < 0.05; Fig 1 and S2 Table). Additionally, the expression levels of 16 genes differed significantly between reproductive statuses, *i*.*e*. reproductive castes (alates, young PKs and PQs, and mature PKs and SQs) and neuter castes (soldiers and workers), and 12 genes showed sexually dimorphic expression patterns regardless of reproductive status (reproductive status: FDR < 0.05, sex nested by reproductive status: FDR < 0.05, Table 1 and S5 Table). Comparing the expression levels between sexes in each caste, the expression of four genes (brevican 1, CTL10, CTL17, and laminin 1) in young PQs were more than twice as high as those in young PKs, but two other genes (CTL7 and CTL9) showed the opposite pattern (FDR < 0.05; S3 Table). CTL6, CTL17, and LPBP2 were more highly expressed in mature SQs than mature PKs, but CTL9 showed the opposite pattern (FDR < 0.05; S3 Table). Also, CTL10 and CTL14 expression levels in female soldiers were more than 2.5 times as high as those male soldiers, and CTL3 showed male-biased expression in workers (FDR < 0.05; S3 Table). Among male reproductives (male alates and young and mature PKs), CTL12 expression showed the highest level in alates. However, the expression levels of 8 genes (CTL7, CTL9, CTL11, CTL13, CTL15, and LPSBP4–6) peaked in young PKs, and those of 5 genes (CTL1, CTL4, CTL16, LPBP2, and laminin1) peaked in mature PKs (FDR < 0.05; S4 Table). In female reproductives (female alates and young PQs), brevican 1 and CTLD4 expression levels in alates were more than 1.2 times as high as those in young PQs, but the expressions of 9 other genes (CTL11, CTL13, CTL15, CTL16, LPSBP1, LPSBP4, LPSBP5, LPSBP6, and laminin 1) in young PQs were more than three times as high as those in alates (FDR < 0.05; S4 Table).

**Table 1:** Number of caste-, sex-, and age-specific immune-related genes.

Category	Gene group	Total	Caste	Sex	Age	R/N	Sex (R/N)
PRP	CTLD-containing protein	26	22	11	17	16	12
PRP	Apolipophorin III	3	3	1	3	2	0
PRP	Endo-β-1,4-glucanase	1	1	1	1	1	1
PRP	PGRP	7	5	1	7	3	4
PRP*	GNBP	3	3	1	3	2	2
S	SP	70	63	21	58	38	23
S	SPI	7	7	2	6	4	1
S	proPO activating factor	1	1	0	1	1	0
S	14-3-3 protein	3	2	1	3	1	0
S	Calpain	5	5	1	4	2	1
S	MHA	2	2	1	2	1	0
S	LRP	8	8	5	7	5	4
S	FHL	1	1	0	1	1	0
E	CPase	11	11	3	11	5	0
E	Cathepsin	10	7	3	6	1	2
E	Lysozyme	9	7	5	8	6	4
E	MC-like CP	2	2	1	2	1	0
E	AE-like CP	1	1	1	1	1	0
E	Lysosomal Pro-X CPase	1	1	0	1	0	0
E	Prolixicin	1	1	1	1	1	1
E	Transferrin	3	3	3	3	1	2
E	Termicin	1	1	1	1	1	1
E	CRP	14	11	7	11	8	5
E	Ferritin	4	3	1	2	0	1
E	Melanotransferrin	1	1	0	1	0	0
E	Venom allergen	1	1	1	1	1	0
E	Thaumatin-like protein	1	1	0	0	1	0
Total number of genes		197	174	73	162	104	64
Ratio		100%	88%	37%	82%	53%	32%

This table shows the total number of genes, and the number of genes showing significant caste-specific expression pattern (Caste), sexual differences in each caste (Sex), age-dependent expression changes in male and/or female reproductives (Age), reproductive status-specific differences (reproductive or neuter caste, R/N), and sexual differences nested by reproductive status (Sex (R/N)) in each protein group (FDR < 0.05). PRP: pattern recognition protein, S: Signalling protein.

E: Effector,

*termite GNBPs function the dual role as the pattern recognition protein and as the effector.

proPO activating factor: Prophenoloxidase activating factor, Lysosomal Pro-X CPase: Lysosomal Pro-X Carboxypeptidase.

Seven PGRPs were annotated (Table 1 and S1 Table). Five genes (PGRP I-alpha, PGRP LB, PGRP LE1, PGRP SC1, PGRP) showed caste-specific expression patterns; for example, the expression levels of PGRP LB and PGRP SC1 in soldiers were more than twice as high as that in other castes (FDR < 0.001, Fig 1 and S2 Table). Comparing the expression level between female and male in each caste, only PGRP SC1 showed the king-biased expression pattern in mature reproductives (FDR < 0.001, S3 Table). Also, the expression levels of three genes (PGRP LB, PGRP LE1 and PGRP SC1) significantly differed between reproductive and neuter castes, and four genes (PGRP I-alpha, PGRP LE1, PGRP SC1 and PGRP SC2) showed sexually dimorphic expression regardless of reproductive status (reproductive status: FDR < 0.001, sex nested by reproductive status: FDR < 0.05, S5 Table). In male reproductives, the expression levels of four genes (PGRP I-alpha, PGRP LB, PGRP SC2 and PGRP SD) rose through the king’s life (FDR < 0.001, S4 Table). In female reproducitves, the expressions of two genes (PGRP I-alpha and PGRP) rose through the queen’s life, but that of PGRP SC1 showed the opposite pattern (FDR < 0.001, S4 Table).

Three apolipophorin III genes were predicted for *R*. *speratus* (Table 1 and S1 Table), and apolipophorin III-1 expression was higher in both young and mature reproductives than in other castes. The remaining two apolipophorin III genes were expressed the highest in young reproductives (FDR< 0.05; S1 Fig and S2 Table). Apolipophorin III-1 and 3 showed differential expression levels between reproductive and neuter castes (FDR < 0.05, Table 1 and S5 Table). The expression level of apolipophorin III-3 in mature SQs was more than twice as high as that in mature PKs (FDR < 0.05, S3 Table). Also, in male reproductives, apolipophorin III-1 expression showed the highest level in mature PKs, and apolipophorin III-2 and 3 showed in young PKs (FDR < 0.05; S4 Table). In contrast, in female reproductives, apolipophorin III-1 and 2 expression levels in female alates were seven times as high as young PQs (FDR < 0.05; S4 Table).

An endo-β-1,4-glucanase was also annotated (Table 1), and it showed high sequence similarity to that of *C*. *formosanus* (Identities = 96%; S1 Table). Although this gene was expressed in alates, young reproductives, and soldiers (FDR < 0.05; S3 Fig and S2 Table), it was little expressed in mature reproductives and soldiers in comparison to other castes. Also, this gene showed expression differences between reproductive statuses and between sexes (reproductive status: FDR < 0.001, sex nested by reproductive status: FDR < 0.001, Table 1 and S5 Table). This gene was expressed higher in young PQs than in young PKs (FDR < 0.05; S3 Table). The expression level decreased with age in male reproductives (FDR < 0.01; S4 Table), but increased with age in female reproductives (FDR < 0.05, S4 Table).

Three GNBP genes were annotated (Table 1 and S1 Table). The expression level of GNBP3 in soldiers was more than 1.5 times as high as those in other castes (FDR < 0.001; Fig 1 and S2 Table). Also, the expression levels of GNBP1 and GNBP3 differed between reproductive and neuter castes, and GNBP1 and GNBP2 showed sexually dimorphic expression patterns regardless of reproductive status (reproductive status: FDR < 0.001, sex nested by reproductive status: FDR < 0.05, Table 1 and S5 Table). In male reproducitve castes, the expression of GNBP1 peaked in young PK, and those of GNBP2 and GNBP3 peaked in mature PK (FDR < 0.001, S4 Table). In female reproducitve castes, the expressions of GNBP1 and GNBP3 rose through the PQ’s life (FDR < 0.001, S4 Table).

### Signalling proteins

**Fig 2:**
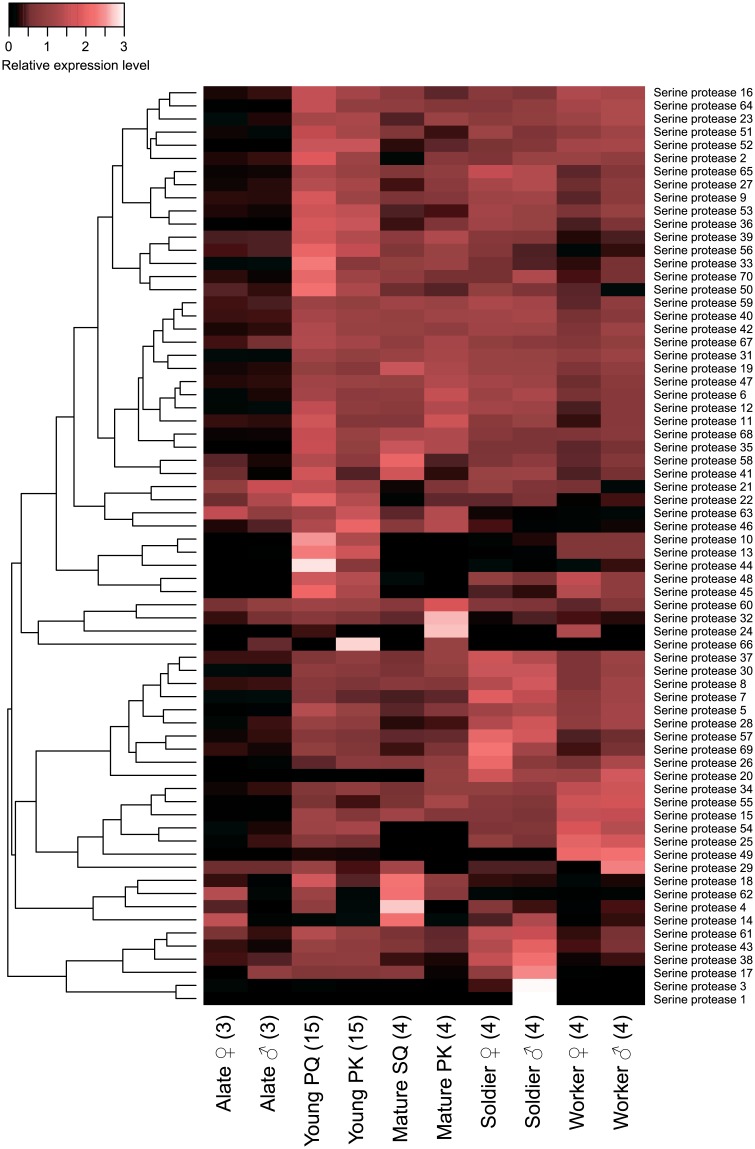
Differential expression of serine proteases among castes. The heatmap indicates the differential expression of 70 serine protease transcripts among castes. Abbreviations are as indicated in Fig 1. Relative expression level indicates the mean normalized CPM, ranging from black (scaled expression of 0) to white (scaled expression of 3.0). The tree at the left corresponds to hierarchical clustering of cluster-averaged expression.

Seventy SPs were annotated through a BLAST search against sequences from various insects (Table 1 and S1 Table). Fourteen SPs (SP2, SP9, SP10, SP13, SP21, SP22, SP33, SP36, SP44, SP50, SP51, SP53, SP56, and SP70) showed the highest expression levels in young reproductives, 11 SPs (SP3, SP7, SP8, SP26, SP28, SP30, SP37, SP38, SP43, SP57, and SP69) in soldiers, five SPs (SP4, SP14, SP18, SP58, and SP62) in mature reproductives, and five SPs (SP15, SP25, SP34, SP49, and SP55) in workers (FDR < 0.05; Fig 2 and S2 Table). Thirty-eight genes showed differential expression between reproductive statuses, and 23 genes showed sexually dimorphic expression (reproductive status: FDR < 0.05, sex nested by reproductive status: FDR < 0.05, Table 1 and S5 Table). When comparing expression levels between sexes within each caste, significant sexually dimorphic expression was found for four SPs in alates (female > male: SP18, SP41, and SP62; male > female: SP66; FDR < 0.05; S3 Table), 10 SPs in young reproductives (PQ > PK: SP4, SP11, SP18, SP41, SP44, and SP62; PK > PQ: SP37, SP46, SP63, and SP66; FDR < 0.01; S3 Table), 12 SPs in mature reproductives (SQ > PK: SP4, SP14, SP18, SP41, SP48, SP58, and SP62; PK > SQ: SP2, SP20, SP32, SP63, and SP66; FDR < 0.05; S3 Table), and six SPs in soldiers (female > male: SP46 and SP69; male > female: SP1, SP3, SP10, and SP70; FDR < 0.05; S3 Table). In male reproductives, while SP21 expression peaked in male alates (FDR < 0.05; S4 Table), the expressions of 32 SPs (SP4, SP5, SP9, SP10, SP13, SP16, SP23, SP25, SP27, SP28, SP36, SP38, SP42–46, SP48–54, SP57, SP58, SP61, SP64–66, SP69, and SP70) peaked in young PKs, and 22 SPs (SP6, SP7, SP11, SP12, SP15, SP18–20, SP30–35, SP37, SP39, SP40, SP47, SP55, SP60, SP62, and SP68) peaked in mature PKs (FDR < 0.05; S4 Table). In female reproductives, the expression levels of SP14, SP62, and SP63 peaked in alates, but those of 38 other SPs (SP2, SP5–7, SP9, SP10, SP12, SP13, SP15, SP18–20, SP23, SP25, SP27, SP28, SP30, SP31, SP33–36, SP40, SP42, SP44, SP45, SP47–49, SP51–55, SP57, SP64, SP65, and SP68) peaked in young PQs (FDR < 0.05; S4 Table).

Seven SPIs were annotated (Table 1 and S1 Table). While Kazal-type SPI domain-containing protein 1 and SPI2 showed the highest expression in soldiers, SPI3, dipetalogastin 1, and dipetalogastin 2 showed in young reproductives, and SPI4 showed in young reproductives and soldiers (FDR < 0.05; S1 Fig and S2 Table). SPI1 was expressed in all castes except for alates (FDR < 0.01; S1 Fig and S2 Table). Four genes exhibited differential expression between reproductive and neuter castes, and one gene exhibited sexually dimorphic expression (reproductive status: FDR < 0.05, sex nested by reproductive status: FDR < 0.05, Table 1 and S5 Table). Comparing between young reproductives, the expression of dipetalogastin 1 was slightly higher in PKs than that in PQs, but the expression of dipetalogastin 2 in PQs was twice as high as that in PKs (FDR < 0.01; S3 Table). In male reproductives, the expression level of SPI1 increased with age, but those of SPI3, SPI4, and dipetalogastin 1 peaked in young PKs (FDR < 0.01; S4 Table).

One prophenoloxidase activating factor was annotated (Table 1 and S1 Table) and was highly expressed in young reproductives and soldiers than other castes (FDR < 0.01; S3 Fig and S2 Table). The expression level of this gene differed between reproductive and neuter castes (FDR < 0.05, Table 1 and S5 Table). In both male and female reproductives, its expression exhibited the highest level in young reproductives among all age classes (FDR < 0.01; S4 Table).

Three 14-3-3 proteins were annotated (Table 1 and S1 Table), of which the expression of 14-3-3 protein 1 in young reproductives was 1.5 times as high as that in other castes, and the expression of 14-3-3 protein 2 in both young reproductives and soldiers was twice as high as that in other castes (FDR < 0.01; S2 Fig and S2 Table). The expression level of 14-3-3 protein 2 differed significantly between reproductive and neuter castes (FDR < 0.05, Table 1 and S5 Table). Comparing the expression level between sexes, 14-3-3 protein 3 expression was doubly higher in young PQs and mature SQs than young and mature PKs, respectively (FDR < 0.05; S3 Table). In male reproductives, all genes were the most highly expressed in young PKs among all age classes, but in female reproductives, 14-3-3 protein 1 and 14-3-3 protein 2 were expressed at the highest levels in young PQs (FDR < 0.01; S4 Table).

Five calpains were annotated (Table 1 and S1 Table). Calpain 1 showed the highest expression in young reproductives among all castes (FDR < 0.01; S1 Fig and S2 Table). While the expression levels of calpain 2 and calpain 3 were higher in young reproductives and soldiers than other castes, that of calpain 4 was higher in young and mature reproductives (FDR < 0.01; S1 Fig and S2 Table). Calpain 5 expression showed the highest level in mature reproductives (FDR < 0.01; S1 Fig and S2 Table). Calpains 2 and 4 showed significant expression differences between reproductive and neuter castes, and calpain 4 expression was sexually dimorphic (reproductive status: FDR < 0.001, sex nested by reproductive status: FDR < 0.001, Table 1 and S5 Table). Only the expression level of calpain 3 in young PQs was three times as high as that in young PKs (FDR < 0.01; S3 Table). Among all age classes in male reproductives, the expression of calpain 1 peaked in young PKs, but the expressions of calpain 4 and calpain 5 peaked in mature PKs (FDR < 0.01; S4 Table). On the other hand, in female reproductives, the expressions of calpain 1 and calpain 2 increased with age (FDR < 0.05; S4 Table).

Two MHAs were annotated (Table 1) through a BLAST search against the MHA sequences of *Z*. *nevadensis*, and showed high sequence similarities (Identities ≥ 77%, Positives ≥ 85%; S1 Table). MHA1 was expressed particularly in young reproductives and soldiers, and MHA2 was remarkably expressed in soldiers (FDR < 0.01; S2 Fig and S2 Table). The expression level of MHA2 in mature PKs was 2.5 times as high as that in SQs (FDR < 0.01; S3 Table). MHA2 was also differentially expressed between reproductive and neuter castes (FDR < 0.001, Table 1 and S5 Table). In male reproductives, MHA2 expression showed the highest level in mature PKs among all age clsasses, and in female reproductives, the expressions of MHA1 and MHA2 showed the highest level in young PQs (FDR < 0.05; S4 Table).

Eight LRPs were annotated (Table 1) through a BLAST search against the LRP sequences of *Z*. *nevadensis* (S1 Table). Each LRP of *R*. *speratus* exhibited the caste-specific expression pattern (FDR < 0.05; S2 Fig and S2 Table). Five LRPs exhibited differential expression between reproductive and neuter castes, and four LRPs exhibited sexually dimorphic expression (reproductive status: FDR < 0.05, sex nested by reproductive status: FDR < 0.05, Table 1 and S5 Table). Although the expression levels of LRP2 and LRP8 in mature PKs were more than twice as high as those in SQs, LRP3 and LRP6 showed the female-biased expression patterns in mature reproductives. Also, LRP5 was more highly expressed in young PQs and female soldiers than young PKs and male soldiers (FDR < 0.05; S3 Table). As for male reproductives, the expression of LRP8 decreased with age, but LRP1 and LRP2 showed the opposite pattern (FDR < 0.05; S4 Table). The expression levels of LRP3, LRP6, and LRP7 peaked in young PKs (FDR < 0.01; S4 Table).

One FHL was annotated in *R*. *speratus* (Table 1), and the sequence showed high similarity to that of *A*. *mellifera* (Identities = 93%; S1 Table). This gene was remarkably expressed in soldiers (FDR < 0.01; S2 Table), and its expression differed significantly between reproductive and neuter castes (FDR < 0.001, Table 1 and S5 Table). There was no sexually dimorphic expression among the castes (FDR > 0.05; S3 Table). In male reproductives, the expression of this gene showed the highest level in young PKs among all age classes (FDR < 0.01; S4 Table).

### Effectors

Eleven CPases were annotated in *R*. *speratus* through a BLAST search against *Z*. *nevadensis* sequences (Table 1 and S1 Table). CPase1, CPase3, CPase4, and CPase11 were remarkably expressed in young reproductives than other castes, and CPase8 was higher in young and mature reproductives than other castes (FDR < 0.05; S3 Fig and S2 Table). Five genes were differentially expressed between reproductive and neuter castes (FDR < 0.05, Table 1 and S5 Table). Comparing expression levels between males and females in each caste, the expression of CPase2 in mature PKs was more than 2.5 times as high as that in SQs, but CPase3 showed the opposite pattern (FDR < 0.05; S3 Table). CPase8 was doubly expressed in female soldiers than males (FDR < 0.05; S3 Table). In male reproductives, the expression of seven genes (CPase1–4, CPase6, CPase10, and CPase11) peaked in young PKs among all PK’s ages, and those of three genes (CPase5, CPase7, and CPase8) peaked in mature PKs (FDR < 0.01; S4 Table). In female reproductives, the expression of CPase9 peaked in female alates, but those of seven other genes (CPase1–4, CPase6, CPase8, and CPase11) peaked in young PQs (FDR < 0.01; S4 Table).

**Fig 3:**
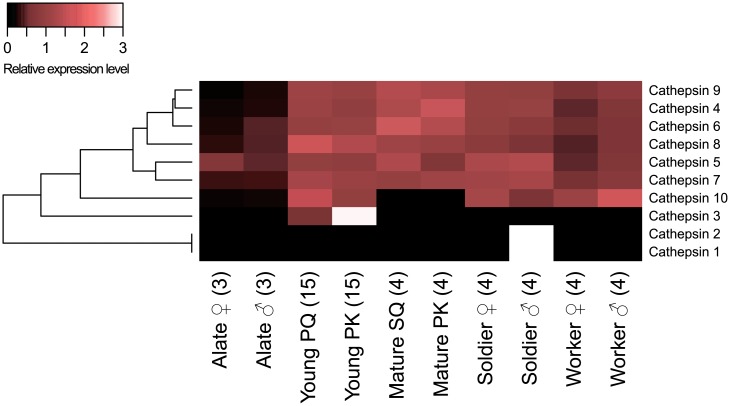
Differential expression of cathepsins among castes. The heatmap indicates the differential expression of 10 cathepsin transcripts among castes. Abbreviations are as indicated in Fig 1. Relative expression level indicates the mean normalized CPM, ranging from black (scaled expression of 0) to white (scaled expression of 3.0). The tree at the left corresponds to hierarchical clustering of cluster-averaged expression.

Ten cathepsins were predicted (Table 1 and S1 Table). Cathepsin 4, cathepsin 6, and cathepsin 9 showed the mature reproductives-specific expressions, and cathepsin 8 showed the young reproductives-specific expressions (FDR < 0.05; Fig 3 and S2 Table). Cathepsin 10’s expression differed between reproductive and neuter castes, and cathepsin 1 and 2 showed sexually dimorphic expression (reproductive status: FDR < 0.001, sex nested by reproductive status: FDR < 0.001, Table 1 and S5 Table). When comparing expression levels between sexes in each caste, cathepsin 1 and cathepsin 2 showed male-biased expression in soldiers, and the expression of cathepsin 3 in young PKs was four times as high as that in PQs (FDR < 0.05; S3 Table). In male reproductives, the expression levels of four genes (cathepsin 4, cathepsin 6, cathepsin 7, and cathepsin 9) rose with the king’s age, and those of two genes (cathepsin 8 and cathepsin 10) peaked in young PKs (FDR < 0.05; S4 Table). In female reproductives, the expression levels of five genes (cathepsin 4, cathepsin 6, cathepsin 7, cathepsin 8, and cathepsin 9) rose with the queen’s age (FDR < 0.05; S4 Table).

**Fig 4:**
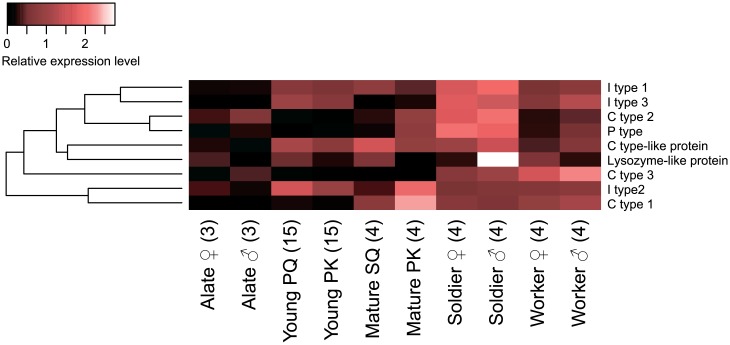
Differential expression of lysozymes among castes. The heatmap indicates the differential expression of 9 lysozyme transcripts among castes. Abbreviations are as indicated in Fig 1. Relative expression level indicates the mean normalized CPM, ranging from black (scaled expression of 0) to white (scaled expression of 2.75). The tree at the left corresponds to hierarchical clustering of cluster-averaged expression.

Previous studies have discovered two lysozyme genes in *R*. *speratus* [32,48,49]. In this study, we annotated a total of nine lysozyme (or lysozyme-like protein) genes (Table 1 and S1 Table), which include three C-type lysozymes, one C-type-like lysozyme, three I-type lysozymes, one P-type, and one lysozyme-like protein. The phylogeny inferred from the amino acid sequences of lysozymes in Blattodea (termites and cockroaches) was consistent with the previously known topology of the lysozyme types (S4 Fig). C-type 1 expression showed the highest level in mature reproductives among all castes; P-type, C-type 2, I-type 1, and I-type 3 showed in soldiers; and C-type 3 showed in workers (FDR < 0.01; Fig 4 and S2 Table). The expression levels of six genes differed between reproductive and neuter castes, and, of those, four genes showed sexual dimorphism (reproductive status: FDR < 0.05, sex nested by reproductive status: FDR < 0.05, Table 1 and S5 Table). Comparing expression levels between sexes in each caste, P-type lysozyme expression in young PKs was four times as high as that in PQs (FDR < 0.01; S3 Table). Although the expression levels of P-type, C-type 2, and I-type 2 were higher in mature PKs than in SQs, those of I-type 1 and C-type-like protein showed the opposite pattern (FDR < 0.05; S3 Table). In male reproductives, the expression levels of C-type 1, C-type 2, C-type-like protein, and I-type 2 increased with the PK’s age, but those of I-type 1 and I-type 3 peaked in young PKs (FDR < 0.05; S4 Table). In female reproductives, the expression levels of P-type, C-type 2, and C-type 3 decreased with the PQ’s age, but those of C-type 1 and I-type 3 showed the opposite pattern (FDR < 0.05; S4 Table).

Two MCA-like CPs were predicted (Table 1 and S1 Table). MCA-like CP1 was remarkably expressed in young reproductives, soldiers, and workers, but MCA-like CP2 was remarkably expressed in alates, soldiers, and workers (FDR < 0.01; S3 Fig and S2 Table). MCA-like CP2 exhibited differential expression between reproductive and neuter castes (FDR < 0.001, S5 Table). Also, the MCA-like CP2’s expression in young PKs was seven times as high as that in PQs (FDR < 0.05; S3 Table). Among age classes in male reproductives, the expression of MCA-like CP1 peaked in young PKs, and that of MCA-like CP2 peaked in male alates (FDR < 0.05; S4 Table). On the other hand, in female reproductives, MCA-like CP1 was higher in young PQs than alates, but MCA-like CP2 showed the opposite pattern (FDR < 0.05; S4 Table).

One AE-like CP was annotated (Table 1) through a BLAST search against the AE-like CP sequence of *C*. *formosanus* (S1 Table). AE-like CP1 was more expressed in soldiers than other castes (FDR < 0.01; S2 Table), and its expression level differed between reproductive and neuter castes (FDR < 0.001, Table 1 and S5 Table). The expression of this gene in young PKs was three times as high as that in young PQs (FDR < 0.05; S3 Table). It was also higher in female alates than young PQs (FDR < 0.01; S4 Table).

One lysosomal Pro-X carboxypeptidase was annotated (Table 1) through a BLAST search against the *Z*. *nevadensis* sequence (S1 Table). This gene was highly expressed in soldiers, and young and mature reproductives (FDR < 0.01; S2 Table), and it was higher in young PQs than in female alates (FDR < 0.05; S4 Table).

One prolixicin antimicrobial protein was annotated (Table 1 and S1 Table) and was remarkably expressed in mature reproductives and soldiers than other castes (FDR < 0.01; S2 Table). Its expression level also differed between reproductive and neuter castes, and showed sexual dimorphism (reproductive status: FDR < 0.001, sex nested by reproductive status: FDR < 0.001, Table 1 and S5 Table). The expression of this gene in SQs was tenfold as high as that in PKs (FDR < 0.01; S3 Table). Among all age classes in male reproductives, the expression level peaked in mature PKs (FDR < 0.01; S4 Table).

Among three predicted transferrins (Table 1 and S1 Table), transferrin 1 showed the highest expression in mature reproductives and workers among all castes, and transferrin 2 and 3 showed the highest expression in mature reproductives (FDR < 0.01; S3 Fig and S2 Table). The expression level of transferrin 1 differed between reproductive and neuter castes, and those of transferrin1 and 3 showed significant sexual dimorphism (reproductive status: FDR < 0.001, sex nested by reproductive status: FDR < 0.001, Table 1 and S5 Table). Comparing expression levels between sexes in each caste, the transferrin 2’s expression level was higher in young PKs than PQs, but transferrin 3 showed the opposite pattern (FDR < 0.05; S3 Table). Also, transferrin 1 expression was higher in mature PKs than SQs, but transferrin 3 was higher in mature SQs (FDR < 0.05; S3 Table). In male reproductives, transferrin 1 and transferrin 2 were highest in mature PKs, and in female reproductives, transferrin 1 and transferrin 3 were higher in young PQs than female alates (FDR < 0.05; S4 Table).

One termicin was also annotated (Table 1 and S1 Table) and was higher in mature reproductives (FDR < 0.01; S3 Fig and S2 Table). The expression levels of termicin 1 differed between reproductive and neuter castes, and it exhibited sexually dimorphic expression (reproductive status: FDR < 0.01, sex nested by reproductive status: FDR < 0.001, Table 1 and S5 Table). Termicin 1 expression was higher in mature SQs than PKs (FDR < 0.01; S3 Table). In male reproductives, the expression level of termicin 1 peaked in mature PKs (FDR < 0.01; S4 Table). In female reproductives, termicin 1 increased with age (FDR < 0.05; S4 Table).

We found 14 CRPs (Table 1 and S1 Table). CRP3 and CRP10 showed lower expression level only in alates, and CRP8 and CRP12 showed the highest expression level in mature reproductives among all castes (FDR < 0.01; S3 Fig and S2 Table). Eight genes showed significant expression differences between reproductive and neuter castes, and five genes showed sexually dimorphic expression (reproductive status: FDR < 0.05, sex nested by reproductive status: FDR < 0.05, Table 1 and S5 Table). Although CRP5 expression was higher in young PKs than PQs, CRP8, CRP11, and CRP12 showed the opposite pattern (FDR < 0.05; S3 Table). The expression levels of CRP1, CRP8, CRP9, and CRP12 in SQs were more than twice as high as those in mature PKs (FDR < 0.05; S3 Table). Additionally, CRP14 showed female-biased expression in soldiers (FDR < 0.01; S3 Table). Among all age classes in male reproductives, the expression of CRP1, CRP3, CRP5, CRP7, CRP11, and CRP14 peaked in mature PKs, and those of CRP9 and CRP10 peaked in young PKs (FDR < 0.05; S4 Table). CRP6, CRP12, and CRP13 showed the highest expression levels in alates (FDR < 0.05; S4 Table). In female reproductives, CRP6, CRP13, and CRP14 were expressed higher in female alates than young PQs, but CRP3 and CRP10 showed the opposite pattern (FDR < 0.01; S4 Table).

Among four annotated ferritins (Table 1 and S1 Table), ferritin 2 showed the highest expression in young PQs, and ferritin 3 and ferritin 4 showed in SQs (FDR < 0.05; S3 Fig and S2 Table). Ferritin 1 exhibited sexually dimorphic expression (FDR < 0.001, Table 1 and S5 Table), and it showed male-biased expression in soldiers (FDR < 0.01; S3 Table). In female reproductives, the expression of ferritin 3 and ferritin 4 increased with age (FDR < 0.05; S4 Table).

One melanotransferrin, one venom allergen, one termicin, and one thaumatin-like protein were also annotated (Table 1 and S1 Table). Melanotransferrin 1 and venom allergen 1 were more expressed in young reproductives and soldiers than other castes (FDR < 0.01; S3 Fig and S2 Table). The expression level of thaumatin-like protein 1 was low only in alates (FDR < 0.01; S2 Table). The expression levels of venom allergen 1 and thaumatin-like protein 1 differed between reproductive and neuter castes (reproductive status: FDR < 0.01, Table 1 and S5 Table). Comparing expression levels between sexes in each caste, venom allergen 1 showed female-biased expression in young reproductives and king-biased expression in mature reproductives (FDR < 0.05; S3 Table). Among age classes in male reproductives, the expression of melanotransferrin 1 peaked in young PKs (FDR < 0.01; S4 Table). In female reproductives, the expression levels of melanotransferrin 1 and venom allergen 1 increased with age (FDR < 0.05; S4 Table).

## Discussion

We found 197 immune-related genes (including 40 PRPs, 97 signalling proteins, 60 effectors) in *R*. *speratus* through a BLAST search querying amino acid sequences of these genes in various insect species (Table 1 and S1 Table). Of these 197 immune-related genes, 174 genes (88%) showed significant expression differences among castes (Table 1). In particular, there were a large number of genes showing soldier- or young reproductive-specific expression patterns. For example, 23% of CTLD-containing genes (6/26) and 20% of SP genes (14/70) had soldier-specific and young reproductive-specific expression patterns, respectively (FDR < 0.05; Figs 1 and 2, and S2 Table). While *Z*. *nevadensis* has 12 SPs [50], *R*. *speratus* has 70 candidates for SP (Fig 2, Table 1, and S1 Table). But the actual number of SPs in *R*. *speratus* needs to be further studied. Our results suggest that different immunoproteins are activated in different castes, leading to an immune division of labor in *R*. *speratus*.

Termicin is a termite-specific antifungal peptide and is present in hemocyte granules and salivary glands [21]. In *Pseudacanthotermes spiniger*, it inhibits the spores of fungi belonging to the genera *Nectria* and *Fusarium* and in *C*. *formosanus*, its expression increases following exposure to *M*. *anisopliae* [29]. On the other hand, transferrins are considered to participate in innate immune responses of termites through sequestration of iron away from iron-seeking pathogens, and in *M*. *darwiniensis*, the transferrin expression was increased after infection with *M*. *anisopliae* [36]. Moreover, *in R*. *chinensis*, the expressions of termicin and transferrin are increased after infection with *M*. *anisopliae* [7]. These facts indicate that termicin and transferrin are important immune proteins during fungal infections. Our study revealed that, in *R*. *speratus*, termicin 1 exhibited the highest expression in SQs, and transferrin 2 showed the highest expression in both mature PKs and SQs (S3 Fig and S2 and S3 Tables). Although the immune responses to infection with *M*. *anisopliae* have been studied in the damp wood termite *Z*. *angusticollis* [4,51], suggesting that female primary reproductives of this species exhibit trade-offs between reproduction and immunity [52], *Z*. *nevadensis* transcriptomic analysis showed that immune genes were expressed more highly in female reproductives than in other castes [50]. These imply that termite reproductives also have high resistance to fungal infections, which may be the factor enabling their extraordinary longevity.

Termite soldiers and workers are sometimes called “neuter” castes because they are sexually immature and sterile [53]. Therefore, sexually dimorphic gene expression in termite neuter castes has not attracted much attention. In our study, however, sexually dimorphic immune-related gene expression was detected in both reproductive and neuter castes (S5 Table). Comparing the gene expression levels between females and males in each caste, sexually dimorphic expression was detected not only in reproductive castes (6 genes in alates, 37 genes in young reproductives, and 43 genes in mature reproductives) but also in neuter castes (15 genes in soldiers and one gene in workers) (Table 1 and S3 Table). In soldiers, the genes showing both male- and female-biased expression were found; for example, two CTLs were higher in female soldiers than males (FDR < 0.01; Fig 1 and S3 Table), and two cathepsins showed the opposite pattern (FDR < 0.01; Fig 3 and S3 Table). In workers, only CTL3 showed a sexual difference, with higher expression in male workers than females (FDR < 0.05; Fig 1 and S3 Table). Because termite soldiers have been considered to have only one function, defending the colony against predators [54], it is noteworthy that there are soldier-specific immune-related genes and that some of them exhibit sexually dimorphic expression. Insofar as we know, this is the first study suggesting a sexual division of labor in termite neuter castes without phenotypic sexual dimorphism.

Each colony of *R*. *speratus* produces multitudes of alates in spring. After swarming, a pair of male and female alates establishes a new colony and starts producing offspring as a PK and PQ, respectively [55]. Just after establishing a new colony, the young PK and PQ must disinfect the inside of their nest and improve their immune systems, because they usually nest inside microbe-rich habitats such as rotten wood [30]. Therefore, we expected that expression of immune-related genes would increase when alates become young reproductives. Among the 197 immune-related genes, 162 genes (82%) showed age-dependent differential expression patterns in female and/or male reproductives (Table 1). In male reproductives, 71 genes showed the highest expression levels in young PKs, and 60 genes were highly expressed in mature PKs, while only 10 genes were more highly expressed in male alates than other age classes (FDR < 0.05; S4 Table). In female reproductives, 98 genes showed significantly higher expression levels in young PQs than female alates, while only 14 genes showed the opposite pattern (FDR < 0.05; S4 Table). Although our previous study revealed that the expression of many chemoreception-related genes in PKs and PQs increases after the colony is founded [40], this study indicated that a large number of immune-related genes also showed a similar tendency. In *R*. *speratus*, PQs live for at least 11 years [55], and PKs live much longer than PQs [56]. Increased expression of immune-related genes is likely an indispensible factor enabling the longevity of reproductives. Indeed, the recent study revealed that the SQs of *R*. *speratus* have higher activity of antioxidant enzymes (CAT and peroxiredoxin (Prx)) than workers and soldiers, which enables the lower oxidative damage to DNA, protein and lipid in SQs [57]. Because it is known that the individuals infected with *M*. *anisopliae* promote the activity of antioxidant enzymes such as CAT and SOD in *R*. *chinensis* [7], these enzymes might be related to both immunity and longevity in termite queens.

Lysozymes are antibacterial proteins against Gram-positive bacteria, and seven types of lysozymes have been found in various organisms: C-type lysozyme (in chickens), G-type lysozyme (in geese), I-type lysozyme (in invertebrates), P-type lysozyme (in nematodes), Plant-type lysozyme (in plants), bacteria-type lysozyme (in bacteria), and phage-type lysozyme (in bacteriophage T4) [27,58]. Insects have C-type, P-type, and I-type lysozymes [29,59], and some lepidopteran C-type lysozymes exhibit antibacterial activities even against Gram-negative bacteria [60]. In termites, the C-type lysozyme is used not only as an antibacterial agent but also as an egg recognition pheromone [32]. Previous studies revealed that, in *R*. *speratus*, two types of C-type lysozymes (Rs-Lys1 and Rs-Lys2) are synthesized in the salivary glands of workers and the ovaries of SQs, and both of them are used as egg recognition pheromones [32,48,49]. In this study, we identified eight types of lysozymes (C-type 1, C-type 2, C-type 3, P-type 1, I-type 1, I-type2, I-type 3, and C-type-like lysozyme), and one type of lysozyme-like protein (Fig 4, S4 Fig, Table 1, and S1 Table). Because phylogenetic analysis revealed that the amino acid sequence of C-type 1 was similar to that of Rs-Lys2 (S4 Fig), C-type 1 is presumed to function as the egg recognition pheromone in *R*. *speratus*. Our analyses also revealed that different types of lysozyme genes showed different expression patterns by caste: C-type 2, P-type, I-type 1, and I-type 3 were more highly expressed in soldiers than other castes; C-type 3 expression was higher in workers than other castes; and the C-type-like protein level was higher in young and mature reproductives and soldiers than other castes (Fig 4 and S2 Table). The functional significance of these caste-specific expression patterns remains unknown until the functional analysis of each lysozyme type can be clarified in future studies. In *D*. *melanogaster*, different types of lysozymes are expressed at different developmental stages [61]. Therefore, it is possible that the expression levels of each lysozyme type in *R*. *speratus* change based on caste roles and developmental stages.

To investigate the immune mechanism in *R*. *speratus*, we annotated the immune-related genes from a transcriptomic database constructed in our previous study [40]. Our transcriptomic analyses revealed that the activated immune genes clearly differed by caste, suggesting an immune division of labor in *R*. *speratus*. Some immune proteins also showed sexually dimorphic differences in their expression levels in the neuter castes, such as soldiers and workers, indicating a potential sexual division of labor in the anti-pathogenic defense system in *R*. *speratus*. Furthermore, many genes exhibited age-dependent expression differences across the PK and PQ life stages. These results will greatly contribute to a better understanding of the evolution of antimicrobial strategies, and the mechanism underlying longevity in termite reproductives.

## Supporting information

S1 Fig Differential expression of apolipophorinIIIs, SPIs, calpains, and LRPs among castes. The heatmap indicates the differential expression of three apolipophorinIIIs, seven SPIs, five calpains, and eight LRP transcripts among castes. Abbreviations are referred to in Fig 1. Relative expression level indicates the mean normalized Count per Million (CPM), ranging from black (scaled expression of 0) to white (scaled expression of 2.1). The tree at the left corresponds to hierarchical clustering of cluster-averaged expression. SPI: serine protease inhibitor, LRP: low-density lipoprotein receptor-related protein.
(EPS)

S2 Fig Differential expression of other signalling proteins among castes. The heatmap indicates the differential expression of two MHA and three 14-3-3 protein transcripts among castes. Abbreviations are referred to in Fig 1. Relative expression level indicates the mean normalized Count per Million (CPM), ranging from black (scaled expression of 0) to white (scaled expression of 1.6). The tree at the left corresponds to hierarchical clustering of cluster-averaged expression. MHA: minor histocompatibility protein.
(EPS)

S3 Fig Differential expression of other immune-related genes among castes. The heatmap indicates the differential expression of transcripts of 11 carboxypeptidases, 2 metacaspase-like cysteine peptidases, 14 cysteine-rich proteases, 4 ferritins, 3 transferrins, and 10 other effectors among castes. Abbreviations are referred to in Fig 1. Relative expression level indicates the mean normalized Count per Million (CPM), ranging from black (scaled expression of 0) to white (scaled expression of 2.5). The tree at the left corresponds to hierarchical clustering of cluster-averaged expression. AE-like CP: asparaginyl endopeptidase-like cysteine protease.
(EPS)

S4 Fig Molecular phylogenetic analysis of lysozyme sequences. The maximum likelihood trees based on the amino acid sequences of C- and P-type lysozymes (a) and those of I-type lysozymes (b) are shown. These trees include the lysozyme sequences of termites (*C*. *formosanus*, *R*. *speratus*, and *Z*. *nevadensis*) and the American cockroach (*Periplaneta americana*) obtained from the protein database UniProtKB (black letters, the Pfam accession numbers are shown in the parentheses) and our trascriptomic database (red letters, the DDBJ accession numbers are shown in the parentheses). Numbers at the each branch indicate bootstrap supports based on 1,000 replicates for maximum likelihood, maximum parsimony, and neighbor-joining methods, respectively. In the maximum likelihood method, the Whelan and Goldman model [62] was estimated as the best model based on BIC and AICc for both of the data set. Initial trees for the heuristic search were obtained by applying the neighbor-joining method to a matrix of pairwise distances estimated using a JTT model. Discrete gamma distributions were used to model evolutionary rate differences among sites (5 categories +G, 4.8178 for C- and P-type lysozymes, and 2.4288 for I-type lysozymes). The rate variation model allowed for some sites to be evolutionarily invariable ([+I], 13.2964% sites for C- and P-type lysozymes, and 14.9231% for I-type lysozymes). The tree is drawn to scale, with branch lengths measured in the number of substitutions per site. All positions containing gaps and missing data were eliminated after the alignments using the MUSCLE program with initial parameter values in MEGA6. These evolutionary analyses were conducted in MEGA6 [63]. A previous study reported that Rs-Lys1 (Q8IAD1) and Rs-Lys2 (Q8IAD0) are identical to Lys1 (A5H9H7) and Lys2 (A5H9H9), respectively [32].
(EPS)

S1 Table Summary results of BLAST homology searches. BLAST homology searches of amino acid sequences in *R*. *speratus* were performed on the sequences of 6 termites (*Z*. *nevadensis*, *C*. *secundus*, *C*. *formosanus*, *R*. *flavipes*, *R*. *chinensis*, *N*. *comatus*), 2 cockroaches (*P*. *americana*, *E*. *sinensis*), a beetle (*T*. *castaneum*), a honeybee (*A*. *mellifera*), a silk moth (*B*. *mori*) and a fruit fly (*D*. *melanogaster*).
(DOCX)

S2 Table Statistical results of differential expression among castes for each gene. Comparison of normalized counts per million (CPM) among castes (A: alates, Y: young primary kings (PKs) and queens, M: mature PKs and secondary queens (SQs), S: soldiers, W: workers) was conducted using edgeR package. Bold letters mean significant difference (FDR < 0.05). “Caste showing the highest expression” means the caste showing the largest mean CPM among all castes for each gene, and the largest mean CPM in the caste is more than 1.2 times as high as the mean CPM in the other castes. LR: likelihood ratio, FDR: false discovery rate, PRP: pattern recognition protein, S: Signalling protein, E: effector.
(DOCX)

S3 Table Statistical results of sexual difference of expression in each caste. Comparison of normalized counts per million (CPM) between sexes in each caste (PK: primary king, PQ: primary queens, SQ: secondary queen) was conducted using edgeR package. Bold letters mean significant difference (FDR < 0.05), and red and blue letters indicate the female- and male-biased expression, respectively. LR: likelihood ratio, FDR: false discovery rate, PRP: pattern recognition protein, S: Signalling protein, E: effector.
(DOCX)

S4 Table Statistical results of age-dependent expression changes in male or female reproductives. Comparison of normalized counts per million (CPM) among male reproductives (alates (A), young primary kings (Y) and mature primary kings (M)) or female ones (alates (A) and young primary queens (Y)) was conducted using edgeR package. Bold letters mean significant difference (FDR < 0.05). “Caste showing the highest expression” means the caste showing the largest mean CPM among all age classes for each gene, and the largest mean CPM in a certain age class is more than 1.2 times as high as the mean CPM in the other classes. LR: likelihood ratio, FDR: false discovery rate, PRP: pattern recognition protein, S: Signalling protein, E: effector.
(DOCX)

S5 Table Statistical results of differential expression between reproductive and neuter castes and between males and females. Comparison of normalized counts per million (CPM) between reproductive statuses (reproductive castes: alates, young PKs and PQs, and mature PKs and SQs; neuter castes: soldiers and workers) and between sexes was conducted by edgeR package. Bold letters mean significant differences (FDR < 0.05). LR: likelihood ratio, FDR: false discovery rate, PRP: pattern recognition protein, S: Signalling protein, E: effector.
(DOCX)
