# Supplementary material for: Caste-, sex-, and age-dependent expression of immune-related genes in a Japanese subterranean termite, Reticulitermes speratus
Source: PLoS One. 2017 Apr 14;12(4):e0175417. doi: 10.1371/journal.pone.0175417 (PMC5391962; doi:10.1371/journal.pone.0175417)
Supplement: S1 Table — BLAST homology searches of amino acid sequences in R. speratus were performed on the sequences of 6 termites (Z. nevadensis, C. secundus, C. formosanus, R. flavipes, R. chinensis, N. comatus), 2 cockroaches (P. americana, E. sinensis), a beetle (T. castaneum), a honeybee (A. mellifera), a silk moth (B. mori) and a fruit fly (D. melanogaster). (DOCX) [file pone.0175417.s005.docx]

**Table S1. Summary results of BLAST homology searches.**

| *Reticulitermes speratus* | |  |  |  | BLASTP queries | |  |  |  |  |
| --- | --- | --- | --- | --- | --- | --- | --- | --- | --- | --- |
| Functional category | Accession No. | Protein name | Contig |  | Accession No. | Protein name in species | Query species | E-Value | Identities | Positives |
| Pattern recognition protein | FX985231 | ApolipophorinIII 1 | comp767530_c1_seq2 |  | KDR17854.1 | Apolipophorin | *Zootermopsis nevadensis* | 0.00E+00 | 78% | 89% |
| Pattern recognition protein | FX985232 | ApolipophorinIII 2 isoform A | comp787860_c1_seq2 |  | EFA05722.1 | Apolipophorin-III | *Tribolium castaneum* | 4.00E-19 | 33% | 58% |
| Pattern recognition protein | FX985233 | ApolipophorinIII 2 isoform B | comp787860_c1_seq3 |  | ABY82793.1 | Apolipophorin-III like protein | *Apis mellifera* | 2.00E-12 | 27% | 49% |
| Pattern recognition protein | FX985234 | ApolipophorinIII 3 isoform A | comp801014_c18_seq1 |  | KDR17776.1 | Apolipophorin | *Zootermopsis nevadensis* | 0.00E+00 | 77% | 88% |
| Pattern recognition protein | FX985235 | ApolipophorinIII 3 isoform B | comp801014_c18_seq2 |  | KDR17777.1 | Apolipophorin | *Zootermopsis nevadensis* | 0.00E+00 | 76% | 86% |
| Pattern recognition protein | FX985236 | Brevican 1 isoform A | comp807283_c0_seq3 |  | EFA12925.2 | Brevican core protein-like Protein | *Tribolium castaneum* | 1.00E-107 | 62% | 74% |
| Pattern recognition protein | FX985237 | Brevican 1 isoform B | comp807283_c0_seq5 |  | KYB24590.1 | Brevican core protein-like Protein | *Tribolium castaneum* | 3.00E-92 | 83% | 93% |
| Pattern recognition protein | FX985238 | C-type lectin-like domain protein 1 | comp530380_c0_seq1 |  | KDR21331.1 | Hypothetical protein | *Zootermopsis nevadensis* | 8.00E-151 | 97% | 99% |
| Pattern recognition protein | FX985239 | C-type lectin-like domain protein 2 | comp750470_c3_seq4 |  | KDR10899.1 | Hypothetical protein | *Zootermopsis nevadensis* | 9.00E-118 | 85% | 93% |
| Pattern recognition protein | FX985240 | C-type lectin-like domain protein 3 | comp767535_c0_seq2 |  | AFZ78855.1 | C-type lectin | *Coptotermes formosanus* | 0.00E+00 | 77% | 83% |
| Pattern recognition protein | FX985241 | C-type lectin-like domain protein 4 isoform A | comp784003_c0_seq1 |  | AGM32432.1 | C-type lectin (CTL)/C-type lectin-like (CTLD) domain protein | *Coptotermes formosanus* | 5.00E-107 | 77% | 85% |
| Pattern recognition protein | FX985242 | C-type lectin-like domain protein 4 isoform B | comp784003_c0_seq2 |  | AGM32449.1 | Lectin-related protein | *Coptotermes formosanus* | 6.00E-45 | 87% | 93% |
| Pattern recognition protein | FX985243 | C-type lectin-like domain protein 5 isoform A | comp792791_c8_seq8 |  | ABI79325.1 | C-type lectin | *Bombyx mori* | 9.00E-112 | 70% | 79% |
| Pattern recognition protein | FX985244 | C-type lectin-like domain protein 5 isoform B | comp792791_c8_seq9 |  | KDR10900.1 | Hypothetical protein | *Zootermopsis nevadensis* | 5.00E-149 | 87% | 93% |
| Pattern recognition protein | FX985245 | C-type lectin-like domain protein 6 | comp792978_c1_seq2 |  | XP_003250964.1 | PREDICTED: Uncharacterized protein LOC100578121 isoform X1 | *Apis mellifera* | 2.00E-37 | 47% | 64% |
| Pattern recognition protein | FX985246 | C-type lectin-like domain protein 7 | comp794704_c0_seq1 |  | BAA18916.1 | Lectin-related protein | *Periplaneta americana* | 2.00E-59 | 47% | 61% |
| Pattern recognition protein | FX985247 | C-type lectin-like domain protein 8 isoform A | comp799434_c2_seq3 |  | XP_006558108.1 | PREDICTED: Uncharacterized protein LOC411888 isoform X2 | *Apis mellifera* | 0.00E+00 | 60% | 71% |
| Pattern recognition protein | FX985248 | C-type lectin-like domain protein 8 isoform B | comp799434_c2_seq4 |  | XP_015833551.1 | PREDICTED: Uncharacterized protein LOC660816 isoform X2 | *Tribolium castaneum* | 0.00E+00 | 69% | 79% |
| Pattern recognition protein | FX985249 | C-type lectin-like domain protein 8 isoform C | comp799434_c2_seq5 |  | AAF48429.2 | CG9095, isoform A | *Drosophila melanogaster* | 0.00E+00 | 67% | 77% |
| Pattern recognition protein | FX985250 | C-type lectin-like domain protein 8 isoform D | comp799434_c2_seq6 |  | EFA01290.2 | Hypothetical protein TcasGA2_TC002984 | *Tribolium castaneum* | 0.00E+00 | 70% | 79% |
| Pattern recognition protein | FX985251 | C-type lectin-like domain protein 9 | comp799616_c3_seq2 |  | KYB27694.1 | Hypothetical protein TcasGA2_TC031143 | *Tribolium castaneum* | 0.00E+00 | 48% | 64% |
| Pattern recognition protein | FX985252 | C-type lectin-like domain protein 10 isoform A | comp801028_c10_seq1 |  | EFA01299.2 | Hypothetical protein TcasGA2_TC003136 | *Tribolium castaneum* | 0.00E+00 | 57% | 71% |
| Pattern recognition protein | FX985253 | C-type lectin-like domain protein 10 isoform B | comp801028_c10_seq2 |  | EFA01298.2 | Hypothetical protein TcasGA2_TC003135 | *Tribolium castaneum* | 1.00E-123 | 43% | 59% |
| Pattern recognition protein | FX985254 | C-type lectin-like domain protein 11 | comp806056_c0_seq1 |  | KDR16268.1 | Hypothetical protein | *Zootermopsis nevadensis* | 7.00E-133 | 84% | 89% |
| Pattern recognition protein | FX985255 | C-type lectin-like domain protein 12 | comp808422_c0_seq2 |  | KDR21328.1 | Hypothetical protein | *Zootermopsis nevadensis* | 5.00E-161 | 92% | 97% |
| Pattern recognition protein | FX985256 | C-type lectin-like domain protein 13 | comp811281_c1_seq1 |  | EFA01446.2 | Hypothetical protein TcasGA2_TC030754 | *Tribolium castaneum* | 0.00E+00 | 72% | 83% |
| Pattern recognition protein | FX985257 | C-type lectin-like domain protein 14 isoform A | comp811593_c4_seq16 |  | ABN49272.1 | IP16828p | *Drosophila melanogaster* | 8.00E-161 | 68% | 82% |
| Pattern recognition protein | FX985258 | C-type lectin-like domain protein 14 isoform B | comp811593_c4_seq5 |  | XP_012544029.1 | PREDICTED: Uncharacterized protein LOC101740358 isoform X1 | *Bombyx mori* | 0.00E+00 | 76% | 88% |
| Pattern recognition protein | FX985259 | C-type lectin-like domain protein 15 | comp813460_c0_seq2 |  | ADB12587.1 | C-type lectin 10 | *Bombyx mori* | 1.00E-25 | 42% | 59% |
| Pattern recognition protein | FX985260 | C-type lectin-like domain protein 16 | comp814965_c8_seq3 |  | XP_001120871.2 | PREDICTED: Uncharacterized protein LOC724971 isoform X1 | *Apis mellifera* | 0.00E+00 | 51% | 66% |
| Pattern recognition protein | FX985261 | C-type lectin-like domain protein 17 isoform A | comp815003_c5_seq7 |  | KYB27838.1 | Hypothetical protein TcasGA2_TC007861 | *Tribolium castaneum* | 0.00E+00 | 59% | 74% |
| Pattern recognition protein | FX985262 | C-type lectin-like domain protein 17 isoform B | comp815003_c5_seq8 |  | EFA02868.2 | Hypothetical protein TcasGA2_TC007861 | *Tribolium castaneum* | 0.00E+00 | 59% | 74% |
| Pattern recognition protein & Effector | FX985263 | Gram-negative bacteria binding protein 1 | comp767461_c0_seq1 |  | ADJ19004.1 | Gram-negative bacteria binding protein 2 | *Reticulitermes flavipes* | 0.00E+00 | 96% | 98% |
| Pattern recognition protein & Effector | FX985264 | Gram-negative bacteria binding protein 2 isoform A | comp803088_c9_seq1 |  | EFA01356.1 | Gram-negative bacteria binding protein 3 | *Tribolium castaneum* | 4.00E-60 | 30% | 47% |
| Pattern recognition protein & Effector | FX985265 | Gram-negative bacteria binding protein 2 isoform B | comp803088_c9_seq4 |  | CAJ19054.1 | Gram-negative bacteria binding protein 2, isoform C | *Drosophila melanogaster* | 2.00E-17 | 34% | 52% |
| Pattern recognition protein & Effector | FX985266 | Gram-negative bacteria binding protein 3 | comp807368_c0_seq1 |  | AAZ08480.1 | Gram-negative bacteria binding protein 1 | *Nasutitermes comatus* | 0.00E+00 | 89% | 97% |
| Pattern recognition protein | FX985267 | Hemolymph lipopolysaccharide-binding protein 1 | comp753538_c0_seq1 |  | KDR10086.1 | Hemolymph lipopolysaccharide-binding protein | *Zootermopsis nevadensis* | 3.00E-84 | 53% | 69% |
| Pattern recognition protein | FX985268 | Hemolymph lipopolysaccharide-binding protein 2 isoform A | comp762974_c0_seq2 |  | KDR17640.1 | Hemolymph lipopolysaccharide-binding protein | *Zootermopsis nevadensis* | 1.00E-108 | 65% | 78% |
| Pattern recognition protein | FX985269 | Hemolymph lipopolysaccharide-binding protein 2 isoform B | comp762974_c0_seq4 |  | KDR17640.1 | Hemolymph lipopolysaccharide-binding protein | *Zootermopsis nevadensis* | 2.00E-108 | 65% | 78% |
| Pattern recognition protein | FX985270 | Hemolymph lipopolysaccharide-binding protein 3 isoform A | comp780783_c0_seq1 |  | KDR10085.1 | Hemolymph lipopolysaccharide-binding protein | *Zootermopsis nevadensis* | 2.00E-44 | 61% | 83% |
| Pattern recognition protein | FX985271 | Hemolymph lipopolysaccharide-binding protein 3 isoform B | comp780783_c0_seq2 |  | KDR10086.1 | Hemolymph lipopolysaccharide-binding protein | *Zootermopsis nevadensis* | 2.00E-60 | 45% | 61% |
| Pattern recognition protein | FX985272 | Hemolymph lipopolysaccharide-binding protein 4 | comp792351_c4_seq2 |  | KDR09967.1 | Hemolymph lipopolysaccharide-binding protein | *Zootermopsis nevadensis* | 1.00E-78 | 59% | 75% |
| Pattern recognition protein | FX985273 | Hemolymph lipopolysaccharide-binding protein 5 | comp799905_c2_seq5 |  | KDR12554.1 | Hemolymph lipopolysaccharide-binding protein | *Zootermopsis nevadensis* | 5.00E-94 | 57% | 75% |
| Pattern recognition protein | FX985274 | Hemolymph lipopolysaccharide-binding protein 6 | comp813460_c0_seq1 |  | BAA00616.1 | Lipopolysaccharide-binding protein | *Periplaneta americana* | 3.00E-72 | 46% | 65% |
| Pattern recognition protein | FX985275 | Laminin 1 | comp812882_c0_seq3 |  | KDR15871.1 | Laminin subunit beta-1 | *Zootermopsis nevadensis* | 0.00E+00 | 90% | 95% |
| Pattern recognition protein | FX985276 | Agglucetin 1 | comp806275_c7_seq3 |  | KDR22586.1 | Agglucetin subunit alpha-1 | *Zootermopsis nevadensis* | 0.00E+00 | 65% | 74% |
| Pattern recognition protein | FX985277 | endo-beta-1,4-glucanase 1 | comp784236_c0_seq1 |  | ACI45756.1 | Endo-beta-1,4-glucanase | *Coptotermes formosanus* | 0.00E+00 | 96% | 98% |
| Pattern recognition protein | FX985468 | Peptidoglycan recognition protein I-alpha | comp764160_c1_seq1 |  | KDR18605.1 | Peptidoglycan recognition protein I-alpha, partial | *Zootermopsis nevadensis* | 2.00E-91 | 75% | 87% |
| Pattern recognition protein | FX985469 | Peptidoglycan recognition protein LB | comp774827_c0_seq1 |  | KDR07311.1 | Peptidoglycan-recognition protein LB | *Zootermopsis nevadensis* | 2.00E-102 | 69% | 80% |
| Pattern recognition protein | FX985470 | Peptidoglycan recognition protein LE 1 | comp780102_c3_seq1 |  | KDR12888.1 | Peptidoglycan-recognition protein LE, partial | *Zootermopsis nevadensis* | 5.00E-120 | 52% | 63% |
| Pattern recognition protein | FX985471 | Peptidoglycan recognition protein LE 2 | comp780102_c3_seq2 |  | EFA01280.1 | Peptidoglycan-recognition protein LE-like Protein | *Tribolium castaneum* | 8.00E-73 | 58% | 70% |
| Pattern recognition protein | FX985472 | Peptidoglycan recognition protein SC 1 | comp790513_c3_seq1 |  | KDR07939.1 | Peptidoglycan-recognition protein SC2, partial | *Zootermopsis nevadensis* | 2.00E-83 | 66% | 81% |
| Pattern recognition protein | FX985473 | Peptidoglycan recognition protein SC 2 | comp799630_c9_seq6 |  | XP_008192540.1 | PREDICTED: peptidoglycan-recognition protein SC1a/b-like | *Tribolium castaneum* | 9.00E-38 | 41% | 59% |
| Pattern recognition protein | FX985474 | Peptidoglycan recognition protein SD | comp799630_c7_seq7 |  | CAD89189.1 | peptidoglycan recognition protein SD | *Drosophila melanogaster* | 4.00E-43 | 44% | 61% |
| Pattern recognition protein | FX985475 | Peptidoglycan recognition protein | comp808145_c8_seq1 |  | ADG48753.1 | CG11709, partial | *Drosophila melanogaster* | 2.00E-23 | 41% | 55% |
| Signalling protein | FX985278 | Serine protease 1 | comp427959_c1_seq1 |  | KYB25980.1 | Serine protease 53-like Protein | *Tribolium castaneum* | 1.00E-23 | 32% | 52% |
| Signalling protein | FX985279 | Serine protease 2 | comp724924_c0_seq1 |  | KDR11537.1 | Tryptase-2 | *Zootermopsis nevadensis* | 4.00E-116 | 62% | 74% |
| Signalling protein | FX985280 | Serine protease 3 | comp733028_c0_seq1 |  | KDR09772.1 | Plasma kallikrein | *Zootermopsis nevadensis* | 0.00E+00 | 75% | 84% |
| Signalling protein | FX985281 | Serine protease 4 | comp738530_c3_seq2 |  | EEZ99215.1 | Serine protease P13 | *Tribolium castaneum* | 2.00E-152 | 76% | 86% |
| Signalling protein | FX985282 | Serine protease 5 | comp743779_c0_seq2 |  | AGM32955.1 | Clip domain serine protease | *Coptotermes formosanus* | 1.00E-92 | 84% | 91% |
| Signalling protein | FX985283 | Serine protease 6 isoform A | comp765941_c0_seq1 |  | KDR09767.1 | Coagulation factor XI | *Zootermopsis nevadensis* | 1.00E-78 | 74% | 88% |
| Signalling protein | FX985284 | Serine protease 6 isoform B | comp765941_c0_seq5 |  | KDR08703.1 | Transmembrane protease, serine 3 | *Zootermopsis nevadensis* | 4.00E-65 | 79% | 89% |
| Signalling protein | FX985285 | Serine protease 7 | comp767439_c0_seq1 |  | EFA05743.2 | Serine protease H164 | *Tribolium castaneum* | 8.00E-58 | 41% | 55% |
| Signalling protein | FX985286 | Serine protease 8 | comp774015_c1_seq2 |  | EFA04595.2 | Serine protease P140 | *Tribolium castaneum* | 9.00E-115 | 52% | 64% |
| Signalling protein | FX985287 | Serine protease 9 | comp779063_c0_seq3 |  | KDR21045.1 | Leukocyte elastase inhibitor | *Zootermopsis nevadensis* | 0.00E+00 | 60% | 75% |
| Signalling protein | FX985288 | Serine protease 10 | comp781134_c0_seq2 |  | AAX33734.1 | MPA3 allergen | *Periplaneta americana* | 2.00E-132 | 73% | 82% |
| Signalling protein | FX985289 | Serine protease 11 | comp783460_c16_seq1 |  | NP_650366.1 | HTRA2-related serine protease, isoform A | *Drosophila melanogaster* | 7.00E-136 | 57% | 75% |
| Signalling protein | FX985290 | Serine protease 12 | comp783898_c0_seq2 |  | KDR14147.1 | Leukocyte elastase inhibitor C | *Zootermopsis nevadensis* | 1.00E-177 | 65% | 79% |
| Signalling protein | FX985291 | Serine protease 13 | comp784494_c0_seq1 |  | EFA05748.1 | Serine protease P166 | *Tribolium castaneum* | 2.00E-63 | 46% | 62% |
| Signalling protein | FX985292 | Serine protease 14 | comp784971_c3_seq1 |  | KDR09405.1 | Heparin cofactor 2 | *Zootermopsis nevadensis* | 1.00E-124 | 52% | 65% |
| Signalling protein | FX985293 | Serine protease 15 | comp785613_c5_seq1 |  | KDR18614.1 | Kallikrein-14 | *Zootermopsis nevadensis* | 1.00E-37 | 70% | 76% |
| Signalling protein | FX985294 | Serine protease 16 | comp785613_c9_seq3 |  | EFA01278.1 | Serine protease P41 | *Tribolium castaneum* | 3.00E-58 | 44% | 60% |
| Signalling protein | FX985295 | Serine protease 17 | comp785667_c3_seq1 |  | KDR12496.1 | Urokinase-type plasminogen activator | *Zootermopsis nevadensis* | 0.00E+00 | 84% | 91% |
| Signalling protein | FX985296 | Serine protease 18 | comp785691_c13_seq36 |  | EFA11567.1 | Serine protease P53 | *Tribolium castaneum* | 1.00E-178 | 53% | 62% |
| Signalling protein | FX985297 | Serine protease 19 | comp786206_c13_seq3 |  | KDR21723.1 | Putative serine protease K12H4.7 | *Zootermopsis nevadensis* | 0.00E+00 | 80% | 88% |
| Signalling protein | FX985298 | Serine protease 20 | comp788457_c0_seq1 |  | AEI58606.1 | Serine protease | *Eupolyphaga sinensis* | 7.00E-20 | 31% | 49% |
| Signalling protein | FX985299 | Serine protease 21 | comp790351_c6_seq1 |  | EFA01268.1 | Serine protease P36 | *Tribolium castaneum* | 3.00E-127 | 90% | 95% |
| Signalling protein | FX985300 | Serine protease 22 | comp790351_c7_seq1 |  | EEZ97707.2 | Serine protease P126 | *Tribolium castaneum* | 3.00E-36 | 37% | 52% |
| Signalling protein | FX985301 | Serine protease 23 | comp790434_c2_seq2 |  | AFZ78860.1 | Serine protease | *Coptotermes formosanus* | 2.00E-65 | 44% | 60% |
| Signalling protein | FX985302 | Serine protease 24 | comp791050_c7_seq2 |  | EFA11582.2 | Serine protease P100 | *Tribolium castaneum* | 3.00E-109 | 69% | 83% |
| Signalling protein | FX985303 | Serine protease 25 isoform A | comp791125_c0_seq1 |  | AFZ78858.1 | Serine protease | *Coptotermes formosanus* | 6.00E-149 | 79% | 88% |
| Signalling protein | FX985304 | Serine protease 25 isoform B | comp791125_c0_seq4 |  | EFA02920.2 | Serine protease H81 | *Tribolium castaneum* | 3.00E-24 | 32% | 49% |
| Signalling protein | FX985305 | Serine protease 26 | comp792736_c1_seq1 |  | BAD93199.1 | Serine protease | *Bombyx mori* | 1.00E-120 | 50% | 67% |
| Signalling protein | FX985306 | Serine protease 27 | comp793628_c0_seq1 |  | KDR09407.1 | Serpin B10 | *Zootermopsis nevadensis* | 0.00E+00 | 64% | 78% |
| Signalling protein | FX985307 | Serine protease 28 | comp794399_c16_seq1 |  | KDR09404.1 | Serpin I2 | *Zootermopsis nevadensis* | 0.00E+00 | 80% | 90% |
| Signalling protein | FX985308 | Serine protease 29 | comp797769_c3_seq2 |  | EFA07413.2 | Serine protease P84 | *Tribolium castaneum* | 3.00E-143 | 79% | 87% |
| Signalling protein | FX985309 | Serine protease 30 | comp798682_c7_seq1 |  | EFA09211.1 | Serine protease P69 | *Tribolium castaneum* | 7.00E-80 | 47% | 64% |
| Signalling protein | FX985310 | Serine protease 31 | comp798864_c1_seq5 |  | KDR19769.1 | Leukocyte elastase inhibitor C | *Zootermopsis nevadensis* | 0.00E+00 | 67% | 80% |
| Signalling protein | FX985311 | Serine protease 32 | comp799311_c9_seq59 |  | KYB29568.1 | Serine protease P54 | *Tribolium castaneum* | 0.00E+00 | 66% | 80% |
| Signalling protein | FX985312 | Serine protease 33 | comp799714_c4_seq1 |  | EFA11600.2 | Serine protease P144 | *Tribolium castaneum* | 0.00E+00 | 63% | 73% |
| Signalling protein | FX985313 | Serine protease 34 | comp799894_c0_seq3 |  | CAO78846.1 | Serine protease | *Drosophila melanogaster* | 2.00E-33 | 35% | 52% |
| Signalling protein | FX985314 | Serine protease 35 isoform A | comp800118_c0_seq2 |  | EFA05688.1 | Serine protease P154 | *Tribolium castaneum* | 1.00E-43 | 37% | 53% |
| Signalling protein | FX985315 | Serine protease 35 isoform B | comp800118_c0_seq3 |  | AAB02551.1 | Serine protease 3 | *Drosophila melanogaster* | 1.00E-16 | 60% | 70% |
| Signalling protein | FX985316 | Serine protease 35 isoform C | comp800118_c0_seq4 |  | EFA05665.2 | Serine protease P150 | *Tribolium castaneum* | 2.00E-44 | 39% | 54% |
| Signalling protein | FX985317 | Serine protease 36 | comp800120_c1_seq1 |  | KDR09408.1 | Serpin B8 | *Zootermopsis nevadensis* | 0.00E+00 | 66% | 79% |
| Signalling protein | FX985318 | Serine protease 37 | comp800600_c12_seq1 |  | KDR16030.1 | Antithrombin-III | *Zootermopsis nevadensis* | 0.00E+00 | 61% | 75% |
| Signalling protein | FX985319 | Serine protease 38 | comp800653_c7_seq2 |  | EFA10739.2 | Serine protease H129 | *Tribolium castaneum* | 0.00E+00 | 60% | 76% |
| Signalling protein | FX985320 | Serine protease 39 | comp801386_c4_seq6 |  | KDR21309.1 | Reversion-inducing cysteine-rich protein with Kazal motifs, partial | *Zootermopsis nevadensis* | 0.00E+00 | 88% | 94% |
| Signalling protein | FX985321 | Serine protease 40 | comp801909_c6_seq9 |  | EEZ99180.1 | Serine protease H1 | *Tribolium castaneum* | 8.00E-68 | 37% | 53% |
| Signalling protein | FX985322 | Serine protease 41 | comp802111_c1_seq4 |  | EFA04569.1 | Serine protease P133 | *Tribolium castaneum* | 1.00E-92 | 44% | 60% |
| Signalling protein | FX985323 | Serine protease 42 | comp802318_c10_seq2 |  | EFA11559.1 | Serine protease P44 | *Tribolium castaneum* | 7.00E-93 | 50% | 65% |
| Signalling protein | FX985324 | Serine protease 43 isoform A | comp802758_c4_seq18 |  | EEZ99266.1 | Serine protease P22 | *Tribolium castaneum* | 1.00E-111 | 57% | 71% |
| Signalling protein | FX985325 | Serine protease 43 isoform B | comp802758_c4_seq5 |  | KYB29219.1 | Serine protease P12 | *Tribolium castaneum* | 3.00E-142 | 71% | 80% |
| Signalling protein | FX985326 | Serine protease 44 | comp803169_c10_seq1 |  | EFA04547.1 | Serine protease H110 | *Tribolium castaneum* | 6.00E-52 | 40% | 57% |
| Signalling protein | FX985327 | Serine protease 45 | comp803169_c2_seq1 |  | EFA04604.1 | Serine protease H143 | *Tribolium castaneum* | 3.00E-48 | 38% | 54% |
| Signalling protein | FX985328 | Serine protease 46 isoform A | comp803252_c1_seq24 |  | KYB24610.1 | Serine protease P43 | *Tribolium castaneum* | 1.00E-84 | 53% | 69% |
| Signalling protein | FX985329 | Serine protease 46 isoform B | comp803252_c1_seq7 |  | EFA12222.2 | Serine protease P60 | *Tribolium castaneum* | 4.00E-72 | 49% | 66% |
| Signalling protein | FX985330 | Serine protease 47 | comp803292_c1_seq9 |  | KDR16770.1 | Antithrombin-III | *Zootermopsis nevadensis* | 0.00E+00 | 81% | 90% |
| Signalling protein | FX985331 | Serine protease 48 | comp803673_c4_seq8 |  | EFA07506.2 | Serine protease P96 | *Tribolium castaneum* | 3.00E-82 | 45% | 62% |
| Signalling protein | FX985332 | Serine protease 49 | comp804136_c1_seq1 |  | AEI58574.1 | Serine protease | *Eupolyphaga sinensis* | 4.00E-104 | 69% | 81% |
| Signalling protein | FX985333 | Serine protease 50 | comp805337_c8_seq1 |  | EFA11565.2 | Serine protease H51 | *Tribolium castaneum* | 0.00E+00 | 62% | 71% |
| Signalling protein | FX985334 | Serine protease 51 | comp806481_c0_seq1 |  | EFA01296.2 | Serine protease H42 | *Tribolium castaneum* | 7.00E-102 | 53% | 71% |
| Signalling protein | FX985335 | Serine protease 52 | comp806560_c1_seq1 |  | XP_015838954.1 | PREDICTED: serine protease 27 | *Tribolium castaneum* | 4.00E-48 | 40% | 59% |
| Signalling protein | FX985336 | Serine protease 53 | comp807436_c5_seq1 |  | KDR14765.1 | Leukocyte elastase inhibitor B | *Zootermopsis nevadensis* | 6.00E-175 | 57% | 67% |
| Signalling protein | FX985337 | Serine protease 54 | comp808029_c1_seq3 |  | AGM32953.1 | Serine protease | *Coptotermes formosanus* | 2.00E-50 | 45% | 59% |
| Signalling protein | FX985338 | Serine protease 55 | comp808071_c0_seq4 |  | KYB27427.1 | Serine protease P134 | *Tribolium castaneum* | 7.00E-21 | 43% | 60% |
| Signalling protein | FX985339 | Serine protease 56 | comp808072_c3_seq2 |  | KDR23303.1 | Transmembrane protease, serine 2 | *Zootermopsis nevadensis* | 0.00E+00 | 92% | 97% |
| Signalling protein | FX985340 | Serine protease 57 | comp809908_c2_seq15 |  | KDR21743.1 | Leukocyte elastase inhibitor C | *Zootermopsis nevadensis* | 0.00E+00 | 67% | 81% |
| Signalling protein | FX985341 | Serine protease 58 | comp810319_c1_seq3 |  | KDR10950.1 | Serpin B9 | *Zootermopsis nevadensis* | 0.00E+00 | 84% | 93% |
| Signalling protein | FX985342 | Serine protease 59 isoform A | comp810786_c0_seq2 |  | EFA07451.1 | Serine protease P90 | *Tribolium castaneum* | 1.00E-75 | 40% | 57% |
| Signalling protein | FX985343 | Serine protease 59 isoform B | comp810786_c0_seq3 |  | EFA07452.2 | Serine protease P91 | *Tribolium castaneum* | 8.00E-97 | 42% | 60% |
| Signalling protein | FX985344 | Serine protease 60 | comp810972_c7_seq45 |  | KYB29465.1 | Serine protease H57 | *Tribolium castaneum* | 1.00E-45 | 38% | 50% |
| Signalling protein | FX985345 | Serine protease 61 | comp811350_c1_seq5 |  | KDR16748.1 | Serpin B8 | *Zootermopsis nevadensis* | 0.00E+00 | 82% | 89% |
| Signalling protein | FX985346 | Serine protease 62 | comp811884_c0_seq1 |  | EEZ99245.2 | Serine protease P19 | *Tribolium castaneum* | 2.00E-103 | 57% | 74% |
| Signalling protein | FX985347 | Serine protease 63 isoform A | comp812342_c0_seq124 |  | KDR19733.1 | Limulus clotting factor C | *Zootermopsis nevadensis* | 6.00E-77 | 85% | 93% |
| Signalling protein | FX985348 | Serine protease 63 isoform B | comp812342_c0_seq85 |  | EEZ99332.2 | Serine protease P46 | *Tribolium castaneum* | 2.00E-91 | 36% | 52% |
| Signalling protein | FX985349 | Serine protease 64 isoform A | comp812604_c0_seq1 |  | EFA05735.1 | Serine protease P160 | *Tribolium castaneum* | 2.00E-46 | 38% | 56% |
| Signalling protein | FX985350 | Serine protease 64 isoform B | comp812604_c0_seq2 |  | EFA10764.1 | Serine protease H132 | *Tribolium castaneum* | 3.00E-48 | 38% | 54% |
| Signalling protein | FX985351 | Serine protease 64 isoform C | comp812604_c0_seq4 |  | AAB02552.1 | Serine protease 1 | *Drosophila melanogaster* | 7.00E-50 | 42% | 57% |
| Signalling protein | FX985352 | Serine protease 65 isoform A | comp812612_c8_seq24 |  | EEZ99182.2 | Serine protease H3 | *Tribolium castaneum* | 6.00E-112 | 50% | 65% |
| Signalling protein | FX985353 | Serine protease 65 isoform B | comp812612_c8_seq30 |  | EEZ99181.1 | Serine protease H2 | *Tribolium castaneum* | 4.00E-178 | 61% | 75% |
| Signalling protein | FX985354 | Serine protease 66 | comp812747_c1_seq2 |  | EFA07557.2 | Serine protease P135 | *Tribolium castaneum* | 3.00E-105 | 52% | 73% |
| Signalling protein | FX985355 | Serine protease 67 | comp814760_c1_seq5 |  | EEZ99238.2 | Serine protease H18 | *Tribolium castaneum* | 2.00E-23 | 33% | 52% |
| Signalling protein | FX985356 | Serine protease 68 | comp814965_c8_seq4 |  | EFA05685.1 | Serine protease P153 | *Tribolium castaneum* | 0.00E+00 | 48% | 64% |
| Signalling protein | FX985357 | Serine protease 69 isoform A | comp815432_c0_seq58 |  | KYB27716.1 | Serine protease P146 | *Tribolium castaneum* | 0.00E+00 | 50% | 68% |
| Signalling protein | FX985358 | Serine protease 69 isoform B | comp815432_c0_seq7 |  | EFA04636.2 | Serine protease P146 | *Tribolium castaneum* | 0.00E+00 | 50% | 68% |
| Signalling protein | FX985359 | Serine protease 70 | comp815964_c1_seq3 |  | EFA09207.2 | Serine protease P66 | *Tribolium castaneum* | 1.00E-70 | 40% | 58% |
| Signalling protein | FX985360 | Kazal-type serine protease inhibitor domain-containing protein 1 | comp807785_c2_seq1 |  | KDR13208.1 | Kazal-type serine protease inhibitor domain-containing protein 1 | *Zootermopsis nevadensis* | 1.00E-154 | 80% | 88% |
| Signalling protein | FX985361 | Serine protease inhibitor 1 isoform A | comp798864_c1_seq5 |  | XP_012547756.1 | PREDICTED: Serine protease inhibitor 2 isoform X1 | *Bombyx mori* | 4.00E-69 | 38% | 62% |
| Signalling protein | FX985362 | Serine protease inhibitor 1 isoform B | comp798864_c1_seq6 |  | XP_012547717.1 | PREDICTED: Serine protease inhibitor 21 isoform X1 | *Bombyx mori* | 9.00E-69 | 36% | 61% |
| Signalling protein | FX985363 | Serine protease inhibitor 1 isoform C | comp798864_c1_seq9 |  | CAB63101.1 | Serine protease inhibitor (serpin-6) | *Drosophila melanogaster* | 5.00E-68 | 37% | 59% |
| Signalling protein | FX985364 | Serine protease inhibitor 2 | comp803311_c2_seq1 |  | XP_975339.3 | PREDICTED: Serine protease inhibitor dipetalogastin | *Tribolium castaneum* | 0.00E+00 | 72% | 82% |
| Signalling protein | FX985365 | Serine protease inhibitor 3 | comp807436_c5_seq2 |  | XP_012552764.1 | PREDICTED: Serine protease inhibitor 34 isoform X1 | *Bombyx mori* | 1.00E-47 | 36% | 50% |
| Signalling protein | FX985366 | Serine protease inhibitor 4 | comp812612_c8_seq29 |  | XP_016769184.1 | PREDICTED: Serine protease 44, isoform X1 | *Apis mellifera* | 5.00E-156 | 56% | 70% |
| Signalling protein | FX985367 | Serine protease inhibitor dipetalogastin 1 | comp801625_c9_seq1 |  | KDR09778.1 | Serine protease inhibitor dipetalogastin | *Zootermopsis nevadensis* | 6.00E-83 | 69% | 84% |
| Signalling protein | FX985368 | Serine protease inhibitor dipetalogastin 2 | comp805888_c14_seq4 |  | XP_004924430.1 | PREDICTED: Serine protease inhibitor dipetalogastin | *Bombyx mori* | 1.00E-11 | 31% | 40% |
| Signalling protein | FX985369 | Prophenoloxidase activating factor 1 | comp802704_c6_seq2 |  | AAL31707.1 | Prophenoloxidase activating factor 3 | *Bombyx mori* | 1.00E-80 | 38% | 55% |
| Signalling protein | FX985370 | 14-3-3 protein 1 | comp482595_c0_seq1 |  | XP_006559884.1 | 14-3-3 protein epsilon | *Apis mellifera* | 0.00E+00 | 96% | 98% |
| Signalling protein | FX985371 | 14-3-3 protein 2 | comp786340_c2_seq8 |  | XP_006566160.1 | 14-3-3 protein zeta, isoform X1 | *Apis mellifera* | 3.00E-179 | 98% | 99% |
| Signalling protein | FX985372 | 14-3-3 protein 3 | comp799140_c0_seq3 |  | AFZ78827.1 | 14-3-3 protein | *Coptotermes formosanus* | 4.00E-60 | 39% | 68% |
| Signalling protein | FX985373 | Calpain 1 | comp782312_c3_seq1 |  | KDR21632.1 | Calpain-1 catalytic subunit | *Zootermopsis nevadensis* | 0.00E+00 | 96% | 98% |
| Signalling protein | FX985374 | Calpain 2 isoform A | comp788807_c1_seq11 |  | XP_015833877.1 | PREDICTED: Calpain-B isoform X8 | *Tribolium castaneum* | 0.00E+00 | 71% | 84% |
| Signalling protein | FX985375 | Calpain 2 isoform B | comp788807_c1_seq20 |  | KDR10636.1 | Calpain-A | *Zootermopsis nevadensis* | 0.00E+00 | 93% | 96% |
| Signalling protein | FX985376 | Calpain 2 isoform C | comp788807_c1_seq22 |  | XP_015833876.1 | PREDICTED: Calpain-B isoform X7 | *Tribolium castaneum* | 0.00E+00 | 65% | 78% |
| Signalling protein | FX985377 | Calpain 2 isoform D | comp788807_c1_seq27 |  | XP_004923127.1 | PREDICTED: Calpain-B isoform X3 | *Bombyx mori* | 0.00E+00 | 70% | 82% |
| Signalling protein | FX985378 | Calpain 2 isoform E | comp788807_c1_seq4 |  | NP_001153877.1 | Calpain-B | *Apis mellifera* | 0.00E+00 | 76% | 88% |
| Signalling protein | FX985379 | Calpain 2 isoform F | comp788807_c1_seq7 |  | XP_015833872.1 | PREDICTED: Calpain-A isoform X3 | *Tribolium castaneum* | 0.00E+00 | 64% | 77% |
| Signalling protein | FX985380 | Calpain 2 isoform G | comp788807_c1_seq8 |  | XP_012552936.1 | PREDICTED: Calpain-A isoform X2 | *Bombyx mori* | 0.00E+00 | 63% | 75% |
| Signalling protein | FX985381 | Calpain 3 | comp796517_c4_seq6 |  | KDR22954.1 | Calpain-C | *Zootermopsis nevadensis* | 0.00E+00 | 98% | 99% |
| Signalling protein | FX985382 | Calpain 4 | comp798743_c3_seq2 |  | P27398.2 | Calpain-D | *Drosophila melanogaster* | 0.00E+00 | 73% | 82% |
| Signalling protein | FX985383 | Calpain 5 | comp805847_c0_seq9 |  | KDR07957.1 | Calpain-7 | *Zootermopsis nevadensis* | 0.00E+00 | 86% | 93% |
| Signalling protein | FX985384 | Minor histocompatibility antigen 1 | comp787208_c1_seq1 |  | KDR15355.1 | CD109 antigen | *Zootermopsis nevadensis* | 0.00E+00 | 87% | 93% |
| Signalling protein | FX985385 | Minor histocompatibility antigen 2 | comp802510_c1_seq2 |  | KDR15612.1 | Minor histocompatibility protein HA-1 | *Zootermopsis nevadensis* | 0.00E+00 | 77% | 85% |
| Signalling protein | FX985386 | Low-density lipoprotein receptor-related protein 1 | comp775319_c0_seq7 |  | KDR16389.1 | Low-density lipoprotein receptor-related protein 12 | *Zootermopsis nevadensis* | 0.00E+00 | 78% | 86% |
| Signalling protein | FX985387 | Low-density lipoprotein receptor-related protein 2 | comp785049_c4_seq1 |  | KDR24262.1 | Low-density lipoprotein receptor-related protein 4 | *Zootermopsis nevadensis* | 0.00E+00 | 87% | 92% |
| Signalling protein | FX985388 | Low-density lipoprotein receptor-related protein 3 | comp792997_c0_seq4 |  | KDR15023.1 | Low-density lipoprotein receptor-related protein | *Zootermopsis nevadensis* | 0.00E+00 | 72% | 81% |
| Signalling protein | FX985389 | Low-density lipoprotein receptor-related protein 4 | comp798761_c7_seq4 |  | KDR10188.1 | Low-density lipoprotein receptor class A domain-containing protein 3 | *Zootermopsis nevadensis* | 0.00E+00 | 56% | 72% |
| Signalling protein | FX985390 | Low-density lipoprotein receptor-related protein 5 | comp802210_c11_seq1 |  | KDR08547.1 | Low-density lipoprotein receptor-related protein 6 | *Zootermopsis nevadensis* | 0.00E+00 | 89% | 93% |
| Signalling protein | FX985391 | Low-density lipoprotein receptor-related protein 6 | comp804712_c10_seq1 |  | KDR15449.1 | Low-density lipoprotein receptor-related protein | *Zootermopsis nevadensis* | 0.00E+00 | 69% | 78% |
| Signalling protein | FX985392 | Low-density lipoprotein receptor-related protein 7 | comp807965_c0_seq3 |  | KDR21560.1 | Low-density lipoprotein receptor-related protein 4 | *Zootermopsis nevadensis* | 0.00E+00 | 70% | 80% |
| Signalling protein | FX985393 | Low-density lipoprotein receptor-related protein 8 | comp813705_c1_seq6 |  | KDR12649.1 | Low-density lipoprotein receptor-related protein 2 | *Zootermopsis nevadensis* | 0.00E+00 | 92% | 97% |
| Signalling protein | FX985394 | Four and a half LIM domains protein 1 isoform A | comp792188_c9_seq1 |  | XP_006566079.1 | PREDICTED: four and a half LIM domains protein 2 isoform X6 | *Apis mellifera* | 0.00E+00 | 77% | 85% |
| Signalling protein | FX985395 | Four and a half LIM domains protein 1 isoform B | comp792188_c9_seq2 |  | XP_976021.1 | PREDICTED: four and a half LIM domains protein 2 isoform X3 | *Tribolium castaneum* | 0.00E+00 | 69% | 80% |
| Signalling protein | FX985396 | Four and a half LIM domains protein 1 isoform C | comp792188_c9_seq3 |  | XP_004924797.1 | PREDICTED: four and a half LIM domains protein 2 isoform X5 | *Bombyx mori* | 4.00E-150 | 82% | 89% |
| Signalling protein | FX985397 | Four and a half LIM domains protein 1 isoform D | comp792188_c9_seq4 |  | XP_006566084.1 | PREDICTED: four and a half LIM domains protein 2 isoform X8 | *Apis mellifera* | 0.00E+00 | 93% | 96% |
| Effector | FX985398 | Carboxypeptidase 1 | comp516001_c0_seq1 |  | KDR22871.1 | Zinc carboxypeptidase A1 | *Zootermopsis nevadensis* | 0.00E+00 | 78% | 90% |
| Effector | FX985399 | Carboxypeptidase 2 | comp751477_c0_seq2 |  | KDR10344.1 | Carboxypeptidase E | *Zootermopsis nevadensis* | 0.00E+00 | 79% | 90% |
| Effector | FX985400 | Carboxypeptidase 3 | comp777264_c0_seq1 |  | AFZ78840.1 | Carboxypeptidase | *Coptotermes formosanus* | 0.00E+00 | 84% | 89% |
| Effector | FX985401 | Carboxypeptidase 4 | comp782324_c0_seq8 |  | KDR22873.1 | Zinc carboxypeptidase | *Zootermopsis nevadensis* | 0.00E+00 | 79% | 87% |
| Effector | FX985402 | Carboxypeptidase 5 | comp793589_c2_seq32 |  | KDR18068.1 | Carboxypeptidase M | *Zootermopsis nevadensis* | 0.00E+00 | 88% | 92% |
| Effector | FX985403 | Carboxypeptidase 6 | comp800691_c0_seq1 |  | AGM32338.1 | Serine carboxypeptidase | *Coptotermes formosanus* | 0.00E+00 | 86% | 92% |
| Effector | FX985404 | Carboxypeptidase 7 | comp801424_c0_seq8 |  | KDR15626.1 | Putative carboxypeptidase PM20D1 | *Zootermopsis nevadensis* | 0.00E+00 | 92% | 96% |
| Effector | FX985405 | Carboxypeptidase 8 | comp806499_c4_seq2 |  | KDR22449.1 | Carboxypeptidase D | *Zootermopsis nevadensis* | 0.00E+00 | 80% | 88% |
| Effector | FX985406 | Carboxypeptidase 9 | comp806673_c7_seq1 |  | KDR21622.1 | Carboxypeptidase B | *Zootermopsis nevadensis* | 0.00E+00 | 79% | 88% |
| Effector | FX985407 | Carboxypeptidase 10 | comp810469_c5_seq59 |  | KDR21431.1 | Carboxypeptidase M | *Zootermopsis nevadensis* | 1.00E-158 | 80% | 89% |
| Effector | FX985408 | Carboxypeptidase 11 | comp815218_c7_seq8 |  | KDR09452.1 | Putative serine carboxypeptidase CPVL | *Zootermopsis nevadensis* | 0.00E+00 | 80% | 89% |
| Effector | FX985409 | Cathepsin 1 | comp501672_c1_seq1 |  | EFA06474.1 | Cathepsin L-like Protein | *Tribolium castaneum* | 5.00E-16 | 37% | 63% |
| Effector | FX985410 | Cathepsin 2 | comp723285_c0_seq1 |  | ABJ97193.1 | Cathepsin L-like proteinase | *Bombyx mori* | 3.00E-62 | 35% | 57% |
| Effector | FX985411 | Cathepsin 3 | comp730638_c0_seq1 |  | XP_016773595.1 | PREDICTED: Cathepsin L1 | *Apis mellifera* | 4.00E-66 | 40% | 62% |
| Effector | FX985412 | Cathepsin 4 | comp773773_c0_seq1 |  | AAP50847.1 | Cathepsin D | *Bombyx mori* | 0.00E+00 | 69% | 81% |
| Effector | FX985413 | Cathepsin 5 | comp789120_c4_seq1 |  | AFZ78846.1 | Cathepsin O-like protein | *Coptotermes formosanus* | 4.00E-120 | 91% | 95% |
| Effector | FX985414 | Cathepsin 6 | comp790271_c0_seq1 |  | BAG70408.1 | Cathepsin L like protein | *Bombyx mori* | 3.00E-88 | 46% | 65% |
| Effector | FX985415 | Cathepsin 7 | comp801909_c6_seq4 |  | AGM32335.1 | Cathepsin L-like protein | *Coptotermes formosanus* | 0.00E+00 | 95% | 97% |
| Effector | FX985416 | Cathepsin 8 | comp807693_c2_seq1 |  | KDR17524.1 | Putative cysteine proteinase | *Zootermopsis nevadensis* | 0.00E+00 | 73% | 84% |
| Effector | FX985417 | Cathepsin 9 | comp807931_c0_seq2 |  | KDR18937.1 | Cathepsin B | *Zootermopsis nevadensis* | 0.00E+00 | 78% | 85% |
| Effector | FX985418 | Cathepsin 10 | comp808549_c0_seq1 |  | AGM32452.1 | Cathepsin D-like aspartic peptidase | *Coptotermes formosanus* | 2.00E-96 | 65% | 78% |
| Effector | FX985419 | Lysozyme C type 1 | comp729118_c0_seq4 |  | AFZ78838.1 | C-type lysozyme-3 | *Coptotermes formosanus* | 3.00E-80 | 72% | 83% |
| Effector | FX985420 | Putative lysozyme-like protein | comp757104_c1_seq1 |  | ACV32411.1 | Lysozyme | *Tribolium castaneum* | 1.00E-30 | 61% | 74% |
| Effector | FX985421 | Lysozyme P type | comp767477_c0_seq1 |  | AGA16574.1 | P-type lysozyme | *Coptotermes formosanus* | 4.00E-82 | 86% | 91% |
| Effector | FX985422 | Lysozyme C type 2 | comp787754_c0_seq1 |  | AFZ78837.1 | C-type lysozyme-2 | *Coptotermes formosanus* | 3.00E-83 | 88% | 92% |
| Effector | FX985423 | Putative lysozyme C type-like protein | comp794904_c6_seq2 |  | KDR16709.1 | Lysozyme | *Zootermopsis nevadensis* | 3.00E-41 | 48% | 69% |
| Effector | FX985424 | Lysozyme I type 1 | comp800275_c1_seq1 |  | AFZ78836.1 | I-type lysozyme | *Coptotermes formosanus* | 4.00E-98 | 87% | 93% |
| Effector | FX985425 | Lysozyme I type 2 | comp802015_c1_seq10 |  | KDR20129.1 | Lysozyme 1 | *Zootermopsis nevadensis* | 1.00E-69 | 75% | 85% |
| Effector | FX985426 | Lysozyme I type 3 | comp808068_c10_seq1 |  | KDR15949.1 | Lysozyme 3 | *Zootermopsis nevadensis* | 1.00E-70 | 70% | 81% |
| Effector | FX985427 | Lysozyme C-type 3 isoform A | comp801401_c3_seq1 |  | AFI81524.1 | C-3 lysozyme | *Periplaneta americana* | 6.00E-33 | 48% | 62% |
| Effector | FX985428 | Lysozyme C-type 3 isoform B | comp801401_c3_seq2 |  | AFI81524.1 | C-3 lysozyme | *Periplaneta americana* | 2.00E-31 | 47% | 64% |
| Effector | FX985429 | Lysozyme C-type 3 isoform C, partial | comp801401_c3_seq3 |  | AFI81524.1 | C-3 lysozyme | *Periplaneta americana* | 1.00E-21 | 47% | 60% |
| Effector | FX985430 | Metacaspase-like cysteine peptidase 1 | comp726130_c0_seq1 |  | AFZ78842.1 | Metacaspase-like cysteine peptidase | *Coptotermes formosanus* | 3.00E-69 | 47% | 62% |
| Effector | FX985431 | Metacaspase-like cysteine peptidase 2 | comp738300_c0_seq1 |  | AGM32866.1 | Metacaspase-like cysteine peptidase | *Coptotermes formosanus* | 8.00E-47 | 37% | 54% |
| Effector | FX985432 | Asparaginyl endopeptidase-like cysteine peptidase 1 | comp760145_c0_seq2 |  | AFZ78841.1 | Asparaginyl endopeptidase-like cysteine peptidase | *Coptotermes formosanus* | 6.00E-179 | 64% | 76% |
| Effector | FX985433 | Lysosomal Pro-X carboxypeptidase 1 | comp803357_c2_seq3 |  | KDR15687.1 | Lysosomal Pro-X carboxypeptidase | *Zootermopsis nevadensis* | 0.00E+00 | 81% | 90% |
| Effector | FX985434 | Prolixicin antimicrobial protein 1 | comp792034_c5_seq4 |  | AFZ78839.1 | Prolixicin antimicrobial protein | *Coptotermes formosanus* | 3.00E-43 | 77% | 82% |
| Effector | FX985455 | Transferrin 1 | comp803710_c5_seq2 |  | XP_004927448.1 | Transferrin-like | *Bombyx mori* | 5.00E-99 | 33% | 47% |
| Effector | FX985456 | Transferrin 2 | comp805153_c4_seq8 |  | ABN05623.1 | Putative transferrin, partial | *Cryptotermes secundus* | 3.00E-58 | 77% | 93% |
| Effector | FX985457 | Transferrin 3 | comp810803_c12_seq1 |  | KDR19727.1 | Transferrin | *Zootermopsis nevadensis* | 0.00E+00 | 77% | 87% |
| Effector | FX985460 | Termicin 1 | comp719353_c0_seq1 |  | ACO36902.1 | Termicin | *Reticulitermes chinensis* | 2.00E-27 | 95% | 98% |
| Effector | FX985435 | Cysteine-rich protein 1 | comp719554_c0_seq1 |  | NP_651509.2 | Secreted protein, acidic, cysteine-rich, isoform A | *Drosophila melanogaster* | 3.00E-89 | 62% | 77% |
| Effector | FX985436 | Cysteine-rich protein 2 | comp755858_c0_seq1 |  | XP_004923112.1 | PREDICTED: Cysteine-rich PDZ-binding protein | *Bombyx mori* | 4.00E-13 | 40% | 57% |
| Effector | FX985437 | Cysteine-rich protein 3 | comp793630_c3_seq9 |  | XP_971778.1 | PREDICTED: Cysteine-rich with EGF-like domain protein 2 | *Tribolium castaneum* | 1.00E-159 | 61% | 78% |
| Effector | FX985438 | Cysteine-rich protein 4 isoform A | comp804172_c0_seq5 |  | XP_006565634.1 | Cysteine-rich DPF motif domain-containing protein 1 | *Apis mellifera* | 4.00E-27 | 44% | 72% |
| Effector | FX985439 | Cysteine-rich protein 4 isoform B | comp804172_c0_seq7 |  | XP_008195524.1 | PREDICTED: Cysteine-rich DPF motif domain-containing protein 1 | *Tribolium castaneum* | 1.00E-28 | 41% | 56% |
| Effector | FX985440 | Cysteine-rich protein 5 | comp806874_c10_seq3 |  | XP_006566358.1 | PREDICTED: Run domain Beclin-1-interacting and cysteine-rich domain-containing protein | *Apis mellifera* | 0.00E+00 | 64% | 78% |
| Effector | FX985441 | Cysteine-rich protein 6 | comp807330_c16_seq8 |  | XP_012550772.1 | PREDICTED: SH3 and cysteine-rich domain-containing protein 3-like | *Bombyx mori* | 5.00E-96 | 82% | 89% |
| Effector | FX985442 | Cysteine-rich protein 7 isoform A | comp809087_c9_seq2 |  | XP_001123220.2 | PREDICTED: Cysteine-rich protein 2-binding protein-like | *Apis mellifera* | 2.00E-115 | 58% | 73% |
| Effector | FX985443 | Cysteine-rich protein 7 isoform B | comp809087_c9_seq3 |  | KYB26599.1 | Cysteine-rich protein 2-binding protein-like Protein | *Tribolium castaneum* | 2.00E-57 | 57% | 69% |
| Effector | FX985444 | Cysteine-rich protein 8 | comp809203_c1_seq2 |  | EFA01871.1 | Glutaredoxin domain-containing cysteine-rich protein CG31559-like Protein | *Tribolium castaneum* | 1.00E-116 | 65% | 79% |
| Effector | FX985445 | Cysteine-rich protein 9 | comp781856_c14_seq1 |  | KDR16505.1 | Cysteine-rich hydrophobic domain 2 protein | *Zootermopsis nevadensis* | 5.00E-118 | 97% | 99% |
| Effector | FX985446 | Cysteine-rich protein 10 | comp782293_c0_seq1 |  | AFZ78847.1 | Putative cysteine-rich protein 1 | *Coptotermes formosanus* | 6.00E-52 | 99% | 99% |
| Effector | FX985447 | Cysteine-rich protein 11 | comp787140_c12_seq1 |  | KDR14990.1 | Cysteine-rich motor neuron 1 protein | *Zootermopsis nevadensis* | 0.00E+00 | 77% | 85% |
| Effector | FX985448 | Cysteine-rich protein 12 | comp794809_c8_seq1 |  | KDR16237.1 | Cysteine-rich secretory protein 2 | *Zootermopsis nevadensis* | 8.00E-180 | 85% | 93% |
| Effector | FX985449 | Cysteine-rich protein 13 | comp807330_c16_seq63 |  | KDR24515.1 | SH3 and cysteine-rich domain-containing protein 2 | *Zootermopsis nevadensis* | 2.00E-127 | 81% | 86% |
| Effector | FX985450 | Cysteine-rich protein 14 | comp815871_c20_seq7 |  | KDR23766.1 | Basement membrane-specific heparan sulfate proteoglycan core protein | *Zootermopsis nevadensis* | 0.00E+00 | 87% | 93% |
| Effector | FX985451 | Ferritin 1 | comp3273972_c0_seq1 |  | XP_967819.1 | PREDICTED: Soma ferritin | *Tribolium castaneum* | 3.00E-33 | 37% | 57% |
| Effector | FX985452 | Ferritin 2 | comp718037_c3_seq1 |  | XP_006568941.1 | PREDICTED: Soma ferritin | *Apis mellifera* | 4.00E-72 | 62% | 77% |
| Effector | FX985453 | Ferritin 3 | comp718766_c0_seq1 |  | EEZ98069.1 | Ferritin subunit-like Protein | *Tribolium castaneum* | 2.00E-83 | 58% | 75% |
| Effector | FX985454 | Ferritin 4 | comp751102_c0_seq4 |  | EEZ98578.1 | Ferritin light chain-like Protein | *Tribolium castaneum* | 2.00E-69 | 52% | 68% |
| Effector | FX985458 | Melanotransferrin 1 | comp781515_c14_seq2 |  | KDR17893.1 | Melanotransferrin | *Zootermopsis nevadensis* | 0.00E+00 | 88% | 94% |
| Effector | FX985459 | Venom allergen 1 | comp796033_c0_seq1 |  | KDR14384.1 | Venom allergen 3 | *Zootermopsis nevadensis* | 6.00E-109 | 62% | 77% |
| Effector | FX985461 | Thaumatin-like protein 1 | comp807921_c1_seq2 |  | AGA16575.1 | Thaumatin-like protein 1 | *Coptotermes formosanus* | 4.00E-139 | 81% | 89% |

BLAST homology searches of amino acid sequences in *R. speratus* were performed on the sequences of 6 termites (*Z. nevadensis*, *C. secundus*, *C. formosanus*, *R. flavipes*, *R. chinensis*, *N. comatus*), 2 cockroaches (*P. americana*, *E. sinensis*), a beetle (*T. castaneum*), a honeybee (*A. mellifera*), a silk moth (*B. mori*) and a fruit fly (*D. melanogaster*).
