# Supplementary material for: Caste-, sex-, and age-dependent expression of immune-related genes in a Japanese subterranean termite, Reticulitermes speratus
Source: PLoS One. 2017 Apr 14;12(4):e0175417. doi: 10.1371/journal.pone.0175417 (PMC5391962; doi:10.1371/journal.pone.0175417)
Supplement: S3 Table — Comparison of normalized counts per million (CPM) between sexes in each caste (PK: primary king, PQ: primary queens, SQ: secondary queen) was conducted using edgeR package. Bold letters mean significant difference (FDR < 0.05), and red and blue letters indicate the female- and male-biased expression, respectively. LR: likelihood ratio, FDR: false discovery rate, PRP: pattern recognition protein, S: Signalling protein, E: effector. (DOCX) [file pone.0175417.s007.docx]

**Table S3. Statistical results of sexual difference of expression in each caste.**

| Functional category | Gene name | Male & Female Alate | | |  | Young PK & PQ | | |  | Mature PK vs SQ | | |  | Male & Female Soldier | | |  | Male & Female Worker | | |
| --- | --- | --- | --- | --- | --- | --- | --- | --- | --- | --- | --- | --- | --- | --- | --- | --- | --- | --- | --- | --- |
|  |  | LR | P-value | FDR |  | LR | P-value | FDR |  | LR | P-value | FDR |  | LR | P-value | FDR |  | LR | P-value | FDR |
| PRP | ApolipophorinIII 1 | 3.35 | 0.07 | 1.00 |  | 2.67 | 0.10 | 0.68 |  | 9.35 | 0.00 | 0.21 |  | 0.58 | 0.45 | 1.00 |  | 0.56 | 0.46 | 1.00 |
| PRP | ApolipophorinIII 2 | 2.34 | 0.13 | 1.00 |  | 0.09 | 0.77 | 1.00 |  | 11.39 | 0.00 | 0.09 |  | 0.00 | 0.96 | 1.00 |  | 0.13 | 0.71 | 1.00 |
| PRP | ApolipophorinIII 3 | 9.41 | 0.00 | 0.50 |  | 0.24 | 0.63 | 1.00 |  | **39.67** | **0.00** | **0.00** |  | 1.30 | 0.25 | 1.00 |  | 1.69 | 0.19 | 1.00 |
| PRP | Brevican 1 | 0.12 | 0.73 | 1.00 |  | **29.94** | **0.00** | **0.00** |  | 0.37 | 0.54 | 1.00 |  | 3.94 | 0.05 | 1.00 |  | 0.51 | 0.47 | 1.00 |
| PRP | C-type lectin-like domain protein 1 | 2.33 | 0.13 | 1.00 |  | 8.57 | 0.00 | 0.05 |  | 1.69 | 0.19 | 1.00 |  | 3.99 | 0.05 | 1.00 |  | 1.51 | 0.22 | 1.00 |
| PRP | C-type lectin-like domain protein 2 | 1.93 | 0.17 | 1.00 |  | 5.94 | 0.01 | 0.17 |  | 10.79 | 0.00 | 0.12 |  | 0.48 | 0.49 | 1.00 |  | 0.79 | 0.38 | 1.00 |
| PRP | C-type lectin-like domain protein 3 | 0.32 | 0.57 | 1.00 |  | 3.76 | 0.05 | 0.44 |  | 0.54 | 0.46 | 1.00 |  | 1.30 | 0.25 | 1.00 |  | **18.28** | **0.00** | **0.04** |
| PRP | C-type lectin-like domain protein 4 | 0.71 | 0.40 | 1.00 |  | **17.22** | **0.00** | **0.00** |  | 12.04 | 0.00 | 0.07 |  | 1.30 | 0.26 | 1.00 |  | 0.18 | 0.67 | 1.00 |
| PRP | C-type lectin-like domain protein 5 | 0.49 | 0.49 | 1.00 |  | 0.08 | 0.78 | 1.00 |  | 1.12 | 0.29 | 1.00 |  | 0.33 | 0.57 | 1.00 |  | 0.10 | 0.75 | 1.00 |
| PRP | C-type lectin-like domain protein 6 | 2.49 | 0.11 | 1.00 |  | 0.70 | 0.40 | 1.00 |  | **53.79** | **0.00** | **0.00** |  | 0.18 | 0.67 | 1.00 |  | 0.10 | 0.76 | 1.00 |
| PRP | C-type lectin-like domain protein 7 | 0.38 | 0.54 | 1.00 |  | **21.72** | **0.00** | **0.00** |  | 1.06 | 0.30 | 1.00 |  | 0.03 | 0.85 | 1.00 |  | 4.03 | 0.04 | 1.00 |
| PRP | C-type lectin-like domain protein 8 | 0.02 | 0.88 | 1.00 |  | 0.00 | 0.98 | 1.00 |  | 2.05 | 0.15 | 1.00 |  | 0.75 | 0.39 | 1.00 |  | 0.32 | 0.57 | 1.00 |
| PRP | C-type lectin-like domain protein 9 | 5.48 | 0.02 | 1.00 |  | **29.59** | **0.00** | **0.00** |  | **26.53** | **0.00** | **0.00** |  | 0.16 | 0.69 | 1.00 |  | 0.01 | 0.94 | 1.00 |
| PRP | C-type lectin-like domain protein 10 | 2.49 | 0.11 | 1.00 |  | **16.50** | **0.00** | **0.00** |  | 0.64 | 0.42 | 1.00 |  | **47.89** | **0.00** | **0.00** |  | 1.60 | 0.21 | 1.00 |
| PRP | C-type lectin-like domain protein 11 | 0.94 | 0.33 | 1.00 |  | 1.29 | 0.26 | 0.85 |  | 2.95 | 0.09 | 1.00 |  | 0.36 | 0.55 | 1.00 |  | 16.30 | 0.00 | 0.09 |
| PRP | C-type lectin-like domain protein 12 | 1.37 | 0.24 | 1.00 |  | 1.65 | 0.20 | 0.85 |  | 0.07 | 0.80 | 1.00 |  | 0.46 | 0.50 | 1.00 |  | 0.55 | 0.46 | 1.00 |
| PRP | C-type lectin-like domain protein 13 | 0.61 | 0.44 | 1.00 |  | 0.01 | 0.94 | 1.00 |  | 2.65 | 0.10 | 1.00 |  | 0.37 | 0.54 | 1.00 |  | 0.23 | 0.63 | 1.00 |
| PRP | C-type lectin-like domain protein 14 | 1.17 | 0.28 | 1.00 |  | 1.45 | 0.23 | 0.85 |  | 1.71 | 0.19 | 1.00 |  | **28.67** | **0.00** | **0.00** |  | 0.16 | 0.69 | 1.00 |
| PRP | C-type lectin-like domain protein 15 | 9.06 | 0.00 | 0.54 |  | 0.10 | 0.75 | 1.00 |  | 1.12 | 0.29 | 1.00 |  | 0.06 | 0.81 | 1.00 |  | 1.87 | 0.17 | 1.00 |
| PRP | C-type lectin-like domain protein 16 | 0.23 | 0.63 | 1.00 |  | 0.00 | 0.96 | 1.00 |  | 1.63 | 0.20 | 1.00 |  | 0.27 | 0.60 | 1.00 |  | 0.61 | 0.44 | 1.00 |
| PRP | C-type lectin-like domain protein 17 | 12.20 | 0.00 | 0.19 |  | **96.93** | **0.00** | **0.00** |  | **14.95** | **0.00** | **0.02** |  | 0.37 | 0.54 | 1.00 |  | 0.36 | 0.55 | 1.00 |
| PRP & E | Gram-negative binding protein 1 | 1.74 | 0.19 | 1.00 |  | 5.51 | 0.02 | 0.21 |  | 2.67 | 0.10 | 1.00 |  | 1.63 | 0.20 | 1.00 |  | 2.38 | 0.12 | 1.00 |
| PRP & E | Gram-negative binding protein 2 | 1.58 | 0.21 | 1.00 |  | **35.91** | **0.00** | **0.00** |  | 3.30 | 0.07 | 1.00 |  | 0.97 | 0.32 | 1.00 |  | 0.17 | 0.68 | 1.00 |
| PRP & E | Gram-negative binding protein 3 | 6.82 | 0.01 | 1.00 |  | 1.63 | 0.20 | 0.85 |  | 3.72 | 0.05 | 1.00 |  | 1.99 | 0.16 | 1.00 |  | 0.15 | 0.70 | 1.00 |
| PRP | Hemolymph lipopolysaccharide-binding protein 1 | 1.48 | 0.22 | 1.00 |  | 0.17 | 0.68 | 1.00 |  | 5.07 | 0.02 | 1.00 |  | 1.80 | 0.18 | 1.00 |  | 0.07 | 0.79 | 1.00 |
| PRP | Hemolymph lipopolysaccharide-binding protein 2 | 1.91 | 0.17 | 1.00 |  | 0.21 | 0.65 | 1.00 |  | **27.30** | **0.00** | **0.00** |  | 0.02 | 0.88 | 1.00 |  | 1.00 | 0.32 | 1.00 |
| PRP | Hemolymph lipopolysaccharide-binding protein 3 | 0.15 | 0.70 | 1.00 |  | 0.02 | 0.90 | 1.00 |  | 1.47 | 0.22 | 1.00 |  | 1.10 | 0.29 | 1.00 |  | 8.62 | 0.00 | 0.86 |
| PRP | Hemolymph lipopolysaccharide-binding protein 4 | 0.04 | 0.84 | 1.00 |  | 0.75 | 0.39 | 1.00 |  | 1.27 | 0.26 | 1.00 |  | 3.01 | 0.08 | 1.00 |  | 0.35 | 0.55 | 1.00 |
| PRP | Hemolymph lipopolysaccharide-binding protein 5 | 7.27 | 0.01 | 0.93 |  | 0.93 | 0.34 | 0.98 |  | 0.19 | 0.66 | 1.00 |  | 1.08 | 0.30 | 1.00 |  | 3.58 | 0.06 | 1.00 |
| PRP | Hemolymph lipopolysaccharide-binding protein 6 | 9.06 | 0.00 | 0.54 |  | 0.10 | 0.75 | 1.00 |  | 1.12 | 0.29 | 1.00 |  | 0.06 | 0.81 | 1.00 |  | 1.87 | 0.17 | 1.00 |
| PRP | Laminin 1 | 3.52 | 0.06 | 1.00 |  | **19.06** | **0.00** | **0.00** |  | 2.45 | 0.12 | 1.00 |  | 3.55 | 0.06 | 1.00 |  | 0.44 | 0.51 | 1.00 |
| PRP | Agglucetin 1 | 4.08 | 0.04 | 1.00 |  | 3.35 | 0.07 | 0.52 |  | 0.05 | 0.82 | 1.00 |  | 0.38 | 0.54 | 1.00 |  | 0.25 | 0.61 | 1.00 |
| PRP | Endo-beta-1,4-glucanase 1 | 1.36 | 0.24 | 1.00 |  | **30.85** | **0.00** | **0.00** |  | **106.13** | **0.00** | **0.00** |  | 0.03 | 0.87 | 1.00 |  | 0.07 | 0.80 | 1.00 |
| PRP | Peptidoglycan recognition protein I-alpha | 0.02 | 0.90 | 1.00 |  | 3.50 | 0.06 | 0.50 |  | 8.74 | 0.00 | 0.28 |  | 4.71 | 0.03 | 1.00 |  | 1.27 | 0.26 | 1.00 |
| PRP | Peptidoglycan recognition protein LB | 0.01 | 0.94 | 1.00 |  | 0.85 | 0.36 | 1.00 |  | 0.39 | 0.53 | 1.00 |  | 2.16 | 0.14 | 1.00 |  | 1.94 | 0.16 | 1.00 |
| PRP | Peptidoglycan recognition protein LE 1 | 11.90 | 0.00 | 0.21 |  | 5.95 | 0.01 | 0.17 |  | 8.39 | 0.00 | 0.32 |  | 1.02 | 0.31 | 1.00 |  | 1.88 | 0.17 | 1.00 |
| PRP | Peptidoglycan recognition protein SC 1 | 0.54 | 0.49 | 1.00 |  | 2.36 | 0.12 | 0.73 |  | **29.3** | **0.00** | **0.00** |  | 4.67 | 0.03 | 1.00 |  | 0.66 | 0.42 | 1.00 |
| PRP | Peptidoglycan recognition protein SC 2 | 0.47 | 0.49 | 1.00 |  | 8.68 | 0.00 | 0.05 |  | 11.5 | 0.00 | 0.08 |  | 0.34 | 0.56 | 1.00 |  | 0.02 | 0.90 | 1.00 |
| PRP | Peptidoglycan recognition protein SD | 1.90 | 0.17 | 1.00 |  | 0.20 | 0.65 | 1.00 |  | 4.51 | 0.03 | 1.00 |  | 0.20 | 0.66 | 1.00 |  | 0.61 | 0.43 | 1.00 |
| PRP | Peptidoglycan recognition protein | 3.07 | 0.08 | 1.00 |  | 0.79 | 0.37 | 1.00 |  | 0.22 | 0.64 | 1.00 |  | 0.20 | 0.65 | 1.00 |  | 2.15 | 0.14 | 1.00 |
| S | Serine protease 1 | 0.00 | 1.00 | 1.00 |  | 0.00 | 1.00 | 1.00 |  | 0.00 | 1.00 | 1.00 |  | **45.92** | **0.00** | **0.00** |  | 0.00 | 1.00 | 1.00 |
| S | Serine protease 2 | 0.51 | 0.47 | 1.00 |  | 3.35 | 0.07 | 0.52 |  | **26.45** | **0.00** | **0.00** |  | 6.72 | 0.01 | 0.84 |  | 2.49 | 0.11 | 1.00 |
| S | Serine protease 3 | 8.77 | 0.00 | 0.59 |  | 3.50 | 0.06 | 0.50 |  | 10.34 | 0.00 | 0.14 |  | **55.83** | **0.00** | **0.00** |  | 0.00 | 0.96 | 1.00 |
| S | Serine protease 4 | 12.39 | 0.00 | 0.18 |  | **25.46** | **0.00** | **0.00** |  | **96.52** | **0.00** | **0.00** |  | 1.83 | 0.18 | 1.00 |  | 4.82 | 0.03 | 1.00 |
| S | Serine protease 5 | 0.06 | 0.81 | 1.00 |  | 0.00 | 0.98 | 1.00 |  | 1.94 | 0.16 | 1.00 |  | 1.18 | 0.28 | 1.00 |  | 0.04 | 0.85 | 1.00 |
| S | Serine protease 6 | 2.72 | 0.10 | 1.00 |  | 2.09 | 0.15 | 0.80 |  | 0.41 | 0.52 | 1.00 |  | 1.96 | 0.16 | 1.00 |  | 0.05 | 0.82 | 1.00 |
| S | Serine protease 7 | 0.00 | 0.97 | 1.00 |  | 0.07 | 0.79 | 1.00 |  | 0.09 | 0.77 | 1.00 |  | 0.81 | 0.37 | 1.00 |  | 0.51 | 0.47 | 1.00 |
| S | Serine protease 8 | 1.33 | 0.25 | 1.00 |  | 1.24 | 0.27 | 0.85 |  | 2.60 | 0.11 | 1.00 |  | 1.96 | 0.16 | 1.00 |  | 3.01 | 0.08 | 1.00 |
| S | Serine protease 9 | 0.42 | 0.52 | 1.00 |  | 1.98 | 0.16 | 0.84 |  | 0.75 | 0.39 | 1.00 |  | 1.35 | 0.25 | 1.00 |  | 4.28 | 0.04 | 1.00 |
| S | Serine protease 10 | 0.93 | 0.34 | 1.00 |  | 8.54 | 0.00 | 0.05 |  | 7.29 | 0.01 | 0.50 |  | **22.16** | **0.00** | **0.00** |  | 0.29 | 0.59 | 1.00 |
| S | Serine protease 11 | 0.92 | 0.34 | 1.00 |  | **15.29** | **0.00** | **0.00** |  | 12.59 | 0.00 | 0.05 |  | 3.19 | 0.07 | 1.00 |  | 5.64 | 0.02 | 1.00 |
| S | Serine protease 12 | 0.30 | 0.58 | 1.00 |  | 4.00 | 0.05 | 0.41 |  | 0.76 | 0.38 | 1.00 |  | 0.34 | 0.56 | 1.00 |  | 5.30 | 0.02 | 1.00 |
| S | Serine protease 13 | 3.92 | 0.05 | 1.00 |  | 3.85 | 0.05 | 0.43 |  | 5.11 | 0.02 | 1.00 |  | 12.49 | 0.00 | 0.11 |  | 0.92 | 0.34 | 1.00 |
| S | Serine protease 14 | 11.90 | 0.00 | 0.21 |  | 0.83 | 0.36 | 1.00 |  | **24.19** | **0.00** | **0.00** |  | 5.88 | 0.02 | 1.00 |  | 0.99 | 0.32 | 1.00 |
| S | Serine protease 15 | 0.20 | 0.65 | 1.00 |  | 1.01 | 0.31 | 0.92 |  | 5.57 | 0.02 | 1.00 |  | 0.00 | 0.95 | 1.00 |  | 1.53 | 0.22 | 1.00 |
| S | Serine protease 16 | 0.36 | 0.55 | 1.00 |  | 0.44 | 0.51 | 1.00 |  | 5.47 | 0.02 | 1.00 |  | 0.10 | 0.75 | 1.00 |  | 3.01 | 0.08 | 1.00 |
| S | Serine protease 17 | 10.08 | 0.00 | 0.40 |  | 0.33 | 0.57 | 1.00 |  | 4.27 | 0.04 | 1.00 |  | 6.03 | 0.01 | 1.00 |  | 1.08 | 0.30 | 1.00 |
| S | Serine protease 18 | **22.95** | **0.00** | **0.00** |  | **120.90** | **0.00** | **0.00** |  | **39.84** | **0.00** | **0.00** |  | 0.11 | 0.74 | 1.00 |  | 0.78 | 0.38 | 1.00 |
| S | Serine protease 19 | 0.04 | 0.83 | 1.00 |  | 0.00 | 0.95 | 1.00 |  | 3.75 | 0.05 | 1.00 |  | 0.61 | 0.44 | 1.00 |  | 0.24 | 0.62 | 1.00 |
| S | Serine protease 20 | 0.08 | 0.77 | 1.00 |  | 0.09 | 0.77 | 1.00 |  | **311.16** | **0.00** | **0.00** |  | 1.02 | 0.31 | 1.00 |  | 0.06 | 0.81 | 1.00 |
| S | Serine protease 21 | 1.20 | 0.27 | 1.00 |  | 0.00 | 0.98 | 1.00 |  | 3.33 | 0.07 | 1.00 |  | 0.54 | 0.46 | 1.00 |  | 9.29 | 0.00 | 0.75 |
| S | Serine protease 22 | 2.04 | 0.15 | 1.00 |  | 0.09 | 0.76 | 1.00 |  | 2.27 | 0.13 | 1.00 |  | 0.18 | 0.67 | 1.00 |  | 0.17 | 0.68 | 1.00 |
| S | Serine protease 23 | 1.78 | 0.18 | 1.00 |  | 5.11 | 0.02 | 0.24 |  | 7.69 | 0.01 | 0.44 |  | 1.58 | 0.21 | 1.00 |  | 0.25 | 0.62 | 1.00 |
| S | Serine protease 24 | 0.00 | 1.00 | 1.00 |  | 1.17 | 0.28 | 0.85 |  | 1.68 | 0.20 | 1.00 |  | 0.00 | 1.00 | 1.00 |  | 1.35 | 0.25 | 1.00 |
| S | Serine protease 25 | 14.28 | 0.00 | 0.09 |  | 0.00 | 1.00 | 1.00 |  | 1.02 | 0.31 | 1.00 |  | 0.58 | 0.45 | 1.00 |  | 1.77 | 0.18 | 1.00 |
| S | Serine protease 26 | 1.79 | 0.18 | 1.00 |  | 7.46 | 0.01 | 0.08 |  | 0.01 | 0.93 | 1.00 |  | 6.18 | 0.01 | 0.97 |  | 1.03 | 0.31 | 1.00 |
| S | Serine protease 27 | 1.17 | 0.28 | 1.00 |  | 5.88 | 0.02 | 0.17 |  | 4.98 | 0.03 | 1.00 |  | 0.90 | 0.34 | 1.00 |  | 2.26 | 0.13 | 1.00 |
| S | Serine protease 28 | 7.31 | 0.01 | 0.91 |  | 7.14 | 0.01 | 0.10 |  | 0.79 | 0.37 | 1.00 |  | 1.42 | 0.23 | 1.00 |  | 0.00 | 0.95 | 1.00 |
| S | Serine protease 29 | 0.00 | 0.98 | 1.00 |  | 1.48 | 0.22 | 0.85 |  | 2.73 | 0.10 | 1.00 |  | 0.00 | 0.96 | 1.00 |  | 6.29 | 0.01 | 1.00 |
| S | Serine protease 30 | 0.35 | 0.55 | 1.00 |  | 0.70 | 0.40 | 1.00 |  | 0.92 | 0.34 | 1.00 |  | 0.12 | 0.73 | 1.00 |  | 0.55 | 0.46 | 1.00 |
| S | Serine protease 31 | 2.31 | 0.13 | 1.00 |  | 0.98 | 0.32 | 0.94 |  | 1.19 | 0.28 | 1.00 |  | 0.25 | 0.62 | 1.00 |  | 0.13 | 0.72 | 1.00 |
| S | Serine protease 32 | 2.47 | 0.12 | 1.00 |  | 1.26 | 0.26 | 0.85 |  | **38.37** | **0.00** | **0.00** |  | 4.67 | 0.03 | 1.00 |  | 3.49 | 0.06 | 1.00 |
| S | Serine protease 33 | 1.19 | 0.28 | 1.00 |  | 2.83 | 0.09 | 0.65 |  | 0.05 | 0.83 | 1.00 |  | 0.13 | 0.72 | 1.00 |  | 4.88 | 0.03 | 1.00 |
| S | Serine protease 34 | 0.20 | 0.65 | 1.00 |  | 5.64 | 0.02 | 0.19 |  | 0.17 | 0.68 | 1.00 |  | 0.14 | 0.71 | 1.00 |  | 0.67 | 0.41 | 1.00 |
| S | Serine protease 35 | 1.63 | 0.20 | 1.00 |  | 1.71 | 0.19 | 0.85 |  | 8.18 | 0.00 | 0.35 |  | 0.52 | 0.47 | 1.00 |  | 0.04 | 0.84 | 1.00 |
| S | Serine protease 36 | 1.61 | 0.20 | 1.00 |  | 2.35 | 0.13 | 0.73 |  | 12.32 | 0.00 | 0.06 |  | 0.10 | 0.75 | 1.00 |  | 0.01 | 0.91 | 1.00 |
| S | Serine protease 37 | 0.39 | 0.53 | 1.00 |  | **16.24** | **0.00** | **0.00** |  | 3.52 | 0.06 | 1.00 |  | 0.48 | 0.49 | 1.00 |  | 1.42 | 0.23 | 1.00 |
| S | Serine protease 38 | 0.24 | 0.63 | 1.00 |  | 4.49 | 0.03 | 0.33 |  | 2.32 | 0.13 | 1.00 |  | 0.00 | 0.99 | 1.00 |  | 1.13 | 0.29 | 1.00 |
| S | Serine protease 39 | 0.90 | 0.34 | 1.00 |  | 1.81 | 0.18 | 0.85 |  | 2.38 | 0.12 | 1.00 |  | 0.05 | 0.83 | 1.00 |  | 0.00 | 0.98 | 1.00 |
| S | Serine protease 40 | 0.79 | 0.37 | 1.00 |  | 4.50 | 0.03 | 0.33 |  | 0.40 | 0.53 | 1.00 |  | 0.56 | 0.45 | 1.00 |  | 0.10 | 0.76 | 1.00 |
| S | Serine protease 41 | **16.45** | **0.00** | **0.04** |  | **119.72** | **0.00** | **0.00** |  | **98.65** | **0.00** | **0.00** |  | 0.00 | 0.95 | 1.00 |  | 0.01 | 0.94 | 1.00 |
| S | Serine protease 42 | 0.19 | 0.66 | 1.00 |  | 0.38 | 0.54 | 1.00 |  | 2.51 | 0.11 | 1.00 |  | 0.25 | 0.61 | 1.00 |  | 0.55 | 0.46 | 1.00 |
| S | Serine protease 43 | 3.12 | 0.08 | 1.00 |  | 1.50 | 0.22 | 0.85 |  | 2.27 | 0.13 | 1.00 |  | 5.01 | 0.03 | 1.00 |  | 0.29 | 0.59 | 1.00 |
| S | Serine protease 44 | 0.00 | 1.00 | 1.00 |  | **19.12** | **0.00** | **0.00** |  | 1.17 | 0.28 | 1.00 |  | 1.52 | 0.22 | 1.00 |  | 1.34 | 0.25 | 1.00 |
| S | Serine protease 45 | 1.14 | 0.28 | 1.00 |  | 6.39 | 0.01 | 0.14 |  | 3.47 | 0.06 | 1.00 |  | 0.01 | 0.92 | 1.00 |  | 10.01 | 0.00 | 0.60 |
| S | Serine protease 46 | 2.04 | 0.15 | 1.00 |  | **54.22** | **0.00** | **0.00** |  | 2.60 | 0.11 | 1.00 |  | **43.72** | **0.00** | **0.00** |  | 2.96 | 0.09 | 1.00 |
| S | Serine protease 47 | 0.83 | 0.36 | 1.00 |  | 2.54 | 0.11 | 0.71 |  | 0.37 | 0.54 | 1.00 |  | 0.30 | 0.58 | 1.00 |  | 1.01 | 0.31 | 1.00 |
| S | Serine protease 48 | 0.08 | 0.77 | 1.00 |  | 1.35 | 0.25 | 0.85 |  | **15.64** | **0.00** | **0.01** |  | 3.19 | 0.07 | 1.00 |  | 12.29 | 0.00 | 0.35 |
| S | Serine protease 49 | 1.14 | 0.28 | 1.00 |  | 0.00 | 0.98 | 1.00 |  | 2.28 | 0.13 | 1.00 |  | 1.05 | 0.30 | 1.00 |  | 3.60 | 0.06 | 1.00 |
| S | Serine protease 50 | 0.15 | 0.70 | 1.00 |  | 0.05 | 0.82 | 1.00 |  | 1.58 | 0.21 | 1.00 |  | 1.23 | 0.27 | 1.00 |  | 9.23 | 0.00 | 0.76 |
| S | Serine protease 51 | 0.32 | 0.57 | 1.00 |  | 0.26 | 0.61 | 1.00 |  | 6.45 | 0.01 | 0.73 |  | 1.51 | 0.22 | 1.00 |  | 0.16 | 0.69 | 1.00 |
| S | Serine protease 52 | 1.70 | 0.19 | 1.00 |  | 3.62 | 0.06 | 0.47 |  | 0.16 | 0.69 | 1.00 |  | 2.68 | 0.10 | 1.00 |  | 2.69 | 0.10 | 1.00 |
| S | Serine protease 53 | 0.93 | 0.34 | 1.00 |  | 4.43 | 0.04 | 0.34 |  | 0.03 | 0.85 | 1.00 |  | 0.04 | 0.85 | 1.00 |  | 0.42 | 0.52 | 1.00 |
| S | Serine protease 54 | 2.02 | 0.16 | 1.00 |  | 1.03 | 0.31 | 0.91 |  | 2.66 | 0.10 | 1.00 |  | 2.20 | 0.14 | 1.00 |  | 2.64 | 0.10 | 1.00 |
| S | Serine protease 55 | 0.33 | 0.57 | 1.00 |  | 0.03 | 0.87 | 1.00 |  | 0.80 | 0.37 | 1.00 |  | 0.04 | 0.85 | 1.00 |  | 1.12 | 0.29 | 1.00 |
| S | Serine protease 56 | 0.27 | 0.61 | 1.00 |  | 0.87 | 0.35 | 1.00 |  | 2.72 | 0.10 | 1.00 |  | 8.06 | 0.00 | 0.55 |  | 3.90 | 0.05 | 1.00 |
| S | Serine protease 57 | 0.46 | 0.50 | 1.00 |  | 1.71 | 0.19 | 0.85 |  | 2.56 | 0.11 | 1.00 |  | 0.01 | 0.92 | 1.00 |  | 0.90 | 0.34 | 1.00 |
| S | Serine protease 58 | 6.96 | 0.01 | 1.00 |  | 5.37 | 0.02 | 0.22 |  | **90.60** | **0.00** | **0.00** |  | 1.67 | 0.20 | 1.00 |  | 2.44 | 0.12 | 1.00 |
| S | Serine protease 59 | 0.00 | 0.95 | 1.00 |  | 2.75 | 0.10 | 0.67 |  | 2.15 | 0.14 | 1.00 |  | 0.27 | 0.60 | 1.00 |  | 1.65 | 0.20 | 1.00 |
| S | Serine protease 60 | 4.87 | 0.03 | 1.00 |  | 5.30 | 0.02 | 0.22 |  | 11.43 | 0.00 | 0.09 |  | 0.01 | 0.94 | 1.00 |  | 0.01 | 0.91 | 1.00 |
| S | Serine protease 61 | 3.73 | 0.05 | 1.00 |  | 1.12 | 0.29 | 0.88 |  | 2.61 | 0.11 | 1.00 |  | 0.30 | 0.58 | 1.00 |  | 1.02 | 0.31 | 1.00 |
| S | Serine protease 62 | **88.84** | **0.00** | **0.00** |  | **235.35** | **0.00** | **0.00** |  | **44.71** | **0.00** | **0.00** |  | 1.18 | 0.28 | 1.00 |  | 0.02 | 0.89 | 1.00 |
| S | Serine protease 63 | 3.92 | 0.05 | 1.00 |  | **32.76** | **0.00** | **0.00** |  | **15.06** | **0.00** | **0.02** |  | 5.80 | 0.02 | 1.00 |  | 0.07 | 0.80 | 1.00 |
| S | Serine protease 64 | 4.85 | 0.03 | 1.00 |  | 0.85 | 0.36 | 1.00 |  | 10.86 | 0.00 | 0.11 |  | 1.74 | 0.19 | 1.00 |  | 2.18 | 0.14 | 1.00 |
| S | Serine protease 65 | 0.05 | 0.82 | 1.00 |  | 0.44 | 0.51 | 1.00 |  | 0.40 | 0.53 | 1.00 |  | 0.04 | 0.85 | 1.00 |  | 1.07 | 0.30 | 1.00 |
| S | Serine protease 66 | **120.46** | **0.00** | **0.00** |  | **1250.12** | **0.00** | **0.00** |  | **200.41** | **0.00** | **0.00** |  | 5.07 | 0.02 | 1.00 |  | 0.28 | 0.60 | 1.00 |
| S | Serine protease 67 | 1.68 | 0.19 | 1.00 |  | 0.17 | 0.68 | 1.00 |  | 0.54 | 0.46 | 1.00 |  | 0.25 | 0.62 | 1.00 |  | 0.03 | 0.86 | 1.00 |
| S | Serine protease 68 | 0.23 | 0.63 | 1.00 |  | 0.00 | 0.96 | 1.00 |  | 1.63 | 0.20 | 1.00 |  | 0.27 | 0.60 | 1.00 |  | 0.61 | 0.44 | 1.00 |
| S | Serine protease 69 | 2.02 | 0.16 | 1.00 |  | 3.46 | 0.06 | 0.51 |  | 4.98 | 0.03 | 1.00 |  | **33.02** | **0.00** | **0.00** |  | 0.04 | 0.85 | 1.00 |
| S | Serine protease 70 | 5.96 | 0.01 | 1.00 |  | 0.10 | 0.75 | 1.00 |  | 5.98 | 0.01 | 0.86 |  | **17.36** | **0.00** | **0.02** |  | 0.04 | 0.85 | 1.00 |
| S | Kazal-type serine protease inhibitor domain-containing protein 1 | 6.51 | 0.01 | 1.00 |  | 2.15 | 0.14 | 0.78 |  | 0.70 | 0.40 | 1.00 |  | 0.02 | 0.88 | 1.00 |  | 0.02 | 0.88 | 1.00 |
| S | Serine protease inhibitor 1 | 2.31 | 0.13 | 1.00 |  | 0.98 | 0.32 | 0.94 |  | 1.19 | 0.28 | 1.00 |  | 0.25 | 0.62 | 1.00 |  | 0.13 | 0.72 | 1.00 |
| S | Serine protease inhibitor 2 | 0.12 | 0.73 | 1.00 |  | 1.08 | 0.30 | 0.89 |  | 3.97 | 0.05 | 1.00 |  | 1.08 | 0.30 | 1.00 |  | 0.05 | 0.83 | 1.00 |
| S | Serine protease inhibitor 3 | 0.93 | 0.34 | 1.00 |  | 4.43 | 0.04 | 0.34 |  | 0.03 | 0.85 | 1.00 |  | 0.04 | 0.85 | 1.00 |  | 0.42 | 0.52 | 1.00 |
| S | Serine protease inhibitor 4 | 0.05 | 0.82 | 1.00 |  | 0.44 | 0.51 | 1.00 |  | 0.40 | 0.53 | 1.00 |  | 0.04 | 0.85 | 1.00 |  | 1.07 | 0.30 | 1.00 |
| S | Serine protease inhibitor dipetalogastin 1 | 2.01 | 0.16 | 1.00 |  | **18.61** | **0.00** | **0.00** |  | 2.14 | 0.14 | 1.00 |  | 1.79 | 0.18 | 1.00 |  | 8.29 | 0.00 | 0.89 |
| S | Serine protease inhibitor dipetalogastin 2 | 0.51 | 0.48 | 1.00 |  | **13.86** | **0.00** | **0.00** |  | 8.99 | 0.00 | 0.25 |  | 0.26 | 0.61 | 1.00 |  | 0.01 | 0.92 | 1.00 |
| S | Prophenoloxidase activating factor 1 | 0.09 | 0.76 | 1.00 |  | 6.22 | 0.01 | 0.15 |  | 2.22 | 0.14 | 1.00 |  | 1.17 | 0.28 | 1.00 |  | 1.03 | 0.31 | 1.00 |
| S | 14-3-3 protein 1 | 0.01 | 0.90 | 1.00 |  | 1.54 | 0.21 | 0.85 |  | 0.35 | 0.55 | 1.00 |  | 1.58 | 0.21 | 1.00 |  | 0.37 | 0.54 | 1.00 |
| S | 14-3-3 protein 2 | 0.25 | 0.61 | 1.00 |  | 3.45 | 0.06 | 0.51 |  | 0.06 | 0.81 | 1.00 |  | 0.02 | 0.88 | 1.00 |  | 0.44 | 0.51 | 1.00 |
| S | 14-3-3 protein 3 | 0.07 | 0.80 | 1.00 |  | **9.07** | **0.00** | **0.04** |  | **24.26** | **0.00** | **0.00** |  | 0.01 | 0.90 | 1.00 |  | 3.54 | 0.06 | 1.00 |
| S | Calpain 1 | 2.89 | 0.09 | 1.00 |  | 1.60 | 0.21 | 0.85 |  | 8.27 | 0.00 | 0.34 |  | 2.71 | 0.10 | 1.00 |  | 5.39 | 0.02 | 1.00 |
| S | Calpain 2 | 4.25 | 0.04 | 1.00 |  | 0.05 | 0.82 | 1.00 |  | 4.93 | 0.03 | 1.00 |  | 1.34 | 0.25 | 1.00 |  | 1.21 | 0.27 | 1.00 |
| S | Calpain 3 | 3.05 | 0.08 | 1.00 |  | **64.73** | **0.00** | **0.00** |  | 5.51 | 0.02 | 1.00 |  | 0.30 | 0.58 | 1.00 |  | 0.00 | 0.98 | 1.00 |
| S | Calpain 4 | 2.36 | 0.12 | 1.00 |  | 8.35 | 0.00 | 0.06 |  | 3.08 | 0.08 | 1.00 |  | 8.50 | 0.00 | 0.45 |  | 0.30 | 0.58 | 1.00 |
| S | Calpain 5 | 0.53 | 0.47 | 1.00 |  | 0.21 | 0.65 | 1.00 |  | 3.11 | 0.08 | 1.00 |  | 0.18 | 0.67 | 1.00 |  | 0.06 | 0.81 | 1.00 |
| S | Minor histocompatibility antigen 1 | 5.51 | 0.02 | 1.00 |  | 5.93 | 0.01 | 0.17 |  | 11.77 | 0.00 | 0.07 |  | 3.72 | 0.05 | 1.00 |  | 3.38 | 0.07 | 1.00 |
| S | Minor histocompatibility antigen 2 | 2.66 | 0.10 | 1.00 |  | 1.46 | 0.23 | 0.85 |  | **23.95** | **0.00** | **0.00** |  | 0.99 | 0.32 | 1.00 |  | 0.38 | 0.54 | 1.00 |
| S | Low-density lipoprotein receptor-related protein 1 | 0.00 | 1.00 | 1.00 |  | 1.44 | 0.23 | 0.85 |  | 0.10 | 0.75 | 1.00 |  | 0.76 | 0.38 | 1.00 |  | 1.27 | 0.26 | 1.00 |
| S | Low-density lipoprotein receptor-related protein 2 | 1.40 | 0.24 | 1.00 |  | 0.09 | 0.77 | 1.00 |  | **28.85** | **0.00** | **0.00** |  | 5.69 | 0.02 | 1.00 |  | 6.26 | 0.01 | 1.00 |
| S | Low-density lipoprotein receptor-related protein 3 | **53.49** | **0.00** | **0.00** |  | **205.37** | **0.00** | **0.00** |  | **149.56** | **0.00** | **0.00** |  | 0.41 | 0.52 | 1.00 |  | 0.00 | 0.97 | 1.00 |
| S | Low-density lipoprotein receptor-related protein 4 | 0.06 | 0.80 | 1.00 |  | 4.41 | 0.04 | 0.34 |  | 2.35 | 0.12 | 1.00 |  | 3.63 | 0.06 | 1.00 |  | 5.19 | 0.02 | 1.00 |
| S | Low-density lipoprotein receptor-related protein 5 | 0.34 | 0.56 | 1.00 |  | **12.55** | **0.00** | **0.01** |  | 11.96 | 0.00 | 0.07 |  | **18.97** | **0.00** | **0.01** |  | 0.01 | 0.94 | 1.00 |
| S | Low-density lipoprotein receptor-related protein 6 | 11.60 | 0.00 | 0.24 |  | **51.27** | **0.00** | **0.00** |  | **192.42** | **0.00** | **0.00** |  | 1.15 | 0.28 | 1.00 |  | 6.51 | 0.01 | 1.00 |
| S | Low-density lipoprotein receptor-related protein 7 | 12.66 | 0.00 | 0.16 |  | 3.07 | 0.08 | 0.58 |  | 3.87 | 0.05 | 1.00 |  | 3.43 | 0.06 | 1.00 |  | 0.01 | 0.93 | 1.00 |
| S | Low-density lipoprotein receptor-related protein 8 | 0.72 | 0.40 | 1.00 |  | 0.22 | 0.64 | 1.00 |  | **13.38** | **0.00** | **0.04** |  | 4.97 | 0.03 | 1.00 |  | 1.04 | 0.31 | 1.00 |
| S | Four and a half LIM domains protein 1 | 3.41 | 0.06 | 1.00 |  | 5.99 | 0.01 | 0.17 |  | 0.01 | 0.91 | 1.00 |  | 0.05 | 0.83 | 1.00 |  | 0.54 | 0.46 | 1.00 |
| E | Carboxypeptidase 1 | 0.31 | 0.58 | 1.00 |  | 0.49 | 0.49 | 1.00 |  | 0.00 | 0.99 | 1.00 |  | 1.03 | 0.31 | 1.00 |  | 1.43 | 0.23 | 1.00 |
| E | Carboxypeptidase 2 | 0.13 | 0.71 | 1.00 |  | 0.01 | 0.92 | 1.00 |  | **19.64** | **0.00** | **0.00** |  | 0.54 | 0.46 | 1.00 |  | 0.01 | 0.91 | 1.00 |
| E | Carboxypeptidase 3 | 0.52 | 0.47 | 1.00 |  | 1.95 | 0.16 | 0.85 |  | **29.30** | **0.00** | **0.00** |  | 3.64 | 0.06 | 1.00 |  | 0.49 | 0.49 | 1.00 |
| E | Carboxypeptidase 4 | 8.21 | 0.00 | 0.71 |  | 1.11 | 0.29 | 0.88 |  | 9.70 | 0.00 | 0.19 |  | 0.95 | 0.33 | 1.00 |  | 0.29 | 0.59 | 1.00 |
| E | Carboxypeptidase 5 | 0.05 | 0.83 | 1.00 |  | 0.29 | 0.59 | 1.00 |  | 0.67 | 0.41 | 1.00 |  | 0.60 | 0.44 | 1.00 |  | 2.33 | 0.13 | 1.00 |
| E | Carboxypeptidase 6 | 8.00 | 0.00 | 0.74 |  | 0.67 | 0.41 | 1.00 |  | 0.94 | 0.33 | 1.00 |  | 9.20 | 0.00 | 0.38 |  | 0.45 | 0.50 | 1.00 |
| E | Carboxypeptidase 7 | 1.06 | 0.30 | 1.00 |  | 8.43 | 0.00 | 0.06 |  | 0.05 | 0.82 | 1.00 |  | 0.04 | 0.84 | 1.00 |  | 0.74 | 0.39 | 1.00 |
| E | Carboxypeptidase 8 | 0.55 | 0.46 | 1.00 |  | 1.65 | 0.20 | 0.85 |  | 4.53 | 0.03 | 1.00 |  | **18.54** | **0.00** | **0.01** |  | 0.30 | 0.58 | 1.00 |
| E | Carboxypeptidase 9 | 0.10 | 0.76 | 1.00 |  | 0.58 | 0.45 | 1.00 |  | 0.92 | 0.34 | 1.00 |  | 2.87 | 0.09 | 1.00 |  | 2.20 | 0.14 | 1.00 |
| E | Carboxypeptidase 10 | 2.01 | 0.16 | 1.00 |  | 4.32 | 0.04 | 0.35 |  | 3.34 | 0.07 | 1.00 |  | 0.13 | 0.72 | 1.00 |  | 0.57 | 0.45 | 1.00 |
| E | Carboxypeptidase 11 | 1.31 | 0.25 | 1.00 |  | 1.79 | 0.18 | 0.85 |  | 1.52 | 0.22 | 1.00 |  | 3.44 | 0.06 | 1.00 |  | 0.16 | 0.69 | 1.00 |
| E | Cathepsin 1 | 0.00 | 1.00 | 1.00 |  | 0.00 | 1.00 | 1.00 |  | 0.00 | 1.00 | 1.00 |  | **25.58** | **0.00** | **0.00** |  | 0.00 | 1.00 | 1.00 |
| E | Cathepsin 2 | 0.00 | 1.00 | 1.00 |  | 0.00 | 1.00 | 1.00 |  | 0.00 | 1.00 | 1.00 |  | **72.88** | **0.00** | **0.00** |  | 0.00 | 1.00 | 1.00 |
| E | Cathepsin 3 | 0.00 | 1.00 | 1.00 |  | **10.70** | **0.00** | **0.02** |  | 0.00 | 1.00 | 1.00 |  | 0.00 | 1.00 | 1.00 |  | 0.00 | 1.00 | 1.00 |
| E | Cathepsin 4 | 0.06 | 0.81 | 1.00 |  | 1.14 | 0.29 | 0.87 |  | 0.35 | 0.55 | 1.00 |  | 0.87 | 0.35 | 1.00 |  | 1.04 | 0.31 | 1.00 |
| E | Cathepsin 5 | 4.04 | 0.04 | 1.00 |  | 0.67 | 0.41 | 1.00 |  | 12.69 | 0.00 | 0.05 |  | 1.06 | 0.30 | 1.00 |  | 0.03 | 0.86 | 1.00 |
| E | Cathepsin 6 | 1.11 | 0.29 | 1.00 |  | 4.33 | 0.04 | 0.35 |  | 3.83 | 0.05 | 1.00 |  | 0.00 | 0.98 | 1.00 |  | 0.03 | 0.86 | 1.00 |
| E | Cathepsin 7 | 0.79 | 0.37 | 1.00 |  | 4.50 | 0.03 | 0.33 |  | 0.40 | 0.53 | 1.00 |  | 0.56 | 0.45 | 1.00 |  | 0.10 | 0.76 | 1.00 |
| E | Cathepsin 8 | 0.69 | 0.41 | 1.00 |  | 3.22 | 0.07 | 0.55 |  | 1.40 | 0.24 | 1.00 |  | 3.95 | 0.05 | 1.00 |  | 0.56 | 0.45 | 1.00 |
| E | Cathepsin 9 | 0.48 | 0.49 | 1.00 |  | 1.73 | 0.19 | 0.85 |  | 5.02 | 0.03 | 1.00 |  | 0.50 | 0.48 | 1.00 |  | 0.10 | 0.75 | 1.00 |
| E | Cathepsin 10 | 0.05 | 0.82 | 1.00 |  | 0.04 | 0.84 | 1.00 |  | 0.00 | 1.00 | 1.00 |  | 3.12 | 0.08 | 1.00 |  | 0.19 | 0.66 | 1.00 |
| E | Lysozyme C type 1 | 0.27 | 0.61 | 1.00 |  | 2.02 | 0.16 | 0.83 |  | 9.96 | 0.00 | 0.16 |  | 0.09 | 0.77 | 1.00 |  | 0.06 | 0.81 | 1.00 |
| E | Lysozyme-like protein | 1.14 | 0.28 | 1.00 |  | 0.42 | 0.52 | 1.00 |  | 2.29 | 0.13 | 1.00 |  | 7.47 | 0.01 | 0.66 |  | 0.63 | 0.43 | 1.00 |
| E | Lysozyme P type | 1.58 | 0.21 | 1.00 |  | **123.73** | **0.00** | **0.00** |  | **61.07** | **0.00** | **0.00** |  | 0.06 | 0.80 | 1.00 |  | 0.39 | 0.53 | 1.00 |
| E | Lysozyme C type 2 | 0.64 | 0.42 | 1.00 |  | 5.91 | 0.02 | 0.17 |  | **40.17** | **0.00** | **0.00** |  | 1.86 | 0.17 | 1.00 |  | 1.68 | 0.19 | 1.00 |
| E | Lysozyme C type-like protein | 3.23 | 0.07 | 1.00 |  | 0.06 | 0.81 | 1.00 |  | **16.01** | **0.00** | **0.01** |  | 4.81 | 0.03 | 1.00 |  | 0.86 | 0.35 | 1.00 |
| E | Lysozyme I type 1 | 0.37 | 0.54 | 1.00 |  | 0.17 | 0.68 | 1.00 |  | **21.92** | **0.00** | **0.00** |  | 1.49 | 0.22 | 1.00 |  | 1.01 | 0.31 | 1.00 |
| E | Lysozyme I type 2 | 0.73 | 0.39 | 1.00 |  | 0.27 | 0.60 | 1.00 |  | **17.08** | **0.00** | **0.01** |  | 0.54 | 0.46 | 1.00 |  | 0.18 | 0.67 | 1.00 |
| E | Lysozyme I type 3 | 1.34 | 0.25 | 1.00 |  | 0.00 | 0.99 | 1.00 |  | 7.20 | 0.01 | 0.51 |  | 0.01 | 0.93 | 1.00 |  | 2.42 | 0.12 | 1.00 |
| E | Lysozyme C type 3 | 12.53 | 0.00 | 0.17 |  | 6.32 | 0.01 | 0.14 |  | 3.22 | 0.07 | 1.00 |  | 2.28 | 0.13 | 1.00 |  | 0.01 | 0.93 | 1.00 |
| E | Metacaspase-like cysteine peptidase 1 | 1.13 | 0.29 | 1.00 |  | 3.94 | 0.05 | 0.41 |  | 0.00 | 1.00 | 1.00 |  | 4.46 | 0.03 | 1.00 |  | 9.09 | 0.00 | 0.79 |
| E | Metacaspase-like cysteine peptidase 2 | 0.51 | 0.47 | 1.00 |  | **13.30** | **0.00** | **0.01** |  | 0.00 | 1.00 | 1.00 |  | 4.25 | 0.04 | 1.00 |  | 0.28 | 0.60 | 1.00 |
| E | Asparaginyl endopeptidase-like cysteine peptidase 1 | 1.95 | 0.16 | 1.00 |  | **10.07** | **0.00** | **0.03** |  | 0.00 | 1.00 | 1.00 |  | 12.98 | 0.00 | 0.09 |  | 0.22 | 0.64 | 1.00 |
| E | Lysosomal Pro-X carboxypeptidase 1 | 0.64 | 0.42 | 1.00 |  | 0.04 | 0.85 | 1.00 |  | 1.28 | 0.26 | 1.00 |  | 0.04 | 0.84 | 1.00 |  | 0.09 | 0.76 | 1.00 |
| E | Prolixicin antimicrobial protein 1 | 0.02 | 0.88 | 1.00 |  | 0.36 | 0.55 | 1.00 |  | **123.90** | **0.00** | **0.00** |  | 104.75 | 0.00 | 0.00 |  | 10.71 | 0.00 | 0.54 |
| E | Transferrin 1 | 6.83 | 0.01 | 1.00 |  | 3.72 | 0.05 | 0.45 |  | **14.50** | **0.00** | **0.02** |  | 0.15 | 0.70 | 1.00 |  | 3.54 | 0.06 | 1.00 |
| E | Transferrin 2 | 1.95 | 0.16 | 1.00 |  | **29.66** | **0.00** | **0.00** |  | 8.34 | 0.00 | 0.33 |  | 0.13 | 0.72 | 1.00 |  | 0.52 | 0.47 | 1.00 |
| E | Transferrin 3 | 4.60 | 0.03 | 1.00 |  | **9.61** | **0.00** | **0.03** |  | **54.79** | **0.00** | **0.00** |  | 0.05 | 0.83 | 1.00 |  | 3.10 | 0.08 | 1.00 |
| E | Termicin 1 | **19.16** | **0.00** | **0.01** |  | 0.26 | 0.61 | 1.00 |  | **95.34** | **0.00** | **0.00** |  | 1.15 | 0.28 | 1.00 |  | 1.49 | 0.22 | 1.00 |
| E | Cysteine-rich protein 1 | 7.82 | 0.01 | 0.74 |  | 1.58 | 0.21 | 0.85 |  | **38.59** | **0.00** | **0.00** |  | 0.14 | 0.71 | 1.00 |  | 0.48 | 0.49 | 1.00 |
| E | Cysteine-rich protein 2 | 0.00 | 1.00 | 1.00 |  | 2.83 | 0.09 | 0.65 |  | 0.00 | 1.00 | 1.00 |  | 0.00 | 1.00 | 1.00 |  | 2.21 | 0.14 | 1.00 |
| E | Cysteine-rich protein 3 | 0.48 | 0.49 | 1.00 |  | 0.38 | 0.54 | 1.00 |  | 4.58 | 0.03 | 1.00 |  | 0.34 | 0.56 | 1.00 |  | 0.25 | 0.62 | 1.00 |
| E | Cysteine-rich protein 4 | 0.05 | 0.82 | 1.00 |  | 0.08 | 0.77 | 1.00 |  | 0.11 | 0.74 | 1.00 |  | 0.88 | 0.35 | 1.00 |  | 0.24 | 0.62 | 1.00 |
| E | Cysteine-rich protein 5 | 3.69 | 0.05 | 1.00 |  | **19.96** | **0.00** | **0.00** |  | 0.05 | 0.82 | 1.00 |  | 0.35 | 0.55 | 1.00 |  | 0.22 | 0.64 | 1.00 |
| E | Cysteine-rich protein 6 | 0.03 | 0.86 | 1.00 |  | 3.42 | 0.06 | 0.51 |  | 2.46 | 0.12 | 1.00 |  | 0.30 | 0.58 | 1.00 |  | 0.62 | 0.43 | 1.00 |
| E | Cysteine-rich protein 7 | 4.43 | 0.04 | 1.00 |  | 0.16 | 0.69 | 1.00 |  | 3.53 | 0.06 | 1.00 |  | 0.00 | 0.98 | 1.00 |  | 0.09 | 0.76 | 1.00 |
| E | Cysteine-rich protein 8 | 1.73 | 0.19 | 1.00 |  | **9.87** | **0.00** | **0.03** |  | **22.99** | **0.00** | **0.00** |  | 0.84 | 0.36 | 1.00 |  | 0.27 | 0.61 | 1.00 |
| E | Cysteine-rich protein 9 | 2.45 | 0.12 | 1.00 |  | 0.83 | 0.36 | 1.00 |  | **16.67** | **0.00** | **0.01** |  | 0.49 | 0.48 | 1.00 |  | 0.26 | 0.61 | 1.00 |
| E | Cysteine-rich protein 10 | 0.12 | 0.73 | 1.00 |  | 1.59 | 0.21 | 0.85 |  | 6.65 | 0.01 | 0.67 |  | 0.77 | 0.38 | 1.00 |  | 0.23 | 0.63 | 1.00 |
| E | Cysteine-rich protein 11 | 4.19 | 0.04 | 1.00 |  | **16.39** | **0.00** | **0.00** |  | 0.66 | 0.42 | 1.00 |  | 9.30 | 0.00 | 0.37 |  | 1.41 | 0.24 | 1.00 |
| E | Cysteine-rich protein 12 | 13.37 | 0.00 | 0.13 |  | **149.00** | **0.00** | **0.00** |  | **188.11** | **0.00** | **0.00** |  | 3.07 | 0.08 | 1.00 |  | 0.63 | 0.43 | 1.00 |
| E | Cysteine-rich protein 13 | 0.03 | 0.86 | 1.00 |  | 3.42 | 0.06 | 0.51 |  | 2.46 | 0.12 | 1.00 |  | 0.30 | 0.58 | 1.00 |  | 0.62 | 0.43 | 1.00 |
| E | Cysteine-rich protein 14 | 4.11 | 0.04 | 1.00 |  | 1.30 | 0.25 | 0.85 |  | 4.42 | 0.04 | 1.00 |  | **89.57** | **0.00** | **0.00** |  | 0.23 | 0.63 | 1.00 |
| E | Ferritin 1 | 0.00 | 1.00 | 1.00 |  | 0.00 | 1.00 | 1.00 |  | 0.00 | 1.00 | 1.00 |  | **28.65** | **0.00** | **0.00** |  | 0.00 | 1.00 | 1.00 |
| E | Ferritin 2 | 1.30 | 0.25 | 1.00 |  | 2.35 | 0.13 | 0.73 |  | 0.00 | 1.00 | 1.00 |  | 0.00 | 1.00 | 1.00 |  | 0.00 | 1.00 | 1.00 |
| E | Ferritin 3 | 1.69 | 0.19 | 1.00 |  | 2.55 | 0.11 | 0.71 |  | 12.82 | 0.00 | 0.05 |  | 0.84 | 0.36 | 1.00 |  | 0.32 | 0.57 | 1.00 |
| E | Ferritin 4 | 1.99 | 0.16 | 1.00 |  | 4.47 | 0.03 | 0.33 |  | 11.71 | 0.00 | 0.08 |  | 1.02 | 0.31 | 1.00 |  | 1.45 | 0.23 | 1.00 |
| E | Melanotransferrin 1 | 1.93 | 0.16 | 1.00 |  | 0.32 | 0.57 | 1.00 |  | 1.42 | 0.23 | 1.00 |  | 0.12 | 0.73 | 1.00 |  | 1.17 | 0.28 | 1.00 |
| E | Venom allergen 1 | 0.04 | 0.84 | 1.00 |  | **11.23** | **0.00** | **0.02** |  | **28.06** | **0.00** | **0.00** |  | 0.00 | 0.98 | 1.00 |  | 0.09 | 0.76 | 1.00 |
| E | Thaumatin-like protein 1 | 0.99 | 0.32 | 1.00 |  | 1.89 | 0.17 | 0.85 |  | 4.88 | 0.03 | 1.00 |  | 0.32 | 0.57 | 1.00 |  | 0.65 | 0.42 | 1.00 |

Comparison of normalized counts per million (CPM) between sexes in each caste (PK: primary king, PQ: primary queens, SQ: secondary queen) was conducted using edgeR package. Bold letters mean significant difference (FDR < 0.05), and red and blue letters indicate the female- and male-biased expression, respectively. LR: likelihood ratio, FDR: false discovery rate, PRP: pattern recognition protein, S: signalling protein, E: Effector.
