# Supplementary material for: Caste-, sex-, and age-dependent expression of immune-related genes in a Japanese subterranean termite, Reticulitermes speratus
Source: PLoS One. 2017 Apr 14;12(4):e0175417. doi: 10.1371/journal.pone.0175417 (PMC5391962; doi:10.1371/journal.pone.0175417)
Supplement: S4 Table — Comparison of normalized counts per million (CPM) among male reproductives (alates (A), young primary kings (Y) and mature primary kings (M)) or female ones (alates (A) and young primary queens (Y)) was conducted using edgeR package. Bold letters mean significant difference (FDR < 0.05). “Caste showing the highest expression” means the caste showing the largest mean CPM among all age classes for each gene, and the largest mean CPM in a certain age class is more than 1.2 times as high as the mean CPM in the other classes. LR: likelihood ratio, FDR: false discovery rate, PRP: pattern recognition protein, S: Signalling protein, E: effector. (DOCX) [file pone.0175417.s008.docx]

**Table S4. Statistical results of age-dependent expression changes in male or female reproductives.**

| Functional category | Gene name | Male | | | |  | Female | | | |
| --- | --- | --- | --- | --- | --- | --- | --- | --- | --- | --- |
|  |  | LR | P-value | FDR | Caste showing the highest expression |  | LR | P-value | FDR | Caste showing the highest expression |
| PRP | ApolipophorinIII 1 | **26.21** | **0.00** | **0.00** | M |  | **61.71** | **0.00** | **0.00** | Y |
| PRP | ApolipophorinIII 2 | **41.85** | **0.00** | **0.00** | Y |  | **60.74** | **0.00** | **0.00** | Y |
| PRP | ApolipophorinIII 3 | **88.22** | **0.00** | **0.00** | Y |  | 3.01 | 0.08 | 0.70 |  |
| PRP | Brevican 1 | **102.79** | **0.00** | **0.00** | A, M |  | **23.73** | **0.00** | **0.00** | A |
| PRP | C-type lectin-like domain protein 1 | **20.82** | **0.00** | **0.00** | M |  | 0.01 | 0.94 | 1.00 |  |
| PRP | C-type lectin-like domain protein 2 | 8.04 | 0.02 | 0.18 |  |  | 2.47 | 0.12 | 0.89 |  |
| PRP | C-type lectin-like domain protein 3 | **30.87** | **0.00** | **0.00** | A, M |  | **22.74** | **0.00** | **0.00** | Y |
| PRP | C-type lectin-like domain protein 4 | **1995.25** | **0.00** | **0.00** | M |  | **125.55** | **0.00** | **0.00** | A |
| PRP | C-type lectin-like domain protein 5 | 10.54 | 0.01 | 0.06 |  |  | 2.31 | 0.13 | 0.96 |  |
| PRP | C-type lectin-like domain protein 6 | 1.64 | 0.44 | 1.00 |  |  | 2.34 | 0.13 | 0.94 |  |
| PRP | C-type lectin-like domain protein 7 | **98.48** | **0.00** | **0.00** | Y |  | 1.81 | 0.18 | 1.00 |  |
| PRP | C-type lectin-like domain protein 8 | 3.70 | 0.16 | 0.91 |  |  | 3.62 | 0.06 | 0.53 |  |
| PRP | C-type lectin-like domain protein 9 | **13.47** | **0.00** | **0.02** | Y |  | 4.79 | 0.03 | 0.37 |  |
| PRP | C-type lectin-like domain protein 10 | 1.97 | 0.37 | 1.00 |  |  | 6.76 | 0.01 | 0.16 |  |
| PRP | C-type lectin-like domain protein 11 | **33.66** | **0.00** | **0.00** | Y |  | **28.82** | **0.00** | **0.00** | Y |
| PRP | C-type lectin-like domain protein 12 | **29.31** | **0.00** | **0.00** | A |  | 9.70 | 0.00 | 0.05 |  |
| PRP | C-type lectin-like domain protein 13 | **21.66** | **0.00** | **0.00** | Y |  | **12.44** | **0.00** | **0.01** | Y |
| PRP | C-type lectin-like domain protein 14 | 9.11 | 0.01 | 0.11 |  |  | 4.84 | 0.03 | 0.36 |  |
| PRP | C-type lectin-like domain protein 15 | **131.75** | **0.00** | **0.00** | Y |  | **77.99** | **0.00** | **0.00** | Y |
| PRP | C-type lectin-like domain protein 16 | **45.13** | **0.00** | **0.00** | M |  | **33.78** | **0.00** | **0.00** | Y |
| PRP | C-type lectin-like domain protein 17 | 4.78 | 0.09 | 0.65 |  |  | 0.67 | 0.41 | 1.00 |  |
| PRP & E | Gram-negative binding protein 1 | **344.83** | **0.00** | **0.00** | Y |  | **59.66** | **0.00** | **0.00** | Y |
| PRP & E | Gram-negative binding protein 2 | **17.35** | **0.00** | **0.00** | M |  | 3.06 | 0.08 | 0.69 |  |
| PRP & E | Gram-negative binding protein 3 | **57.42** | **0.00** | **0.00** | M |  | **24.64** | **0.00** | **0.00** | Y |
| PRP | Hemolymph lipopolysaccharide-binding protein 1 | 4.69 | 0.10 | 0.67 |  |  | **14.21** | **0.00** | **0.01** | Y |
| PRP | Hemolymph lipopolysaccharide-binding protein 2 | **46.38** | **0.00** | **0.00** | M |  | 4.18 | 0.04 | 0.47 |  |
| PRP | Hemolymph lipopolysaccharide-binding protein 3 | 2.97 | 0.23 | 1.00 |  |  | 1.36 | 0.24 | 1.00 |  |
| PRP | Hemolymph lipopolysaccharide-binding protein 4 | **48.49** | **0.00** | **0.00** | Y |  | **41.21** | **0.00** | **0.00** | Y |
| PRP | Hemolymph lipopolysaccharide-binding protein 5 | **64.11** | **0.00** | **0.00** | Y |  | **37.62** | **0.00** | **0.00** | Y |
| PRP | Hemolymph lipopolysaccharide-binding protein 6 | **131.75** | **0.00** | **0.00** | Y |  | **77.99** | **0.00** | **0.00** | Y |
| PRP | Laminin 1 | **21.86** | **0.00** | **0.00** | M |  | **11.93** | **0.00** | **0.02** | Y |
| PRP | Agglucetin 1 | 6.84 | 0.03 | 0.29 |  |  | 0.01 | 0.93 | 1.00 |  |
| PRP | Endo-beta-1,4-glucanase 1 | **502.14** | **0.00** | **0.00** | A |  | **10.74** | **0.00** | **0.03** | Y |
| PRP | Peptidoglycan recognition protein I-alpha | **109.00** | **0.00** | **0.00** | M |  | **78.50** | **0.00** | **0.00** | Y |
| PRP | Peptidoglycan recognition protein LB | **20.50** | **0.00** | **0.00** | M |  | 0.01 | 0.92 | 1.00 |  |
| PRP | Peptidoglycan recognition protein LE 1 | **169.00** | **0.00** | **0.00** | A |  | 0.69 | 0.41 | 1.00 |  |
| PRP | Peptidoglycan recognition protein SC 1 | **508.00** | **0.00** | **0.00** | A |  | **483.00** | **0.00** | **0.00** | A |
| PRP | Peptidoglycan recognition protein SC 2 | **11.50** | **0.00** | **0.04** | M |  | 1.11 | 0.29 | 1.00 |  |
| PRP | Peptidoglycan recognition protein SD | **29.90** | **0.00** | **0.00** | M |  | 2.31 | 0.13 | 0.96 |  |
| PRP | Peptidoglycan recognition protein | **19,80** | **0.00** | **0.00** | Y |  | **25.50** | **0.00** | **0.00** | Y |
| S | Serine protease 1 | 0.00 | 1.00 | 1.00 |  |  | 0.00 | 1.00 | 1.00 |  |
| S | Serine protease 2 | 7.24 | 0.03 | 0.24 |  |  | **16.31** | **0.00** | **0.00** | Y |
| S | Serine protease 3 | 10.97 | 0.00 | 0.05 |  |  | 5.78 | 0.02 | 0.24 |  |
| S | Serine protease 4 | **12.08** | **0.00** | **0.03** | Y |  | 0.34 | 0.56 | 1.00 |  |
| S | Serine protease 5 | **105.72** | **0.00** | **0.00** | Y |  | **110.13** | **0.00** | **0.00** | Y |
| S | Serine protease 6 | **27.90** | **0.00** | **0.00** | M |  | **34.06** | **0.00** | **0.00** | Y |
| S | Serine protease 7 | **16.83** | **0.00** | **0.00** | M |  | **19.41** | **0.00** | **0.00** | Y |
| S | Serine protease 8 | 10.64 | 0.00 | 0.06 |  |  | 0.89 | 0.34 | 1.00 |  |
| S | Serine protease 9 | **28.84** | **0.00** | **0.00** | Y |  | **19.28** | **0.00** | **0.00** | Y |
| S | Serine protease 10 | **679.66** | **0.00** | **0.00** | Y |  | **355.87** | **0.00** | **0.00** | Y |
| S | Serine protease 11 | **56.19** | **0.00** | **0.00** | M |  | 7.10 | 0.01 | 0.14 |  |
| S | Serine protease 12 | **60.52** | **0.00** | **0.00** | M |  | **81.97** | **0.00** | **0.00** | Y |
| S | Serine protease 13 | **674.86** | **0.00** | **0.00** | Y |  | **243.89** | **0.00** | **0.00** | Y |
| S | Serine protease 14 | 0.00 | 1.00 | 1.00 |  |  | **42.44** | **0.00** | **0.00** | A |
| S | Serine protease 15 | **17.23** | **0.00** | **0.00** | M |  | **23.07** | **0.00** | **0.00** | Y |
| S | Serine protease 16 | **17.45** | **0.00** | **0.00** | Y |  | 7.12 | 0.01 | 0.14 |  |
| S | Serine protease 17 | 8.36 | 0.02 | 0.15 |  |  | 4.86 | 0.03 | 0.36 |  |
| S | Serine protease 18 | **58.38** | **0.00** | **0.00** | M |  | **11.86** | **0.00** | **0.02** | Y |
| S | Serine protease 19 | **30.81** | **0.00** | **0.00** | M |  | **12.32** | **0.00** | **0.01** | Y |
| S | Serine protease 20 | **471.86** | **0.00** | **0.00** | M |  | **25.55** | **0.00** | **0.00** | Y |
| S | Serine protease 21 | **13.81** | **0.00** | **0.01** | A |  | 1.62 | 0.20 | 1.00 |  |
| S | Serine protease 22 | 6.91 | 0.03 | 0.28 |  |  | 0.92 | 0.34 | 1.00 |  |
| S | Serine protease 23 | **48.49** | **0.00** | **0.00** | Y |  | **47.99** | **0.00** | **0.00** | Y |
| S | Serine protease 24 | 4.72 | 0.09 | 0.66 |  |  | 0.19 | 0.66 | 1.00 |  |
| S | Serine protease 25 | **161.89** | **0.00** | **0.00** | Y |  | **26.91** | **0.00** | **0.00** | Y |
| S | Serine protease 26 | 8.00 | 0.02 | 0.18 |  |  | 9.65 | 0.00 | 0.05 |  |
| S | Serine protease 27 | **40.97** | **0.00** | **0.00** | Y |  | **33.42** | **0.00** | **0.00** | Y |
| S | Serine protease 28 | **25.03** | **0.00** | **0.00** | Y |  | **21.26** | **0.00** | **0.00** | Y |
| S | Serine protease 29 | 2.42 | 0.30 | 1.00 |  |  | 0.00 | 0.95 | 1.00 |  |
| S | Serine protease 30 | **42.95** | **0.00** | **0.00** | M |  | **28.79** | **0.00** | **0.00** | Y |
| S | Serine protease 31 | **68.87** | **0.00** | **0.00** | M |  | **39.62** | **0.00** | **0.00** | Y |
| S | Serine protease 32 | **74.02** | **0.00** | **0.00** | M |  | 0.32 | 0.57 | 1.00 |  |
| S | Serine protease 33 | **36.35** | **0.00** | **0.00** | M |  | **45.69** | **0.00** | **0.00** | Y |
| S | Serine protease 34 | **19.09** | **0.00** | **0.00** | M |  | **12.37** | **0.00** | **0.01** | Y |
| S | Serine protease 35 | **256.90** | **0.00** | **0.00** | M |  | **345.73** | **0.00** | **0.00** | Y |
| S | Serine protease 36 | **168.60** | **0.00** | **0.00** | Y |  | **114.15** | **0.00** | **0.00** | Y |
| S | Serine protease 37 | **20.45** | **0.00** | **0.00** | M |  | 2.40 | 0.12 | 0.92 |  |
| S | Serine protease 38 | **40.56** | **0.00** | **0.00** | Y |  | 0.01 | 0.93 | 1.00 |  |
| S | Serine protease 39 | **16.80** | **0.00** | **0.00** | M |  | 5.48 | 0.02 | 0.27 |  |
| S | Serine protease 40 | **12.37** | **0.00** | **0.03** | M |  | **13.43** | **0.00** | **0.01** | Y |
| S | Serine protease 41 | 5.26 | 0.07 | 0.55 |  |  | 3.05 | 0.08 | 0.69 |  |
| S | Serine protease 42 | **23.05** | **0.00** | **0.00** | Y |  | **22.07** | **0.00** | **0.00** | Y |
| S | Serine protease 43 | **18.75** | **0.00** | **0.00** | Y |  | 2.30 | 0.13 | 0.96 |  |
| S | Serine protease 44 | **135.63** | **0.00** | **0.00** | Y |  | **71.25** | **0.00** | **0.00** | Y |
| S | Serine protease 45 | **154.49** | **0.00** | **0.00** | Y |  | **61.13** | **0.00** | **0.00** | Y |
| S | Serine protease 46 | **29.19** | **0.00** | **0.00** | Y |  | 8.97 | 0.00 | 0.06 |  |
| S | Serine protease 47 | **18.59** | **0.00** | **0.00** | M |  | **14.86** | **0.00** | **0.00** | Y |
| S | Serine protease 48 | **187.05** | **0.00** | **0.00** | Y |  | **97.66** | **0.00** | **0.00** | Y |
| S | Serine protease 49 | **161.90** | **0.00** | **0.00** | Y |  | **55.07** | **0.00** | **0.00** | Y |
| S | Serine protease 50 | **27.64** | **0.00** | **0.00** | Y |  | 6.54 | 0.01 | 0.17 |  |
| S | Serine protease 51 | **32.25** | **0.00** | **0.00** | Y |  | **11.20** | **0.00** | **0.02** | Y |
| S | Serine protease 52 | **253.08** | **0.00** | **0.00** | Y |  | **220.10** | **0.00** | **0.00** | Y |
| S | Serine protease 53 | **69.80** | **0.00** | **0.00** | Y |  | **18.49** | **0.00** | **0.00** | Y |
| S | Serine protease 54 | **321.48** | **0.00** | **0.00** | Y |  | **51.25** | **0.00** | **0.00** | Y |
| S | Serine protease 55 | **106.12** | **0.00** | **0.00** | M |  | **51.60** | **0.00** | **0.00** | Y |
| S | Serine protease 56 | 7.39 | 0.02 | 0.23 |  |  | 7.67 | 0.01 | 0.11 |  |
| S | Serine protease 57 | **12.52** | **0.00** | **0.03** | Y |  | **10.98** | **0.00** | **0.03** | Y |
| S | Serine protease 58 | **28.45** | **0.00** | **0.00** | Y |  | 1.42 | 0.23 | 1.00 |  |
| S | Serine protease 59 | 5.92 | 0.05 | 0.42 |  |  | 1.54 | 0.21 | 1.00 |  |
| S | Serine protease 60 | **19.38** | **0.00** | **0.00** | M |  | 1.26 | 0.26 | 1.00 |  |
| S | Serine protease 61 | **14.86** | **0.00** | **0.01** | Y |  | 0.81 | 0.37 | 1.00 |  |
| S | Serine protease 62 | **146.77** | **0.00** | **0.00** | M |  | **72.87** | **0.00** | **0.00** | A |
| S | Serine protease 63 | 0.59 | 0.74 | 1.00 |  |  | **49.12** | **0.00** | **0.00** | A |
| S | Serine protease 64 | **129.38** | **0.00** | **0.00** | Y |  | **90.34** | **0.00** | **0.00** | Y |
| S | Serine protease 65 | **43.03** | **0.00** | **0.00** | Y |  | **34.29** | **0.00** | **0.00** | Y |
| S | Serine protease 66 | **82.41** | **0.00** | **0.00** | Y |  | 3.07 | 0.08 | 0.68 |  |
| S | Serine protease 67 | 4.17 | 0.12 | 0.79 |  |  | 7.47 | 0.01 | 0.12 |  |
| S | Serine protease 68 | **45.13** | **0.00** | **0.00** | M |  | **33.78** | **0.00** | **0.00** | Y |
| S | Serine protease 69 | **12.47** | **0.00** | **0.03** | Y |  | 6.65 | 0.01 | 0.17 |  |
| S | Serine protease 70 | **39.77** | **0.00** | **0.00** | Y |  | 9.69 | 0.00 | 0.05 |  |
| S | Kazal-type serine protease inhibitor domain-containing protein 1 | 6.88 | 0.03 | 0.29 |  |  | **17.53** | **0.00** | **0.00** | Y |
| S | Serine protease inhibitor 1 | **68.87** | **0.00** | **0.00** | M |  | **39.62** | **0.00** | **0.00** | Y |
| S | Serine protease inhibitor 2 | 4.65 | 0.10 | 0.68 |  |  | 0.28 | 0.60 | 1.00 |  |
| S | Serine protease inhibitor 3 | **69.80** | **0.00** | **0.00** | Y |  | **18.49** | **0.00** | **0.00** | Y |
| S | Serine protease inhibitor 4 | **43.03** | **0.00** | **0.00** | Y |  | **34.29** | **0.00** | **0.00** | Y |
| S | Serine protease inhibitor dipetalogastin 1 | **68.57** | **0.00** | **0.00** | Y |  | **16.09** | **0.00** | **0.00** | Y |
| S | Serine protease inhibitor dipetalogastin 2 | **31.66** | **0.00** | **0.00** | A, M |  | 4.92 | 0.03 | 0.35 |  |
| S | Prophenoloxidase activating factor 1 | **139.28** | **0.00** | **0.00** | Y |  | **102.21** | **0.00** | **0.00** | Y |
| S | 14-3-3 protein 1 | **56.92** | **0.00** | **0.00** | Y |  | **44.14** | **0.00** | **0.00** | Y |
| S | 14-3-3 protein 2 | **36.81** | **0.00** | **0.00** | Y |  | **24.86** | **0.00** | **0.00** | Y |
| S | 14-3-3 protein 3 | **54.26** | **0.00** | **0.00** | Y |  | 0.06 | 0.80 | 1.00 |  |
| S | Calpain 1 | **35.67** | **0.00** | **0.00** | Y |  | **12.64** | **0.00** | **0.01** | Y |
| S | Calpain 2 | 10.91 | 0.00 | 0.05 |  |  | **29.55** | **0.00** | **0.00** | Y |
| S | Calpain 3 | 5.34 | 0.07 | 0.53 |  |  | 2.46 | 0.12 | 0.90 |  |
| S | Calpain 4 | **24.13** | **0.00** | **0.00** | M |  | 4.37 | 0.04 | 0.43 |  |
| S | Calpain 5 | **37.01** | **0.00** | **0.00** | M |  | 5.60 | 0.02 | 0.26 |  |
| S | Minor histocompatibility antigen 1 | 8.48 | 0.01 | 0.15 |  |  | **24.67** | **0.00** | **0.00** | Y |
| S | Minor histocompatibility antigen 2 | **25.79** | **0.00** | **0.00** | M |  | **13.87** | **0.00** | **0.01** | Y |
| S | Low-density lipoprotein receptor-related protein 1 | **25.50** | **0.00** | **0.00** | M |  | **16.83** | **0.00** | **0.00** | M |
| S | Low-density lipoprotein receptor-related protein 2 | **25.36** | **0.00** | **0.00** | M |  | 0.63 | 0.43 | 1.00 |  |
| S | Low-density lipoprotein receptor-related protein 3 | **22.88** | **0.00** | **0.00** | Y |  | 4.56 | 0.03 | 0.41 |  |
| S | Low-density lipoprotein receptor-related protein 4 | 9.27 | 0.01 | 0.11 |  |  | **11.39** | **0.00** | **0.02** | Y |
| S | Low-density lipoprotein receptor-related protein 5 | 4.60 | 0.10 | 0.70 |  |  | 1.61 | 0.20 | 1.00 |  |
| S | Low-density lipoprotein receptor-related protein 6 | **58.34** | **0.00** | **0.00** | Y |  | 6.92 | 0.01 | 0.15 |  |
| S | Low-density lipoprotein receptor-related protein 7 | **30.93** | **0.00** | **0.00** | Y |  | **66.06** | **0.00** | **0.00** | Y |
| S | Low-density lipoprotein receptor-related protein 8 | **12.74** | **0.00** | **0.02** | A |  | 4.75 | 0.03 | 0.37 |  |
| S | Four and a half LIM domains protein 1 | **21.01** | **0.00** | **0.00** | Y |  | 3.94 | 0.05 | 0.52 |  |
| E | Carboxypeptidase 1 | **498.39** | **0.00** | **0.00** | Y |  | **293.03** | **0.00** | **0.00** | Y |
| E | Carboxypeptidase 2 | **36.84** | **0.00** | **0.00** | Y |  | **28.11** | **0.00** | **0.00** | Y |
| E | Carboxypeptidase 3 | **519.68** | **0.00** | **0.00** | Y |  | **338.47** | **0.00** | **0.00** | Y |
| E | Carboxypeptidase 4 | **325.33** | **0.00** | **0.00** | Y |  | **341.39** | **0.00** | **0.00** | Y |
| E | Carboxypeptidase 5 | **21.06** | **0.00** | **0.00** | M |  | 1.52 | 0.22 | 1.00 |  |
| E | Carboxypeptidase 6 | **459.30** | **0.00** | **0.00** | Y |  | **364.49** | **0.00** | **0.00** | Y |
| E | Carboxypeptidase 7 | **32.57** | **0.00** | **0.00** | M |  | 6.72 | 0.01 | 0.16 |  |
| E | Carboxypeptidase 8 | **36.53** | **0.00** | **0.00** | M |  | **37.68** | **0.00** | **0.00** | Y |
| E | Carboxypeptidase 9 | **116.48** | **0.00** | **0.00** | A, M |  | **73.92** | **0.00** | **0.00** | A |
| E | Carboxypeptidase 10 | **26.20** | **0.00** | **0.00** | Y |  | 4.20 | 0.04 | 0.46 |  |
| E | Carboxypeptidase 11 | **20.49** | **0.00** | **0.00** | Y |  | **24.43** | **0.00** | **0.00** | Y |
| E | Cathepsin 1 | 0.00 | 1.00 | 1.00 |  |  | 0.00 | 1.00 | 1.00 |  |
| E | Cathepsin 2 | 0.00 | 1.00 | 1.00 |  |  | 0.00 | 1.00 | 1.00 |  |
| E | Cathepsin 3 | 8.99 | 0.01 | 0.12 |  |  | 0.58 | 0.45 | 1.00 |  |
| E | Cathepsin 4 | **38.93** | **0.00** | **0.00** | M |  | **26.28** | **0.00** | **0.00** | Y |
| E | Cathepsin 5 | 3.79 | 0.15 | 0.90 |  |  | 3.86 | 0.05 | 0.53 |  |
| E | Cathepsin 6 | **19.76** | **0.00** | **0.00** | M |  | **18.15** | **0.00** | **0.00** | Y |
| E | Cathepsin 7 | **12.37** | **0.00** | **0.03** | M |  | **13.43** | **0.00** | **0.01** | Y |
| E | Cathepsin 8 | **25.91** | **0.00** | **0.00** | Y |  | **21.93** | **0.00** | **0.00** | Y |
| E | Cathepsin 9 | **29.32** | **0.00** | **0.00** | M |  | **30.53** | **0.00** | **0.00** | Y |
| E | Cathepsin 10 | **54.49** | **0.00** | **0.00** | Y |  | 7.34 | 0.01 | 0.12 |  |
| E | Lysozyme C type 1 | **412.57** | **0.00** | **0.00** | M |  | **52.78** | **0.00** | **0.00** | Y |
| E | Lysozyme-like protein | 1.36 | 0.51 | 1.00 |  |  | 0.27 | 0.61 | 1.00 |  |
| E | Lysozyme P type | **382.85** | **0.00** | **0.00** | M |  | **196.70** | **0.00** | **0.00** | A |
| E | Lysozyme C type 2 | **504.35** | **0.00** | **0.00** | M |  | **235.63** | **0.00** | **0.00** | A |
| E | Lysozyme C type-like protein | **21.28** | **0.00** | **0.00** | M |  | 6.14 | 0.01 | 0.21 |  |
| E | Lysozyme I type 1 | **15.33** | **0.00** | **0.01** | Y |  | 8.26 | 0.00 | 0.08 |  |
| E | Lysozyme I type 2 | **18.61** | **0.00** | **0.00** | M |  | 0.53 | 0.47 | 1.00 |  |
| E | Lysozyme I type 3 | **46.13** | **0.00** | **0.00** | Y |  | **26.13** | **0.00** | **0.00** | Y |
| E | Lysozyme C type 3 | **246.66** | **0.00** | **0.00** | A |  | **31.39** | **0.00** | **0.00** | A |
| E | Metacaspase-like cysteine peptidase 1 | **18.99** | **0.00** | **0.00** | Y |  | **12.54** | **0.00** | **0.01** | Y |
| E | Metacaspase-like cysteine peptidase 2 | **13.22** | **0.00** | **0.02** | A |  | **36.00** | **0.00** | **0.00** | A |
| E | Asparaginyl endopeptidase-like cysteine peptidase 1 | 8.50 | 0.01 | 0.14 |  |  | **21.17** | **0.00** | **0.00** | A |
| E | Lysosomal Pro-X carboxypeptidase 1 | 8.94 | 0.01 | 0.12 |  |  | **10.64** | **0.00** | **0.03** | Y |
| E | Prolixicin antimicrobial protein 1 | **22.68** | **0.00** | **0.00** | M |  | 2.26 | 0.13 | 0.98 |  |
| E | Transferrin 1 | **58.64** | **0.00** | **0.00** | M |  | **65.24** | **0.00** | **0.00** | Y |
| E | Transferrin 2 | **22.54** | **0.00** | **0.00** | M |  | 3.22 | 0.07 | 0.64 |  |
| E | Transferrin 3 | 8.36 | 0.02 | 0.15 |  |  | **12.25** | **0.00** | **0.01** | Y |
| E | Termicin 1 | **125.03** | **0.00** | **0.00** | M |  | **31.97** | **0.00** | **0.00** | Y |
| E | Cysteine-rich protein 1 | **17.85** | **0.00** | **0.00** | M |  | 0.00 | 0.99 | 1.00 |  |
| E | Cysteine-rich protein 2 | 0.55 | 0.76 | 1.00 |  |  | 1.04 | 0.31 | 1.00 |  |
| E | Cysteine-rich protein 3 | **28.22** | **0.00** | **0.00** | M |  | **15.92** | **0.00** | **0.00** | Y |
| E | Cysteine-rich protein 4 | 2.90 | 0.23 | 1.00 |  |  | 0.11 | 0.74 | 1.00 |  |
| E | Cysteine-rich protein 5 | **89.15** | **0.00** | **0.00** | M |  | 0.80 | 0.37 | 1.00 |  |
| E | Cysteine-rich protein 6 | **116.19** | **0.00** | **0.00** | A |  | **91.76** | **0.00** | **0.00** | A |
| E | Cysteine-rich protein 7 | **12.93** | **0.00** | **0.02** | M |  | 5.26 | 0.02 | 0.30 |  |
| E | Cysteine-rich protein 8 | 0.90 | 0.64 | 1.00 |  |  | 1.51 | 0.22 | 1.00 |  |
| E | Cysteine-rich protein 9 | **16.32** | **0.00** | **0.00** | Y |  | 5.05 | 0.02 | 0.33 |  |
| E | Cysteine-rich protein 10 | **30.97** | **0.00** | **0.00** | Y |  | **20.95** | **0.00** | **0.00** | Y |
| E | Cysteine-rich protein 11 | **11.98** | **0.00** | **0.03** | M |  | 6.64 | 0.01 | 0.17 |  |
| E | Cysteine-rich protein 12 | **22.16** | **0.00** | **0.00** | A |  | 0.64 | 0.43 | 1.00 |  |
| E | Cysteine-rich protein 13 | **116.19** | **0.00** | **0.00** | A |  | **91.76** | **0.00** | **0.00** | A |
| E | Cysteine-rich protein 14 | **13.81** | **0.00** | **0.01** | M |  | **35.20** | **0.00** | **0.00** | A |
| E | Ferritin 1 | 0.00 | 1.00 | 1.00 |  |  | 0.00 | 1.00 | 1.00 |  |
| E | Ferritin 2 | 10.36 | 0.01 | 0.07 |  |  | 2.89 | 0.09 | 0.74 |  |
| E | Ferritin 3 | 7.13 | 0.03 | 0.26 |  |  | **14.72** | **0.00** | **0.00** | Y |
| E | Ferritin 4 | 6.80 | 0.03 | 0.29 |  |  | **13.44** | **0.00** | **0.01** | Y |
| E | Melanotransferrin 1 | **40.38** | **0.00** | **0.00** | Y |  | **24.67** | **0.00** | **0.00** | Y |
| E | Venom allergen 1 | **32.59** | **0.00** | **0.00** |  |  | **9.82** | **0.00** | **0.04** | Y |
| E | Thaumatin-like protein 1 | 2.27 | 0.32 | 1.00 |  |  | 0.11 | 0.74 | 1.00 |  |

Comparison of normalized counts per million (CPM) among male reproductives (alates (A), young primary kings (Y) and mature primary kings (M)) or female ones (alates (A) and young primary queens (Y)) was conducted using edgeR package. Bold letters mean significant difference (FDR < 0.05). “Caste showing the highest expression” means the caste showing the largest mean CPM among all age classes for each gene, and the largest mean CPM in a certain age class is more than 1.2 times as high as the mean CPM in the other classes. LR: likelihood ratio, FDR: false discovery rate, PRP: pattern recognition protein, S: signalling protein, E: Effector.
