# Supplementary material for: Caste-, sex-, and age-dependent expression of immune-related genes in a Japanese subterranean termite, Reticulitermes speratus
Source: PLoS One. 2017 Apr 14;12(4):e0175417. doi: 10.1371/journal.pone.0175417 (PMC5391962; doi:10.1371/journal.pone.0175417)
Supplement: S5 Table — Comparison of normalized counts per million (CPM) between reproductive statuses (reproductive castes: alates, young PKs and PQs, and mature PKs and SQs; neuter castes: soldiers and workers) and between sexes was conducted by edgeR package. Bold letters mean significant differences (FDR < 0.05). LR: likelihood ratio, FDR: false discovery rate, PRP: pattern recognition protein, S: Signalling protein, E: effector. (DOCX) [file pone.0175417.s009.docx]

**Table S5. Statistical results of differential expression between reproductive and neuter castes and between males and females.**

| Functional category | Gene name | Reproductive status | | |  | Sex differences nested by reproductive statuses | | |
| --- | --- | --- | --- | --- | --- | --- | --- | --- |
|  |  | LR | P-Value | FDR |  | LR | P-Value | FDR |
| PRP | Apolipophorin1 | 28.35 | 0.00 | **0.00** |  | 10.60 | 0.01 | 0.07 |
| PRP | Apolipophorin2 | 4.16 | 0.04 | 0.36 |  | 1.36 | 0.51 | 1.00 |
| PRP | Apolipophorin3 | 25.70 | 0.00 | **0.00** |  | 1.82 | 0.40 | 1.00 |
| PRP | Brevican1 | 9.60 | 0.00 | **0.03** |  | 14.48 | 0.00 | **0.01** |
| PRP | C-type lectin-like domain protein1 | 1.31 | 0.25 | 1.00 |  | 6.71 | 0.03 | 0.32 |
| PRP | C-type lectin-like domain protein2 | 1.47 | 0.23 | 1.00 |  | 16.05 | 0.00 | **0.01** |
| PRP | C-type lectin-like domain protein3 | 14.47 | 0.00 | **0.00** |  | 20.90 | 0.00 | **0.00** |
| PRP | C-type lectin-like domain protein4 | 3.47 | 0.06 | 0.49 |  | 73.67 | 0.00 | **0.00** |
| PRP | C-type lectin-like domain protein5 | 8.90 | 0.00 | 0.05 |  | 0.54 | 0.76 | 1.00 |
| PRP | C-type lectin-like domain protein6 | 7.99 | 0.00 | 0.07 |  | 16.08 | 0.00 | **0.01** |
| PRP | C-type lectin-like domain protein7 | 116.79 | 0.00 | **0.00** |  | 24.22 | 0.00 | **0.00** |
| PRP | C-type lectin-like domain protein8 | 0.48 | 0.49 | 1.00 |  | 0.60 | 0.74 | 1.00 |
| PRP | C-type lectin-like domain protein9 | 16.02 | 0.00 | **0.00** |  | 35.87 | 0.00 | **0.00** |
| PRP | C-type lectin-like domain protein10 | 10.88 | 0.00 | **0.02** |  | 30.73 | 0.00 | **0.00** |
| PRP | C-type lectin-like domain protein11 | 77.66 | 0.00 | **0.00** |  | 10.23 | 0.01 | 0.08 |
| PRP | C-type lectin-like domain protein12 | 106.33 | 0.00 | **0.00** |  | 2.34 | 0.31 | 1.00 |
| PRP | C-type lectin-like domain protein13 | 160.25 | 0.00 | **0.00** |  | 1.18 | 0.56 | 1.00 |
| PRP | C-type lectin-like domain protein14 | 18.10 | 0.00 | **0.00** |  | 27.39 | 0.00 | **0.00** |
| PRP | C-type lectin-like domain protein15 | 172.66 | 0.00 | **0.00** |  | 3.71 | 0.16 | 0.91 |
| PRP | C-type lectin-like domain protein16 | 0.01 | 0.90 | 1.00 |  | 0.88 | 0.65 | 1.00 |
| PRP | C-type lectin-like domain protein17 | 87.66 | 0.00 | **0.00** |  | 118.95 | 0.00 | **0.00** |
| PRP & E | Gram-negative bacteria binding protein1 | 25.26 | 0.00 | **0.00** |  | 13.36 | 0.00 | **0.02** |
| PRP & E | Gram-negative bacteria binding protein2 | 0.22 | 0.64 | 1.00 |  | 36.15 | 0.00 | **0.00** |
| PRP & E | Gram-negative bacteria binding protein3 | 25.05 | 0.00 | **0.00** |  | 1.58 | 0.45 | 1.00 |
| PRP | Lipopolysaccharide-binding protein1 | 4.47 | 0.03 | 0.31 |  | 4.09 | 0.13 | 0.81 |
| PRP | Lipopolysaccharide-binding protein2 | 47.98 | 0.00 | **0.00** |  | 28.46 | 0.00 | **0.00** |
| PRP | Lipopolysaccharide-binding protein3 | 42.12 | 0.00 | **0.00** |  | 8.87 | 0.01 | 0.14 |
| PRP | Lipopolysaccharide-binding protein4 | 15.70 | 0.00 | **0.00** |  | 3.61 | 0.16 | 0.93 |
| PRP | Lipopolysaccharide-binding protein5 | 24.92 | 0.00 | **0.00** |  | 8.93 | 0.01 | 0.14 |
| PRP | Lipopolysaccharide-binding protein6 | 172.66 | 0.00 | **0.00** |  | 3.71 | 0.16 | 0.91 |
| PRP | Laminin1 | 0.48 | 0.49 | 1.00 |  | 25.01 | 0.00 | **0.00** |
| PRP | Agglucetin1 | 0.44 | 0.51 | 1.00 |  | 4.38 | 0.11 | 0.74 |
| PRP | Endo-beta-1,4-glucanase1 | 18.26 | 0.00 | **0.00** |  | **23.77** | **0.00** | **0.00** |
| PRP | Peptidoglycan recognition protein I-alpha | 0.30 | 0.58 | 1.00 |  | **12.70** | **0.00** | **0.03** |
| PRP | Peptidoglycan recognition protein LB | **221.00** | **0.00** | **0.00** |  | 4.48 | 0.11 | 0.71 |
| PRP | Peptidoglycan recognition protein LE 1 | **66.00** | **0.00** | **0.00** |  | **17.80** | **0.00** | **0.00** |
| PRP | Peptidoglycan recognition protein SC 1 | **587.00** | **0.00** | **0.00** |  | **18.40** | **0.00** | **0.00** |
| PRP | Peptidoglycan recognition protein SC 2 | 0.78 | 0.38 | 1.00 |  | **18.60** | **0.00** | **0.00** |
| PRP | Peptidoglycan recognition protein SD | 0.04 | 0.85 | 1.00 |  | 1.38 | 0.50 | 1.00 |
| PRP | Peptidoglycan recognition protein | 0.00 | 0.00 | 0.06 |  | 1.47 | 0.48 | 1.00 |
| S | Serine protease 1 | 0.00 | 1.00 | 1.00 |  | 48.23 | 0.00 | **0.00** |
| S | Serine protease 2 | 0.22 | 0.64 | 1.00 |  | 0.82 | 0.66 | 1.00 |
| S | Serine protease 3 | 29.29 | 0.00 | **0.00** |  | 101.24 | 0.00 | **0.00** |
| S | Serine protease 4 | 6.29 | 0.01 | 0.14 |  | 111.65 | 0.00 | **0.00** |
| S | Serine protease 5 | 31.72 | 0.00 | **0.00** |  | 1.79 | 0.41 | 1.00 |
| S | Serine protease 6 | 3.46 | 0.06 | 0.49 |  | 2.37 | 0.31 | 1.00 |
| S | Serine protease 7 | 167.03 | 0.00 | **0.00** |  | 0.19 | 0.91 | 1.00 |
| S | Serine protease 8 | 58.85 | 0.00 | **0.00** |  | 6.45 | 0.04 | 0.35 |
| S | Serine protease 9 | 0.31 | 0.58 | 1.00 |  | 8.92 | 0.01 | 0.14 |
| S | Serine protease 10 | 54.62 | 0.00 | **0.00** |  | 11.80 | 0.00 | **0.04** |
| S | Serine protease 11 | 3.96 | 0.05 | 0.40 |  | 12.00 | 0.00 | **0.04** |
| S | Serine protease 12 | 0.04 | 0.85 | 1.00 |  | 8.05 | 0.02 | 0.20 |
| S | Serine protease 13 | 72.31 | 0.00 | **0.00** |  | 4.64 | 0.10 | 0.68 |
| S | Serine protease 14 | 3.66 | 0.06 | 0.45 |  | 29.66 | 0.00 | **0.00** |
| S | Serine protease 15 | 18.49 | 0.00 | **0.00** |  | 4.15 | 0.13 | 0.80 |
| S | Serine protease 16 | 7.84 | 0.01 | 0.07 |  | 0.96 | 0.62 | 1.00 |
| S | Serine protease 17 | 0.10 | 0.75 | 1.00 |  | 5.01 | 0.08 | 0.60 |
| S | Serine protease 18 | 123.61 | 0.00 | **0.00** |  | 193.76 | 0.00 | **0.00** |
| S | Serine protease 19 | 3.98 | 0.05 | 0.40 |  | 3.71 | 0.16 | 0.91 |
| S | Serine protease 20 | 1331.84 | 0.00 | **0.00** |  | 218.97 | 0.00 | **0.00** |
| S | Serine protease 21 | 0.05 | 0.82 | 1.00 |  | 6.15 | 0.05 | 0.39 |
| S | Serine protease 22 | 5.88 | 0.02 | 0.17 |  | 0.45 | 0.80 | 1.00 |
| S | Serine protease 23 | 21.69 | 0.00 | **0.00** |  | 11.23 | 0.00 | 0.06 |
| S | Serine protease 24 | 0.60 | 0.44 | 1.00 |  | 1.40 | 0.50 | 1.00 |
| S | Serine protease 25 | 203.82 | 0.00 | **0.00** |  | 3.72 | 0.16 | 0.91 |
| S | Serine protease 26 | 36.89 | 0.00 | **0.00** |  | 7.02 | 0.03 | 0.28 |
| S | Serine protease 27 | 10.04 | 0.00 | **0.03** |  | 14.20 | 0.00 | **0.02** |
| S | Serine protease 28 | 83.90 | 0.00 | **0.00** |  | 12.58 | 0.00 | **0.03** |
| S | Serine protease 29 | 1.96 | 0.16 | 0.82 |  | 8.08 | 0.02 | 0.19 |
| S | Serine protease 30 | 57.42 | 0.00 | **0.00** |  | 2.20 | 0.33 | 1.00 |
| S | Serine protease 31 | 12.91 | 0.00 | **0.01** |  | 0.98 | 0.61 | 1.00 |
| S | Serine protease 32 | 5.26 | 0.02 | 0.23 |  | 11.65 | 0.00 | 0.05 |
| S | Serine protease 33 | 10.23 | 0.00 | **0.02** |  | 8.22 | 0.02 | 0.18 |
| S | Serine protease 34 | 84.87 | 0.00 | **0.00** |  | 5.67 | 0.06 | 0.47 |
| S | Serine protease 35 | 10.73 | 0.00 | **0.02** |  | 8.80 | 0.01 | 0.15 |
| S | Serine protease 36 | 0.69 | 0.41 | 1.00 |  | 5.09 | 0.08 | 0.58 |
| S | Serine protease 37 | 77.93 | 0.00 | **0.00** |  | 17.80 | 0.00 | **0.00** |
| S | Serine protease 38 | 35.25 | 0.00 | **0.00** |  | 3.47 | 0.18 | 0.96 |
| S | Serine protease 39 | 7.64 | 0.01 | 0.08 |  | 2.04 | 0.36 | 1.00 |
| S | Serine protease 40 | 6.94 | 0.01 | 0.11 |  | 4.65 | 0.10 | 0.68 |
| S | Serine protease 41 | 9.53 | 0.00 | **0.03** |  | 231.40 | 0.00 | **0.00** |
| S | Serine protease 42 | 9.29 | 0.00 | **0.04** |  | 2.04 | 0.36 | 1.00 |
| S | Serine protease 43 | 19.06 | 0.00 | **0.00** |  | 3.88 | 0.14 | 0.87 |
| S | Serine protease 44 | 55.81 | 0.00 | **0.00** |  | 30.82 | 0.00 | **0.00** |
| S | Serine protease 45 | 0.10 | 0.75 | 1.00 |  | 12.02 | 0.00 | **0.04** |
| S | Serine protease 46 | 36.02 | 0.00 | **0.00** |  | 69.76 | 0.00 | **0.00** |
| S | Serine protease 47 | 4.17 | 0.04 | 0.36 |  | 5.18 | 0.07 | 0.56 |
| S | Serine protease 48 | 10.57 | 0.00 | **0.02** |  | 15.33 | 0.00 | **0.01** |
| S | Serine protease 49 | 319.62 | 0.00 | **0.00** |  | 1.24 | 0.54 | 1.00 |
| S | Serine protease 50 | 0.80 | 0.37 | 1.00 |  | 7.86 | 0.02 | 0.21 |
| S | Serine protease 51 | 0.72 | 0.40 | 1.00 |  | 2.61 | 0.27 | 1.00 |
| S | Serine protease 52 | 12.76 | 0.00 | **0.01** |  | 3.37 | 0.19 | 0.99 |
| S | Serine protease 53 | 6.64 | 0.01 | 0.12 |  | 4.68 | 0.10 | 0.67 |
| S | Serine protease 54 | 44.09 | 0.00 | **0.00** |  | 3.41 | 0.18 | 0.98 |
| S | Serine protease 55 | 182.77 | 0.00 | **0.00** |  | 0.59 | 0.74 | 1.00 |
| S | Serine protease 56 | 18.57 | 0.00 | **0.00** |  | 4.01 | 0.13 | 0.83 |
| S | Serine protease 57 | 92.73 | 0.00 | **0.00** |  | 1.16 | 0.56 | 1.00 |
| S | Serine protease 58 | 7.59 | 0.01 | 0.08 |  | 56.44 | 0.00 | **0.00** |
| S | Serine protease 59 | 4.97 | 0.03 | 0.25 |  | 2.71 | 0.26 | 1.00 |
| S | Serine protease 60 | 0.43 | 0.51 | 1.00 |  | 17.02 | 0.00 | **0.01** |
| S | Serine protease 61 | 7.18 | 0.01 | 0.10 |  | 0.84 | 0.66 | 1.00 |
| S | Serine protease 62 | 234.50 | 0.00 | **0.00** |  | 347.08 | 0.00 | **0.00** |
| S | Serine protease 63 | 47.46 | 0.00 | **0.00** |  | 23.48 | 0.00 | **0.00** |
| S | Serine protease 64 | 14.57 | 0.00 | **0.00** |  | 5.48 | 0.06 | 0.50 |
| S | Serine protease 65 | 12.01 | 0.00 | **0.01** |  | 1.39 | 0.50 | 1.00 |
| S | Serine protease 66 | 6.49 | 0.01 | 0.13 |  | 1697.69 | 0.00 | **0.00** |
| S | Serine protease 67 | 0.77 | 0.38 | 1.00 |  | 1.05 | 0.59 | 1.00 |
| S | Serine protease 68 | 0.01 | 0.90 | 1.00 |  | 0.88 | 0.65 | 1.00 |
| S | Serine protease 69 | 104.01 | 0.00 | **0.00** |  | 22.00 | 0.00 | **0.00** |
| S | Serine protease 70 | 8.31 | 0.00 | 0.06 |  | 14.32 | 0.00 | **0.02** |
| S | Kazal-type serine protease inhibitor domain-containing protein 1 | 22.63 | 0.00 | **0.00** |  | 2.29 | 0.32 | 1.00 |
| S | Serine protease inhibitor 1 | 12.91 | 0.00 | **0.01** |  | 0.98 | 0.61 | 1.00 |
| S | Serine protease inhibitor 2 | 0.82 | 0.36 | 1.00 |  | 6.03 | 0.05 | 0.41 |
| S | Serine protease inhibitor 3 | 6.64 | 0.01 | 0.12 |  | 4.68 | 0.10 | 0.67 |
| S | Serine protease inhibitor 4 | 12.01 | 0.00 | **0.01** |  | 1.39 | 0.50 | 1.00 |
| S | Serine protease inhibitor dipetalogastin 1 | 0.49 | 0.48 | 1.00 |  | 23.64 | 0.00 | **0.00** |
| S | Serine protease inhibitor dipetalogastin 2 | 13.95 | 0.00 | **0.00** |  | 2.15 | 0.34 | 1.00 |
| S | Prophenoloxidase activating factor1 | 11.28 | 0.00 | **0.02** |  | 8.29 | 0.02 | 0.18 |
| S | 14-3-3 protein1 | 0.62 | 0.43 | 1.00 |  | 5.13 | 0.08 | 0.58 |
| S | 14-3-3 protein2 | 16.63 | 0.00 | **0.00** |  | 5.39 | 0.07 | 0.52 |
| S | 14-3-3 protein3 | 0.15 | 0.70 | 1.00 |  | 3.98 | 0.14 | 0.84 |
| S | Calpain1 | 0.36 | 0.55 | 1.00 |  | 0.79 | 0.68 | 1.00 |
| S | Calpain2 | 23.85 | 0.00 | **0.00** |  | 1.61 | 0.45 | 1.00 |
| S | Calpain3 | 0.35 | 0.56 | 1.00 |  | 72.76 | 0.00 | **0.00** |
| S | Calpain4 | 36.74 | 0.00 | **0.00** |  | 8.03 | 0.02 | 0.20 |
| S | Calpain5 | 2.95 | 0.09 | 0.56 |  | 3.40 | 0.18 | 0.98 |
| S | Minor histocompatibility protein1 | 2.98 | 0.08 | 0.56 |  | 5.37 | 0.07 | 0.52 |
| S | Minor histocompatibility protein2 | 23.11 | 0.00 | **0.00** |  | 11.50 | 0.00 | 0.05 |
| S | Low-density lipoprotein receptor-related protein1 | 0.01 | 0.94 | 1.00 |  | 3.83 | 0.15 | 0.88 |
| S | Low-density lipoprotein receptor-related protein2 | 1.52 | 0.22 | 1.00 |  | 6.65 | 0.04 | 0.33 |
| S | Low-density lipoprotein receptor-related protein3 | 171.79 | 0.00 | **0.00** |  | 393.61 | 0.00 | **0.00** |
| S | Low-density lipoprotein receptor-related protein4 | 10.13 | 0.00 | **0.03** |  | 13.19 | 0.00 | **0.03** |
| S | Low-density lipoprotein receptor-related protein5 | 18.21 | 0.00 | **0.00** |  | 34.20 | 0.00 | **0.00** |
| S | Low-density lipoprotein receptor-related protein6 | 20.49 | 0.00 | **0.00** |  | 193.13 | 0.00 | **0.00** |
| S | Low-density lipoprotein receptor-related protein7 | 62.54 | 0.00 | **0.00** |  | 9.93 | 0.01 | 0.09 |
| S | Low-density lipoprotein receptor-related protein8 | 8.51 | 0.00 | 0.05 |  | 0.96 | 0.62 | 1.00 |
| S | Four and a half LIM domains protein1 | 62.27 | 0.00 | **0.00** |  | 7.86 | 0.02 | 0.21 |
| E | Carboxypeptidase1 | 16.35 | 0.00 | **0.00** |  | 1.03 | 0.60 | 1.00 |
| E | Carboxypeptidase2 | 62.42 | 0.00 | **0.00** |  | 0.77 | 0.68 | 1.00 |
| E | Carboxypeptidase3 | 32.19 | 0.00 | **0.00** |  | 3.11 | 0.21 | 1.00 |
| E | Carboxypeptidase4 | 1.80 | 0.18 | 0.89 |  | 2.33 | 0.31 | 1.00 |
| E | Carboxypeptidase5 | 3.29 | 0.07 | 0.53 |  | 3.69 | 0.16 | 0.91 |
| E | Carboxypeptidase6 | 15.48 | 0.00 | **0.00** |  | 0.62 | 0.74 | 1.00 |
| E | Carboxypeptidase7 | 1.41 | 0.23 | 1.00 |  | 8.37 | 0.02 | 0.17 |
| E | Carboxypeptidase8 | 8.79 | 0.00 | 0.05 |  | 5.95 | 0.05 | 0.42 |
| E | Carboxypeptidase9 | 2.61 | 0.11 | 0.61 |  | 5.99 | 0.05 | 0.41 |
| E | Carboxypeptidase10 | 44.41 | 0.00 | **0.00** |  | 7.90 | 0.02 | 0.21 |
| E | Carboxypeptidase11 | 3.83 | 0.05 | 0.42 |  | 1.50 | 0.47 | 1.00 |
| E | Cathepsin1 | 0.00 | 1.00 | 1.00 |  | 25.26 | 0.00 | **0.00** |
| E | Cathepsin2 | 0.00 | 1.00 | 1.00 |  | 74.15 | 0.00 | **0.00** |
| E | Cathepsin3 | 1.28 | 0.26 | 1.00 |  | 9.78 | 0.01 | 0.10 |
| E | Cathepsin4 | 0.09 | 0.77 | 1.00 |  | 3.36 | 0.19 | 0.99 |
| E | Cathepsin5 | 1.73 | 0.19 | 0.92 |  | 4.96 | 0.08 | 0.61 |
| E | Cathepsin6 | 0.03 | 0.86 | 1.00 |  | 0.52 | 0.77 | 1.00 |
| E | Cathepsin7 | 6.94 | 0.01 | 0.11 |  | 4.65 | 0.10 | 0.68 |
| E | Cathepsin8 | 2.86 | 0.09 | 0.56 |  | 1.48 | 0.48 | 1.00 |
| E | Cathepsin9 | 0.76 | 0.38 | 1.00 |  | 1.48 | 0.48 | 1.00 |
| E | Cathepsin10 | 17.35 | 0.00 | **0.00** |  | 0.28 | 0.87 | 1.00 |
| E | Lysozyme Ctype1 | 168.33 | 0.00 | **0.00** |  | 28.39 | 0.00 | **0.00** |
| E | Lysozyme-like protein | 0.24 | 0.63 | 1.00 |  | 5.75 | 0.06 | 0.45 |
| E | Lysozyme Ptype | 766.61 | 0.00 | **0.00** |  | 182.36 | 0.00 | **0.00** |
| E | Lysozyme Ctype2 | 329.34 | 0.00 | **0.00** |  | 49.17 | 0.00 | **0.00** |
| E | Lysozyme C type-like protein | 0.05 | 0.82 | 1.00 |  | 12.11 | 0.00 | **0.04** |
| E | Lysozyme Itype1 | 63.58 | 0.00 | **0.00** |  | 11.55 | 0.00 | 0.05 |
| E | Lysozyme Itype2 | 0.09 | 0.77 | 1.00 |  | 4.12 | 0.13 | 0.81 |
| E | Lysozyme Itype3 | 60.57 | 0.00 | **0.00** |  | 0.93 | 0.63 | 1.00 |
| E | Lysozyme Ctype3 | 877.13 | 0.00 | **0.00** |  | 10.38 | 0.01 | 0.08 |
| E | Metacaspase-like cysteine peptidase1 | 0.46 | 0.50 | 1.00 |  | 5.48 | 0.06 | 0.50 |
| E | Metacaspase-like cysteine peptidase2 | 58.00 | 0.00 | **0.00** |  | 4.94 | 0.08 | 0.62 |
| E | Asparaginyl endopeptidase-like cysteine peptidase1 | 34.90 | 0.00 | **0.00** |  | 9.05 | 0.01 | 0.13 |
| E | Lysosomal Pro-X carboxypeptidase1 | 7.42 | 0.01 | 0.09 |  | 0.54 | 0.76 | 1.00 |
| E | Prolixicin antimicrobial protein1 | 24.38 | 0.00 | **0.00** |  | 343.25 | 0.00 | **0.00** |
| E | Transferrin1 | 87.80 | 0.00 | **0.00** |  | 17.03 | 0.00 | **0.00** |
| E | Transferrin2 | 0.00 | 0.96 | 1.00 |  | 0.37 | 0.83 | 1.00 |
| E | Transferrin3 | 0.62 | 0.43 | 1.00 |  | 56.52 | 0.00 | **0.00** |
| E | Termicin1 | 19.25 | 0.00 | **0.00** |  | 161.17 | 0.00 | **0.00** |
| E | Cysteine-rich protein 1 | 17.21 | 0.00 | **0.00** |  | 50.49 | 0.00 | **0.00** |
| E | Cysteine-rich protein 2 | 0.11 | 0.74 | 1.00 |  | 4.71 | 0.10 | 0.66 |
| E | Cysteine-rich protein 3 | 4.48 | 0.03 | 0.31 |  | 2.18 | 0.34 | 1.00 |
| E | Cysteine-rich protein 4 | 0.04 | 0.83 | 1.00 |  | 1.76 | 0.41 | 1.00 |
| E | Cysteine-rich protein 5 | 18.06 | 0.00 | **0.00** |  | 10.34 | 0.01 | 0.08 |
| E | Cysteine-rich protein 6 | 32.09 | 0.00 | **0.00** |  | 4.73 | 0.09 | 0.66 |
| E | Cysteine-rich protein 7 | 0.01 | 0.91 | 1.00 |  | 3.90 | 0.14 | 0.87 |
| E | Cysteine-rich protein 8 | 0.92 | 0.34 | 1.00 |  | 31.39 | 0.00 | **0.00** |
| E | Cysteine-rich protein 9 | 3.83 | 0.05 | 0.42 |  | 10.63 | 0.00 | 0.07 |
| E | Cysteine-rich protein 10 | 13.28 | 0.00 | **0.01** |  | 2.17 | 0.34 | 1.00 |
| E | Cysteine-rich protein 11 | 24.34 | 0.00 | **0.00** |  | 26.64 | 0.00 | **0.00** |
| E | Cysteine-rich protein 12 | 18.25 | 0.00 | **0.00** |  | 345.10 | 0.00 | **0.00** |
| E | Cysteine-rich protein 13 | 32.09 | 0.00 | **0.00** |  | 4.73 | 0.09 | 0.66 |
| E | Cysteine-rich protein 14 | 214.99 | 0.00 | **0.00** |  | 109.20 | 0.00 | **0.00** |
| E | Ferritin1 | 0.00 | 1.00 | 1.00 |  | 28.76 | 0.00 | **0.00** |
| E | Ferritin2 | 6.86 | 0.01 | 0.11 |  | 2.24 | 0.33 | 1.00 |
| E | Ferritin3 | 1.28 | 0.26 | 1.00 |  | 3.04 | 0.22 | 1.00 |
| E | Ferritin4 | 1.03 | 0.31 | 1.00 |  | 4.17 | 0.12 | 0.79 |
| E | Melanotransferrin1 | 2.89 | 0.09 | 0.56 |  | 1.66 | 0.44 | 1.00 |
| E | Venom allergen1 | 22.77 | 0.00 | **0.00** |  | 0.93 | 0.63 | 1.00 |
| E | Thaumatin-like protein1 | 15.15 | 0.00 | **0.00** |  | 2.97 | 0.23 | 1.00 |

Comparison of normalized counts per million (CPM) between reproductive statuses (reproductive castes: alates, young PKs and PQs, and mature PKs and SQs; neuter castes: soldiers and workers) and between sexes was conducted by edgeR package. Bold letters mean significant differences (FDR < 0.05). LR: likelihood ratio, FDR: false discovery rate, PRP: pattern recognition protein, S: signalling protein, E: Effector.
